# Alkene hydrosilylation catalyzed by easily assembled Ni(ii)-carboxylate MOFs†
†Electronic supplementary information (ESI) available. See DOI: 10.1039/c9sc00126c


**DOI:** 10.1039/c9sc00126c

**Published:** 2019-02-25

**Authors:** Zhikun Zhang, Lichen Bai, Xile Hu

**Affiliations:** a Laboratory of Inorganic Synthesis and Catalysis , Institute of Chemical Sciences and Engineering , École Polytechnique Fédérale de Lausanne (EPFL) , ISIC-LSCI , Lausanne 1015 , Switzerland . Email: xile.hu@epfl.ch

## Abstract

We report the first Ni MOF catalysts for anti-Markovnikov hydrosilylation of alkenes. These catalysts are bench-stable and easily-assembled from simple Ni salts and carboxylic acids. The best catalyst gives turnover numbers up to 9500 and is robust even after 10 recycling runs. The catalyst can be applied for the hydrosilylation of a wide range of alkenes, achieving good synthetic utility and functional group tolerance.

## Abstract 2

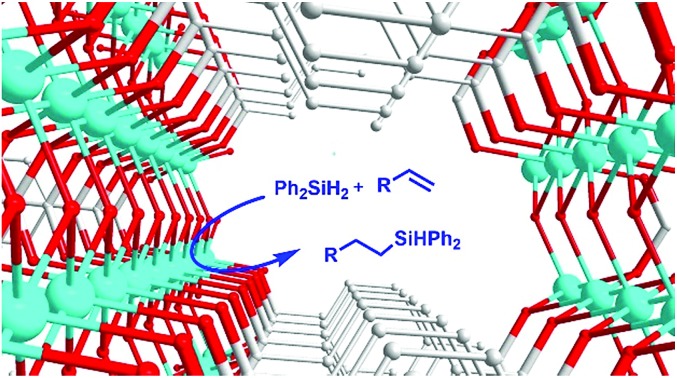
Easily-assembled Ni MOFs are efficient and robust catalysts for alkene hydrosilylation.

## 

Catalytic hydrosilylation of alkenes^1^–^7^ is one of the most important methods to synthesize organosilanes, which are precursors to silicon-based polymers and intermediates in organic synthesis.^8^–^13^ Pt-based catalysts^14^–^16^ are most efficient for hydrosilylation. However, the low abundance and high cost of Pt^17^,^18^ have motivated the development of base metal catalysts. A growing number of Fe, Co, Ni, Cu and Mn complexes have been reported as efficient homogeneous catalysts in alkene hydrosilylation.^4^–^7^,^19^–^33^ Through rational ligand development, it is possible to tune the reactivity,^20^ regioselectivity,^4^,^31^ and even enantioselectivity^6^ of base metal catalysts. Despite this progress, significant improvement of base metal catalysts is still required for practical applications. Designer ligands and complexes require multi-step synthesis and can be expensive. Many reported catalysts are air and moisture sensitive, making them difficult to handle. More importantly, the separation of homogeneous catalysts from the reaction mixture can be problematic and costly.

Heterogenized catalysts based on metal–organic frameworks (MOFs) exhibit both the tunability of homogeneous catalysts and the stability and practicality of heterogeneous catalysts.^34^–^41^ In pioneering work, Lin and co-workers incorporated active metal complexes into MOFs for alkene hydrosilylation.^42^,^43^ Nevertheless, only one base metal MOF catalyst, the Fe-containing 2D Hf-MOF, had been developed for alkene hydrosilylation,^43^ and it was applied for the hydrosilylation of only four simple alkenes. This Fe MOF catalyst was prepared by a post-synthetic metalation strategy. However, the synthesis of ligands containing two types of orthogonal coordination groups, necessary for this strategy, can be challenging. An analogous strategy was adapted for the development of Co-coordination polymer catalysts for alkyne hydrosilylation and alkene hydroborylation.^44^,^45^

Here we report an alternative MOF system that is easy to prepare, is stable, and exhibits broad substrate scope for alkene hydrosilylation. Our catalysts are based on Ni carboxylate MOFs,^46^–^48^ which can be prepared by mixing simple Ni salts with di- or polycarboxylate ligands.^49^ The Ni centers at the nodes of these MOFs can be accessible reaction centers.^50^ Some Ni carboxylate MOFs are reported to catalyze organic reactions^51^–^54^ as well as electrochemical oxygen evolution.^55^ However, none were known to catalyze alkene hydrosilylation prior to this work. In fact, these Ni MOFs are the first Ni-based heterogeneous catalysts for alkene hydrosilylation.

We started with a Ni MOF of the benzenedicarboxylic acid ligand (BDC, **L1**), which was characterized previously.^55^ This MOF is made of ultrathin 2-dimensional nanosheets, in which the hydroxyl-coordinated nickel center could act as the active site for hydrosilylation. In initial exploration, we also screened analogous compounds assembled from three other commonly used di- and tri-carboxylate ligands (**L2–L4**, Table 1). The hydrosilylation of *n*-decene (**1a**) by Ph_2_SiH_2_ (**2**) was used as a test reaction. A robustness test consisting of three consecutive runs was employed to identify the most robust catalysts (Table 1). It was noted that with the same nickel salt and ligands, different synthetic procedures gave catalysts with different activities (see Section 3.1 and 3.2 in the ESI†). For each metal ligand combination, results from the best catalysts are shown in Table 1. To our satisfaction, all four ligands (**L1–L4**) gave Ni-MOFs with high catalytic efficiency and robustness (Table 1, entries 1–4). Biphenyl-4,4′-dicarboxylic acid (**L4**) was slightly better than the other three ligands (**L1–L3**) (Table 1, entry 4). With a simple nickel salt without a ligand, a moderate yield was obtained in the first run (Table 1, entry 5). However, numerous black particles were formed during the reaction, which were presumably nickel nanoparticles. Very low to negligible yields were obtained in the 2^nd^ and 3^rd^ runs. We also tested some other reported nickel MOFs,^51^,^54^,^56^–^58^ such as [Ni_2_-(BDC)_2_-DABCO], Ni-HKUST-1, Ni-MOF-74, [Ni_3_(2,6-NDC)_3_(bipy)_1.5_] and [Ni(C_8_H_4_O_4_)(C_5_H_5_NO)] (Table 1, entry 6–10). These Ni MOFs all catalyzed the hydrosilylation with good to excellent yields in the first run; however, they were less robust, and the yields dropped significantly in the 2^nd^ and 3^rd^ runs. Recently many cobalt, iron and manganese complexes were developed for homogeneous hydrosilylation of alkenes.^4^–^7^,^59^ We attempted to prepare Co, Fe, and Mn-MOFs by combining a Co, Fe, or Mn salt with **L1** under conditions similar to those for the synthesis of the **Ni-L1** MOF. However, these compounds were poor catalysts even in the first run (Table 1, entries 11–13). Overall the results in Table 1 indicated that Ni carboxylate MOFs were the most efficient and robust catalysts for alkene hydrosilylation.

**Table 1:** Screening of robust MOF catalysts for the hydrosilylation of *n*-decene^*a*^

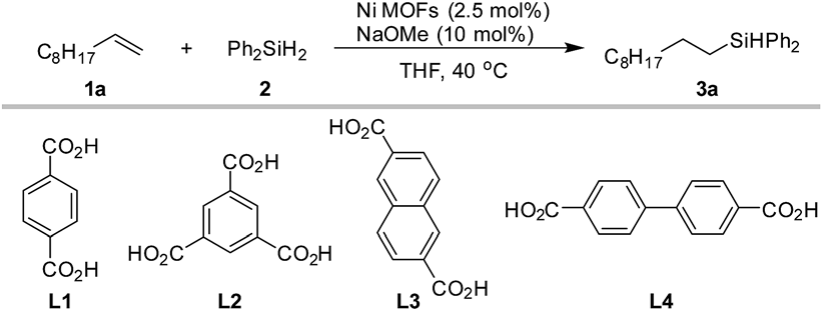				
Entry	Catalysts	1^st^	2^nd^	3^rd^
1	**Ni-L1-1**	85	82	78
2	**Ni-L2-1**	85	85	88
3	**Ni-L3-1**	88	71	80
4	**Ni-L4-1**	87	82	92
5	NiCl_2_·6H_2_O	79	10	3
6	[Ni_2_-(BDC)_2_-DABCO]	90	84	77
7	Ni-HKUST-1	67	14	6
8	Ni-MOF-74	71	52	17
9	[Ni_3_(2,6-NDC)_3_(bipy)_1.5_]	62	86	15
10	[Ni(C_8_H_4_O_4_)(C_5_H_5_NO)]	82	15	8
11^*b*^	Co-L1-1	<1	nd	nd
12^*b*^	Fe-L1-1	14	nd	nd
13^*b*^	Mn-L1-1	2	nd	nd

^*a*^Conditions: 0.2 mmol **1a**, 0.21 mmol diphenylsilane **2**, 2.5 mol% catalyst and 10 mol% NaOMe, 2 mL tetrahydrofuran, 40 °C reaction temperature. The yields were obtained through GC with dodecane as the internal standard, the results are the average values of two parallel experiments.

^*b*^The scale was 0.1 mmol. nd = not determined.

As **Ni-L4-1** was the optimized catalyst, it was subjected to further characterization (Fig. 1a–c and Section 4.1, ESI†). According to the powder X-ray diffraction (PXRD) pattern (Fig. 1a), the catalyst is crystalline. The Scanning Electron Microscope (SEM) image showed the sheet-like morphology of the catalyst (Fig. 1c), similar to the previously reported **Ni-L1-1**.^55^ The Transmission electron microscope (TEM) image indicated the thin nature of the catalyst (Fig. 1b). Element mapping showed that Ni and O were uniformly distributed throughout the sample (Fig. S1, ESI†), and X-ray photoelectron spectroscopy (XPS) indicated that Ni was coordinated to carboxylate ligands (Fig. S2, ESI†). According to N_2_ adsorption experiments (Fig. S3, ESI†), the surface area was about 120 m^2^ g^–1^, and the pore size distribution was narrow and uniform. All pores were between 3.0 nm and 4.0 nm. Thermogravimetry Analysis (TGA) suggested that the catalyst was thermally stable up to 400 °C (Fig. S4, ESI†). The Infra-red (IR) spectrum suggested the existence of Ni–OH moieties (Fig. S5, ESI†).

**Fig. 1:**
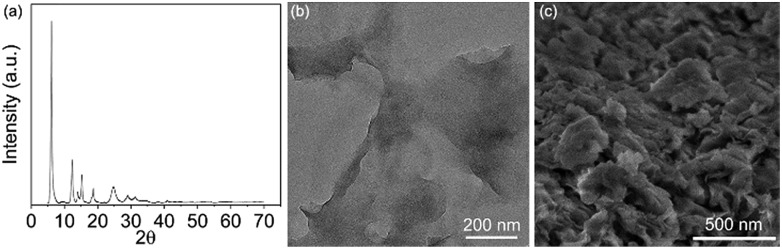
(a) PXRD pattern of **Ni-L4-1**. (b) TEM image of **Ni-L4-1**; (c) SEM image of **Ni-L4-1**.

The robustness of **Ni-L4-1** was further tested in 10 recycling catalytic runs. The catalyst maintained a similar level of efficiency over 8 runs, and afterwards slightly lower yields were obtained (Fig. 2). Even after 10 runs, the yield was still higher than 60%. The post-catalytic characterization of the catalyst suggested that the catalyst remained in its initial state after multiple catalytic runs (Section 4.2, ESI†). These results suggest that the catalyst is robust.

**Fig. 2:**
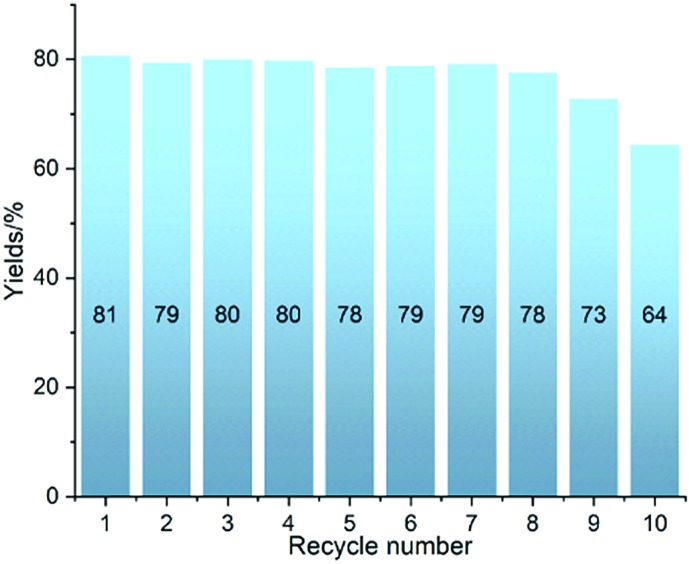
Testing the robustness of **Ni-L4-1** in ten recycling experiments with **1a** and **2** as substrates.

Although the results from the recycling experiments were consistent with the catalyst being heterogeneous, they were not proof. To further probe whether the catalysis was homogeneous or heterogeneous in nature, we conducted a filtration experiment (Scheme 1 and Section 5, ESI†). After 20 minutes of catalytic hydrosilylation, the yield was 60%. When the reaction was continued for 1.5 h, the final yield was 87%. When the reaction medium was centrifuged to separate the solution and solid phase, and the solution phase was allowed to react for another 1.5 h, no further hydrosilylation beyond the initial 60% was observed. The possibility that a homogeneous catalyst species was deactivated by centrifugation could be ruled out because the solid phase was still active. This result gives further support that **Ni-L4-1** is a heterogeneous catalyst.

**Scheme 1:**
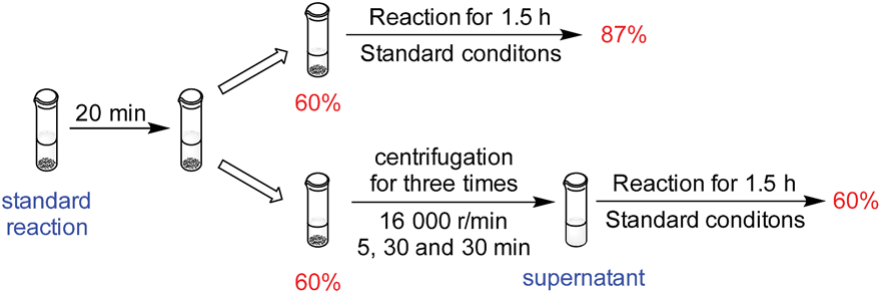
Probing the heterogeneous nature of **Ni-L4-1**.

The scope of this heterogeneous hydrosilylation was then explored using **Ni-L4-1** as the catalyst (Table 2). Generally, anti-Markovnikov selectivity was observed. Unhindered alkyl alkenes were hydrosilylated in high yields (**1a–1e**). The internal alkenyl carbon could be substituted by a secondary (**1f**) or tertiary (**1g**) alkyl group. When the substrate contained both an internal (cyclic) and a terminal C–C double bond (**1h**), hydrosilylation was selective on the terminal position. Functional groups such as chloro (**1i**), trifluoromethyl (**1j**), cyanide (**1k**), and silyl (**1l–1n**) groups were readily tolerated. Hydroxyl and carbonyl groups needed to be protected (**1o** and **1p**). The ester group (**1q** and **1r**) was compatible. Although nickel-catalyzed ring-opening of epoxide was reported,^60^ epoxide was tolerated by this method (**1s** and **1t**). Allylic ether (**1u**) and allylic amine (**1v** and **1w**) were also suitable substrates. Styrene (**1x**) was hydrosilylated at both the α and β positions, due to stabilization of benzyl intermediates. Anti-Markovnikov selectivity was again obtained for α-substituted styrenes (**1y** and **1z**). For an acyclic internal alkene, 2-octene, the substrate was first isomerized to a terminal alkene before hydrosilylation to give the terminal silane (**3b′**). Similar results were previously reported in homogeneous and nanoparticle catalysis.^25^ Normal hydrosilylation occurred on cyclic alkenes such as norbornene (**1ac**) and cyclopentene (**1ad**) as isomerization was either impossible or indistinguishable. When 1-allylpyrene (**1aa**), a rather big molecule, was used as the substrate, nearly no product (**3aa**) was obtained. Comparing the results using **1e** and **1aa** as substrates suggests that catalysis is sensitive to the size of the substrate, which indicates that the reaction occurs mostly inside the mesopores.

**Table 2:** Substrate scope^*a*^ ^*b*^

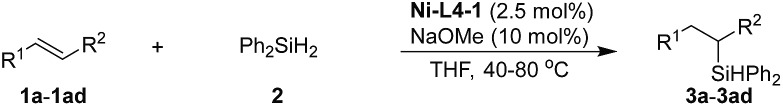
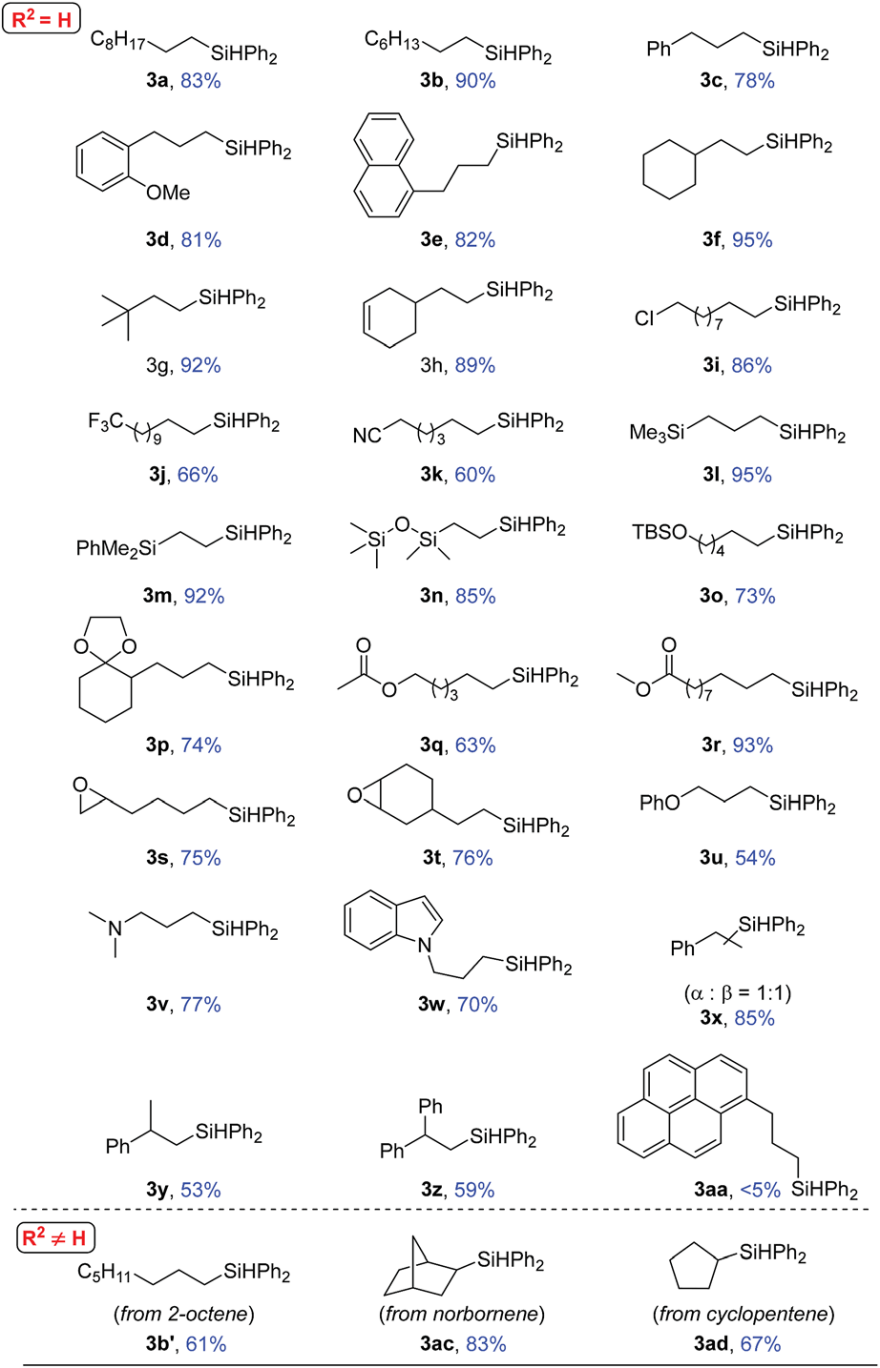

^*a*^Conditions: 0.5 mmol (1.0 eq.) alkenes and 0.525 mmol (1.05 eq.) diphenylsilane, 2.5 mol% **Ni-L4-1**, 10 mol% NaOMe, 2.0–5.0 mL tetrahydrofuran (THF), reaction temperature: 40–80 °C, reaction time: 2–84 hours.

^*b*^The yields are isolated yields after column chromatography or preparative thin-layer chromatography with silica gel.

To show the potential of the current method in practical applications, a 150 mmol scale synthesis was carried out using **1b** and **2** as substrates (Scheme 2a). 0.01 mol% catalyst was sufficient to catalyze the reaction with a 95% yield after 40 hours, corresponding to a turnover number (TON) of 9500. Silanols are key intermediates for the synthesis of silicon-based polymeric material.^61^–^63^ A catalytic amount of sodium hydroxide could be used to transform the silane product (**3b**) into a silanol (**4**) in a one-pot procedure (Scheme 2b). The latter step does not require nickel catalysis (Section 3.5, ESI†).^64^

**Scheme 2:**
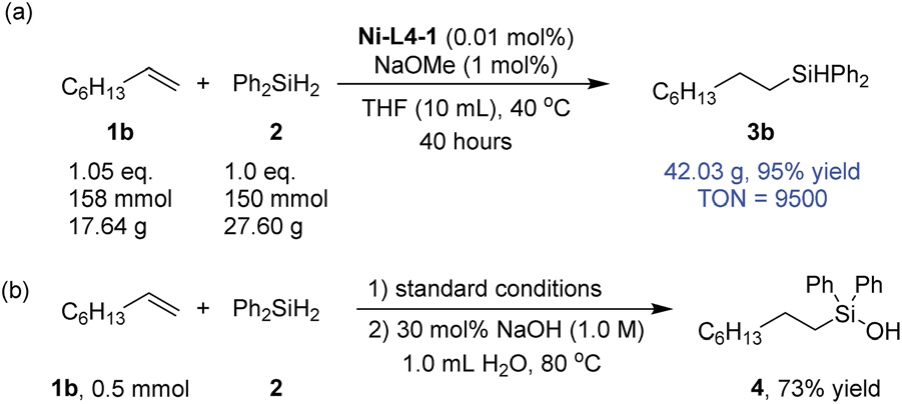
(a) A large-scale hydrosilylation reaction; (b) one-pot hydrosilylation and silanol formation.

Several experiments were conducted to obtain preliminary insights into the mechanism of the reaction. When a deuterated silane, Ph_2_SiD_2_ (**D-2**) was used for the hydrosilylation of **1d**, deuterium scrambling was observed in the α, β, and γ-carbon positions of **D-3d** (Scheme 3a). In homogeneous Ni-catalyzed hydrosilylation of alkenes, nickel hydride (Ni–H)^65^ species are commonly proposed as the key intermediate.^30^,^66^–^69^ The result in Scheme 3a suggests that an analogous Ni–H species in the Ni-MOF catalyst is an intermediate. When the final reaction mixture after hydrosilylation was quenched with water, hydrogen evolution was observed (Scheme 3b). An analogous quenching of the reaction mixture without the alkene substrate yielded copious H_2_. These results suggest the Ni–H species as the resting state of the catalyst. Although the crystal structure of **Ni-L4-1** is inaccessible due to poor crystallinity, we suspect the local structure of Ni centers to be similar to the Ni centers in **Ni-L1-1**, which is supported by their similar IR and UV-vis spectra (Fig. S5 and S6, ESI†). The active site, thus, is a Ni center with an exchangeable ligand such as hydroxyl. Upon silane addition, this Ni–OH group is converted to the Ni–H intermediate. There are two possible roles for NaOMe: to activate silane by forming a 5-coordinate silicon intermediate prone to hydride transfer,^32^ or to form a Ni–OMe species that reacts with silane to form the Ni–H.^30^ Finally, to probe whether the reaction occurred exclusively on a defect site rather than a node site, a defect poison, NaSCH_3_, was used as an additive in the hydrosilylation (Scheme 3c). In the presence of NaSCH_3_, the hydrosilylation still proceeded smoothly to give a yield of 89%. This result suggests that the defect sites are not the major reaction sites.

**Scheme 3:**
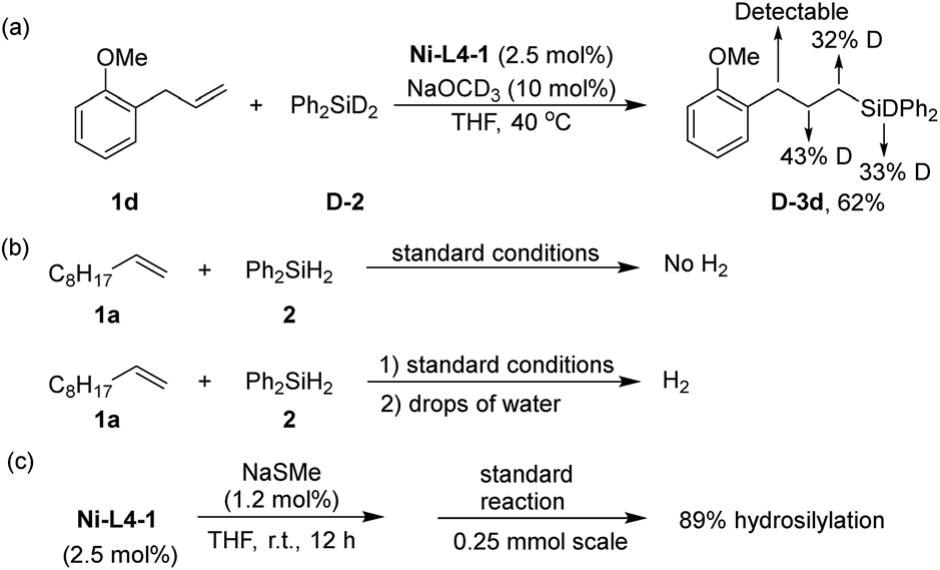
Mechanism study: (a) isotope labelling experiment; (b) quenching by water; the quench was after full conversion of silane in the hydrosilylation reaction; (c) catalyst poison test.

## Conclusions

In summary, we have developed efficient and robust MOF catalysts for the hydrosilylation of alkenes. The catalysts are based on Ni carboxylate MOFs, which can be easily assembled from readily available Ni salts and carboxylic acids. The best catalyst, **Ni-L4-1**, shows high activity even after 10 recycling experiments. TONs of up to 9500 are achieved, the highest for a base metal heterogeneous catalyst. This catalyst can be applied for the hydrosilylation of a large number of alkenes, with good functional group tolerance. This work demonstrates the synthetic utility of base metal MOF catalysts for alkene hydrosilylation.

## Conflicts of interest

The authors declare no conflict of interest.

## Supplementary Material

Supplementary information
