# Supplementary material for: Alkene hydrosilylation catalyzed by easily assembled Ni(ii)-carboxylate MOFs
Source: Chem Sci. 2019 Feb 25;10(13):3791–5. doi: 10.1039/c9sc00126c (PMC6446965; doi:10.1039/c9sc00126c)
Supplement: Supplementary file 1 [file SC-010-C9SC00126C-s001.pdf]

Electronic Supplementary Material (ESI) for Chemical Science.
This journal is © The Royal Society of Chemistry 2019

Supplementary Information

Alkene Hydrosilylation Catalyzed by Easily Assembled Ni(II)-Carboxylate MOFs

Zhikun Zhang, Lichen Bai, and Xile Hu*

Laboratory of Inorganic Synthesis and Catalysis, Institute of Chemical Sciences and Engineering, École Polytechnique Fédérale de Lausanne (EPFL), ISIC-LSCI, Lausanne 1015, Switzerland

Contents

1. General 2

2. Synthesis of substrates 3

3. Base metal MOF catalyzed hydrosilylation of alkenes 3

4. Characterization of Ni-L4-1 5

5. Probing heterogeneous nature 10

6. Mechanism study 10

7. Spectral data 11

8. Reference 17

9. ^{1}H, ^{13}C and ^{29}Si spectra 18

1. General

All the reactions were performed under an inert N_2 (g) atmosphere and dry reaction tube. THF was purified using a two-column solid-state purification system (Innovative Technology, NJ, USA) and transferred to a nitrogen filled glovebox without exposure to air. $CDCl_3$ were purchased from ARMAR AG and used directly. Unless otherwise noted, all other reagents and starting materials were purchased from commercial sources and used without further purification. ^1H and ^{13}C NMR spectra were recorded at 293 K on Bruker Advance 400 spectrometers. ^1H NMR chemical shifts were referenced to residual solvent as determined relative to Me_4Si (δ = 0 ppm) or $CHCl_3$ (δ = 7.26 ppm). The ^{13}C (CPD) chemical shifts were reported in ppm relative to the carbon resonance of $CDCl_3$ (77.16 ppm). The data for NMR spectra were reported as follows: chemical shifts (δ) were reported in ppm, and coupling constants (J) were in Hertz (Hz). The following abbreviations were used to explain the multiplicities: s = singlet, d = doublet, t = triplet, q = quartet, m = multiplet, br = broad. GC measurement was conducted on a Perkin-Elmer Clarus 400 GC with a FID detector. GC-MS measurements were conducted on an Agilent Technologies 7890A GC system equipped with a 5975C mass detector. High-resolution mass (HRMS) measurements were conducted at the EPFL ISIC mass spectrometry service with a Micro Mass QTOF with atmospheric pressure photoionization (APPI) ionization source. Elemental analyses were performed on a Carlo Erba EA 1110 CHN instrument. Powder X-ray diffraction measurements were carried out on Malvern Panalytical Aeris Cement Edition with monochromatic $CuK_{\alpha1,2}$ radiation and a PIX^{1D} detector or X'Pert Philips diffractometer in Bragg-Brentano geometry with monochromatic $CuK_{\alpha1,2}$ radiation and a fast Si-PIN multi-strip detector (0.1540 nm). Transmission electron microscope (TEM) and High-angle annular dark-field scanning transmission electron microscope (HAADF-STEM) images were collected on a FEI Tecnai Osiris TEM equipped with high-brightness field emission gun (XFEG). Samples for TEM were prepared by drying a drop of diluted ethanol dispersion of the samples onto a copper grid covered by ultrathin carbon membrane. Energy-dispersive X-ray spectroscopy (EDX) mapping images were taken under a scanning TEM (STEM) modal. TGA analyses were performed on a TGA 4000 from Perkin Elmer in a 20 $mL \cdot min^{-1}$. The ramping rate of temperature was 10 $°C \cdot min^{-1}$. Scanning electron microscopy (SEM) images were taken using a Zeiss Merlin Microscope operated at 2 kV and equipped with an Inlens secondary electron detector. N2 adsorption-desorption experiment was carried out with Quantachrome autosorb iQ instrument. The IR was tested with Varian 800 FT-IR Scimitar Series. UV-vis was tested with Shimadazu UV-3600Plus UV-VIS-NIR machine. sssThe products were isolated with preparation thin-layer chromatography with TLC Silica gel 60 F_{254} from Merck KGaA, Darmstadt, Germany or column chromatography filled with silica gel from SiliaFlash® P60 (40-60 μm, 230-400 mesh). Diphenylsilane and Biphenyl-4, 4'-dicarboxylic acid was from TCI Chemicals. Sodium methoxide was from Sigma-Aldrich. Nickel (II) chloride hexahydrate was from AlfaAesar.

2. Synthesis of substrates

Substrates **1j**[1], **1o**[2], **1p**[3], **1w**[4] and **1ad**[5] were prepared according to related reported procedures.

3. Base metal MOF catalyzed hydrosilylation of alkenes

3.1 Synthesis of MOFs[6]

A typical procedure for the synthesis of Ni-L4-1: 4,4'-Biphenyldicarboxylic acid (0.375 mmol, 91mg) was added to a mixture of DMF (16 mL), ethanol (1 mL) and water (1 mL) in a 50 mL plastic tube. Then 0.375 mmol $NiCl_2{\cdot}6H_2O$ (89.2 mg) was added. The reaction mixture was stirred for five minutes. Then 0.4 mL triethylamine was added, and then the mixture was sonicated with an ultrasonic cleaner for eight hours. During the process, the bath temperature was controlled between 0–25 °C through addition of ice. The final reaction mixture was centrifuged at 8000 rpm for 15 minutes, then washed with 8 mL water, DMF and ethanol twice. The powder was collected.

Other MOF samples were prepared in a similar manner, except some variations in conditions (Table S1). The isolation process was the same as for **Ni-L4-1**.

Table S1. Summary of the synthetic conditions for different MOFs.

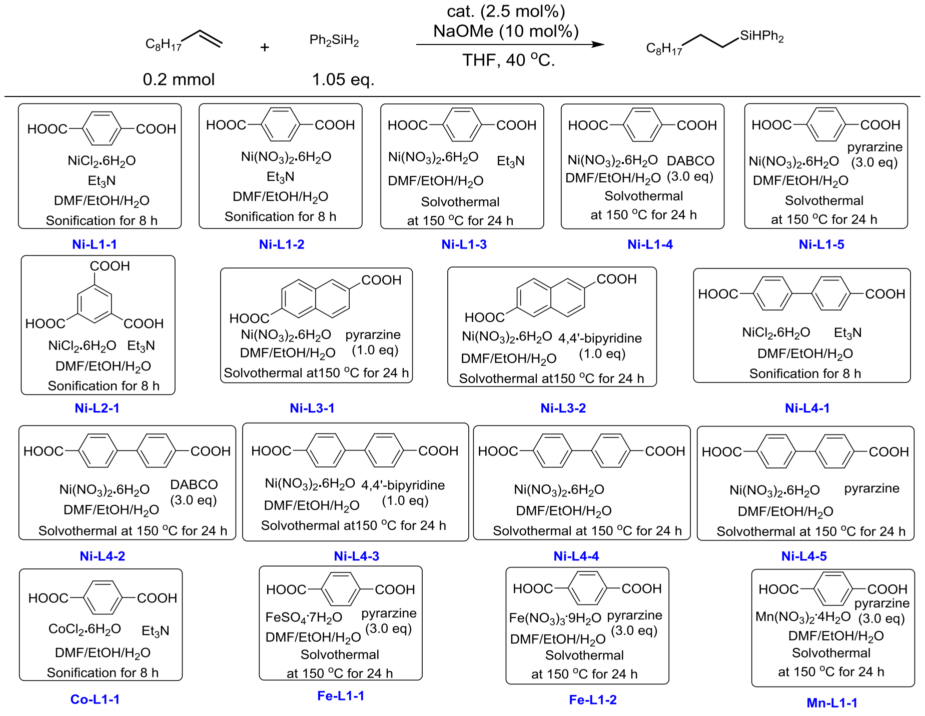

3.2 Screening of catalytic efficiency

General procedures: 2.5 mol% catalyst and corresponding amount of NaOMe were added to a dry vial with a magnetic stir. Then the vial was transferred to a nitrogen filled glovebox. 2 mL THF was added to the vial by a pipette, followed by addition of 0.2 mmol 1-decene. Then the mixture was stirred for 1 minute. 0.21 mmol diphenylsilane was then added. The vial was taken out of the glovebox and allowed to react at 40 °C under a 600 rpm stirring rate for the 12 hours. Then the reaction mixture was quenched by adding 2 mL diethyl ether. Dodecane was then added and the mixture was stirred for five minutes.

Then the mixture was transferred to a centrifuge tube and centrifuged at 12000 rpm for 5 minutes to isolate the catalyst. The supernatant was analysed by GC to give the yields of the hydrosilylation. Then the catalyst was washed with 2 mL diethyl ether for two times. At last, the catalyst was transferred to the same reaction vial, dried in vacuum for 1 hour, and reused. NaOMe and substrates were added for the next run. Three runs were made for each catalyst, unless otherwise noted. The results are shown in Table S2 and Table 1, main text.

We tried other silanes such as $(EtO)_3SiH$ but the yields were poor. For reactions that did not go well, we observed disproportionation products due to base-mediated disproportionation. For example, with $(EtO)_3SiH$ we saw $(EtO)_4Si$ in GC-MS.

Table S2. Screening of MOF catalysts for hydrosilylation of n-decene.

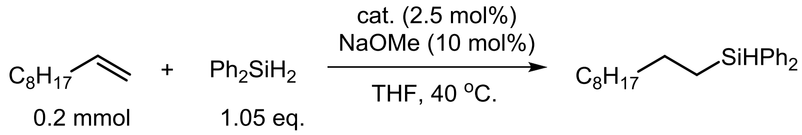

Entry	Cat.	1st use	2nd use	3rd use	Entry	Cat.	1st use	2nd use	3rd use
1	**Ni-L1-1**	85	82	78	8	**Ni-L3-2**	88	71	80
2	**Ni-L1-2**	83	89	87	9	**Ni-L4-1**	87	82	92
3	**Ni-L1-3**	87	67	17	10	**Ni-L4-2**	92	85	28
4	**Ni-L1-4**	88	86	84	11	**Ni-L4-3**	93	17	8
5	**Ni-L1-5**	91	8	14	12	**Ni-L4-4**	90	78	14
6	**Ni-L2-1**	85	85	88	13	**Ni-L4-5**	88	11	5
7	**Ni-L3-1**	97	19	7					

3.3 Robust test by 10 recycling experiments

General procedures: **Ni-L4-1** (3.7 mg, 0.0125 mmol, 2.5 mol%) and NaOMe (2.7 mg, 0.05 mol, 10 mol%) were added to a dry vial with a magnetic stir. Then the vial was transferred to a nitrogen filled glovebox, 2 mL THF was added to the vial with pipette, followed by adding of 1-decene (70.1 mg, 0.5 mmol, 1.0 eq.). Then the mixture was stirred for 1 minute. Diphenylsilane (0.525 mmol, 96.7) was then added. The vial was taken out of the glovebox and allowed to react at 40 °C under a 450 rpm stirring rate for 2 hours. Then the reaction mixture was quenched by adding 2 mL diethyl ether, then dodecane was added and the mixture was stirred for five minutes. Then the mixture was transferred to a centrifuge tube and centrifuged at 12000 rpm for 5 minutes to isolate the catalyst. The supernatant was analysed by GC to give the yields of the hydrosilylation. Then the catalyst was washed with 2 mL THF for two times. At last, the catalyst was transferred to the same reaction vial, dried in vacuum for 1 hour, and reused. NaOMe and substrates were added for the next run.

Table S3. Yields of hydrosilylation in 10 recycling experiments (2 trials) using **Ni-L4-1** as catalyst

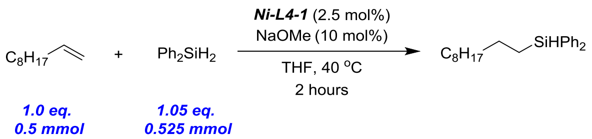

Recycling number	1	2	3	4	5	6	7	8	9	10
sample 1	89	88	88	86	85	86	87	86	82	81
sample 2	73	70	72.3	74	72	72	71	69	63	48
Average (%)	81	79	80	80	78	79	79	78	73	64

3.4 Exploration of scope of hydrosilylation

General procedures: **Ni-L4-1** (3.7 mg, 0.0125 mmol, 2.5 mol%) and NaOMe (2.7 mg, 0.05 mol, 10 mol%) were added to a dry vial with a magnetic stir. Then the vial was transferred to a nitrogen filled glovebox. 5 mL THF was added to the vial by a pipette, followed by addition of an alkene (0.5 mmol, 1.0 eq.). Then the mixture was stirred for 1 minute. Diphenylsilane (0.525 mmol, 96.7) was then added. The vial was taken out of the glovebox and allowed to react at 40 °C at a 450 rpm stirring rate. The reaction was stopped after the disappearing of alkene monitored by TLC or GS-MS, and quenched by addition of 2 mL diethyl ether. The mixture was filtered through a pad of silica gel eluted by diethyl ether. The solvent was removed using a rotary evaporator. The hydrosilylation product was isolated by column chromatography or preparation thin-layer chromatography with silica gel. For substrate **1y**, **1aa** and **1ac**, the reactions were conducted at 60 °C in 2 mL THF. For substrate **1z** and **1ab**, the reactions were conducted at 80 °C in 2 mL THF.

3.5 One-pot hydrolysis of the produced silane

General procedures: A reaction with **1b** as the substrate was set up according to the above mentioned standard conditions. After two hours, the cape of the vial was opened, and 1.0 mL water was added, followed by addition of a 150 µL NaOH (1.0 M, 30 mol%) aqueous solution. H_2 gas was evolved immediately. The mixture was then allowed to react at 80 °C. No silane was detected by GC-MS after 24 hours. The final mixture was diluted by diethyl ether and water, and extracted with diethyl ether to give a solution. The final product was isolated by silica chromatography.

Control experiment: To probe the potential role of **Ni-L4-1** in the hydrolysis of the produced silane, a control experiment was conducted. Hydrolysis of silane **3b** was conducted using 30 mol% NaOH only, without the Ni catalyst. Silanol **4** was still produced after 12 hours. This result indicates that hydrolysis does not require a Ni catalyst. A proposed mechanism for the hydrolysis is shown in Scheme S1.

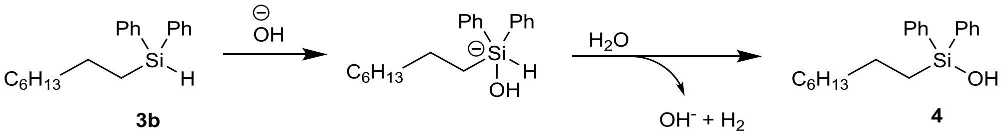

Scheme S1. A proposed mechanism for the hydrolysis of silane

4. Characterization of Ni-L4-1

4.1 Characterization of the as-prepared (pristine) catalyst

4.1.1 High-angle annular dark-field scanning transmission electron microscope (HAADF-STEM) and energy-dispersive X-ray spectroscopy (EDX)

HAADF-STEM and EDX showed the major elements are nickel and oxygen, and they are well distributed in the material (Fig. S1)

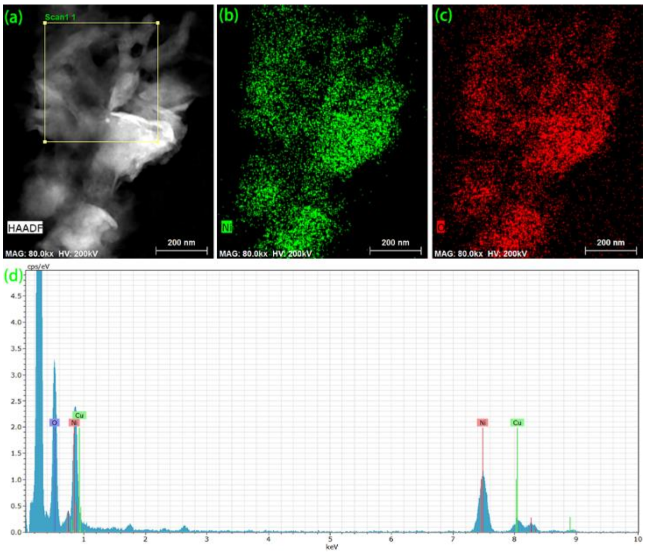

Figure S1. (a): HAADF-STEM image of the pristine catalyst. (b-c) the corresponding EDX mapping. (d) EDX spectrum of the pristine catalyst.

4.1.2 X-ray photoelectron spectroscopy (XPS)

XPS was then used to analyse the catalyst (Fig. S2). The Ni Spectra suggest the successful coordination of carboxylate to nickel, because the binding energies are very similar with those of $Ni(OAc)_2{\cdot}4H_2O$.

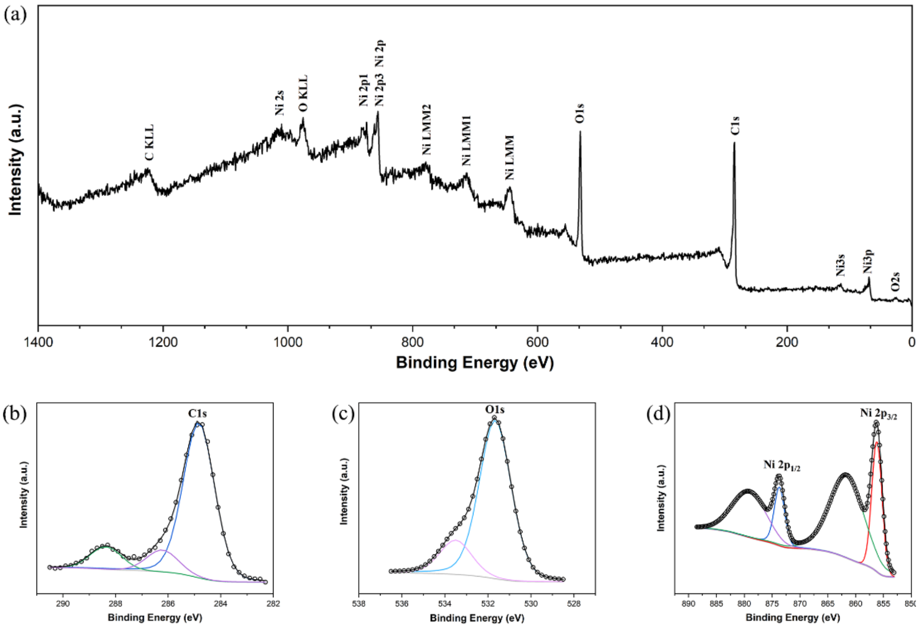

Figure S2. XPS spectra of pristine catalyst. (a): The survey spectrum. (b), C1s spectrum. (c): O1s spectrum. (d): $Ni2p_{1/2}$ and $Ni2p_{3/2}$ spectrum.

4.1.3 N_2 adsorption experiment

The Brunauer-Emmett-Teller (BET) surface area is 123 m^2/g. The pore size distribution is very narrow and uniform. All the pore sizes are between 3.0 nm and 4.0 nm (Fig. S3).

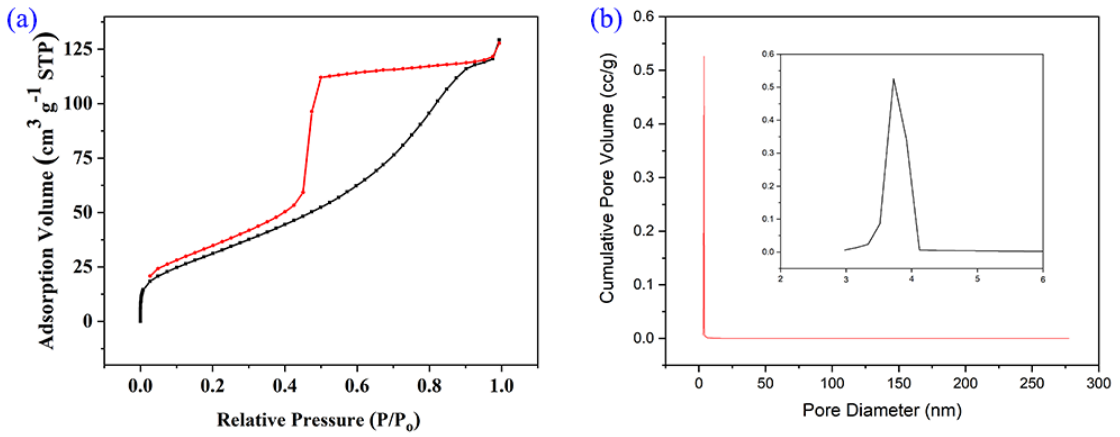

Figure S3. (a). N_2 adsorption-desorption isotherms curve of the pristine catalyst. (b). Pore distribution curve, the inset is the details.

4.1.6 Thermogravimetry Analysis (TGA)

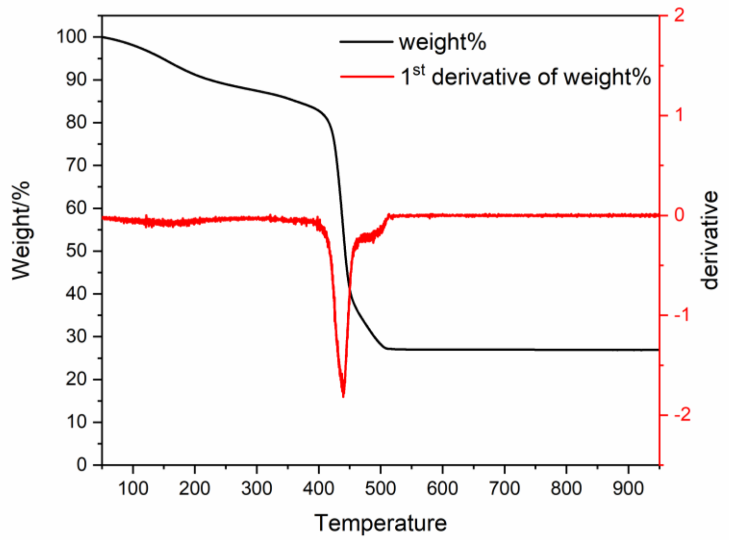

Figure S4. TGA graphs of the pristine catalyst

Thermogravimetry Analysis (TGA) in air (Fig. S4) was used to investigate the stability of the catalyst. The weight loss before 250 ^{o}C should originate from the loss of absorbed solvents. The catalyst began to decompose at 400 ^{o}C, and totally decomposed at 520 ^{o}C to afford nickel oxide.

4.1.7 IR of Ni-L1-1 and Ni-L4-1

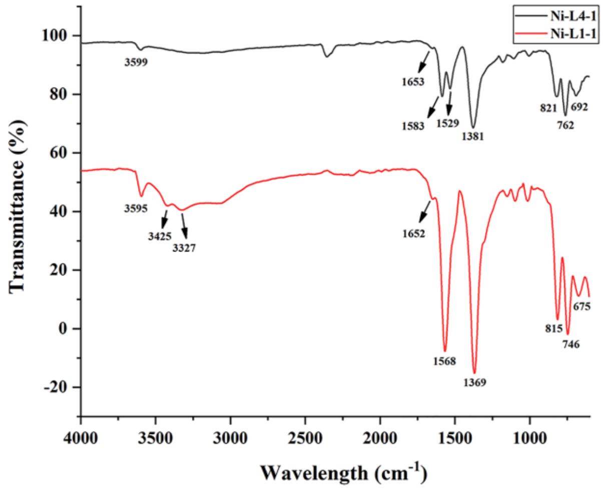

Figure S5. Infrared spectra of **Ni-L4-1** and **Ni-L1-1**.

The IR spectra indicated the existence of Ni-OH moieties. The peaks around 3599 cm^{-1} should belong to O-H vibration, while peaks between 600 cm^{-1} to 850 cm^{-1} should belong to Ni-OH vibration.

4.1.7 UV-Vis of Ni-L1-1 and Ni-L4-1

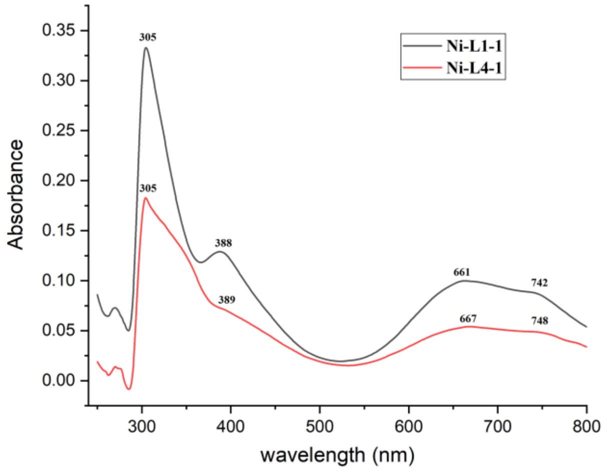

Figure S6. UV-Vis spectra of **Ni-L4-1** and **Ni-L1-1**.

The Ni-based absorbance can be observed between 600 nm and 800 nm. The spectra are similar..

4.2 The post-catalytic characterization

TEM showed the morphology of catalyst did not change, but some sodium salt appeared and accumulated (Fig. S5-S6). HAADF-STEM revealed nickel, oxygen and sodium were homogeneously distributed.

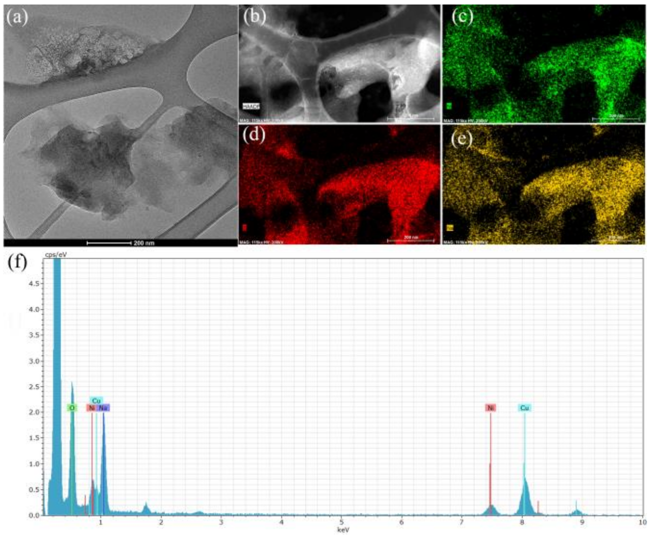

Figure S7. The post-catalytic characterization of the recovered catalyst after 2 runs by microscopy. (a). TEM image. (b.: HAADF-STEM image. (c). Element mapping of Ni; (d) Element mapping of O; € Elemental mapping of Na. (f) The corresponding EDX spectrum.

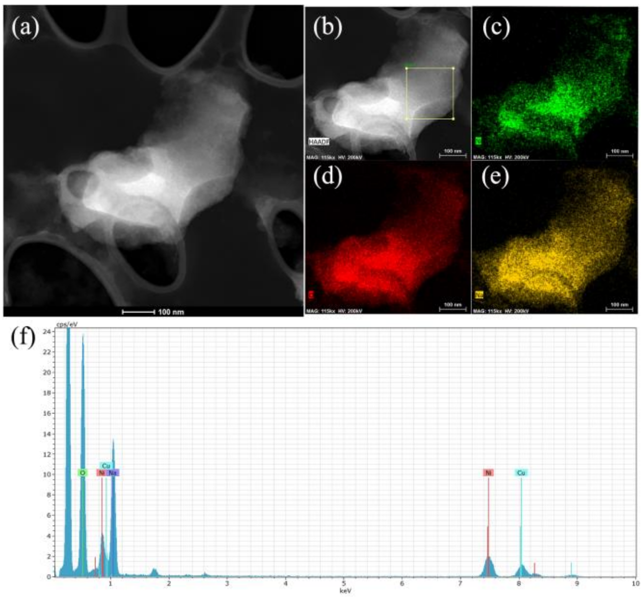

Figure S8. The post-catalytic characterization of the recovered catalyst after 10 runs by microscopy. (a). TEM image. (b.: HAADF-STEM image. (c). Element mapping of Ni; (d) Element mapping of O; € Elemental mapping of Na. (f) The corresponding EDX spectrum.

PXRD pattern of the catalyst after 2 runs showed peaks from sodium formate (Fig. S7b). This sodium salt should derive from sodium methoxide used for the reaction. The pattern was similar to that of the pristine catalyst (Fig. S7a). However, after ten runs, the PXRD pattern became rather different from that of the pristine catalyst, with significant new peaks (Fig. S7c). We suspected that these peaks came from various sodium salts accumulated from the 10 runs. After the catalyst was washed with water, a cleaner PXRD pattern, similar to that of the pristine catalyst, was obtained (Fig. S7d). The intensity of peaks was lower, indicating some loss of catalyst. We tentatively assign this loss to manipulation during 10 recycling runs, although

catalyst deactivation cannot be fully ruled out. The PXRD analysis did rule out the formation of either Ni or NiO_x nanoparticles.

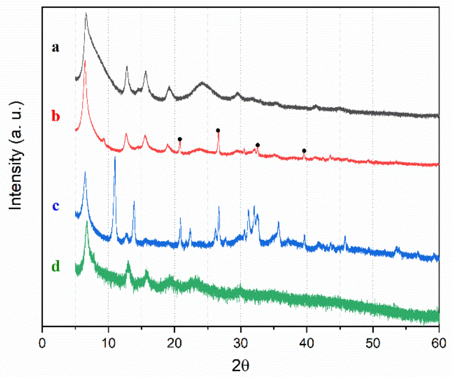

Figure S9. The post-catalytic characterization of the recovered catalysts by PXRD. (a). pristine catalyst; (b). Catalyst after 2 runs; (c). Catalyst after 10 runs. (d). Catalyst after 10 runs which was further washed by water.

5. Probing Heterogeneous nature

General procedure: A reaction was set up according the standard procedures with 50 µL dodecane. It was stopped after 20 minutes and then was transferred to a nitrogen filled glovebox. The mixture was separately by equal portion. One half was allowed to react for another 1.5 hours under normal reaction conditions. The remaining half of the mixture was transferred to two sealable centrifuge tubes. The centrifuge tubes was centrifuged at 16000 rpm for five minutes, and then the half supernatant was transferred to another two sealable centrifuge tubes. The tubes were centrifuged at 16000 rpm for 30 minutes. The above procedure was repeated again. After that, half supernatant was transferred to a dry vial with magnetic stir in a nitrogen filled glovebox, GC was used to analysis the residual supernatant, 60% yield was gave. The vial supernatant was allowed to react at standard conditions for 1.5 hours. After the two reaction finished, we also analysed the yields with GC, we found the reaction with solid species can give 87% yield, however, the reaction of supernatant gave 60% yield. It means only the solid species can catalyze the reaction, so this reaction should be a heterogeneous process.

6. Mechanism study

Procedure for isotope labelling experiment (Scheme 3a, main text): The experiment was carried out using the standard procedure for hydrosilylation of substrate **1d**, except that Ph_2SiD_2 was used instead of Ph_2SiH_2.

Procedure for reaction in Scheme 3b of main text: After two parallel catalytic hydrosilylation reactions as shown in Scheme 3b were completed, the head space of the reaction vials were analysed by GC. Without addition of water, no hydrogen gas was detected. However, if the reaction was quenched with water (several drops), hydrogen evolution was observed.

Procedure for reaction in Scheme 3c of main text (poison probe with $NaSCH_3$: Two parallel vials were charged each with **Ni-L4-1** (1.8 mg, 2.5 mol%) and **$NaSCH_3$** (0.3 mg, 1.2 mol%). Then these two vials were transferred to a glovebox. 2 mL THF was added to each vial, and the mixtures was stirred for 12 hours.

The reaction mixture in each vial was centrifuged to remove the solvent; the solid residue was washed twice with 1.0 mL THF. The solid residue in each vial was then used as catalyst for the hydrosilylation test reaction using **1a** and **2** as substrates. The average yield was 89%.

7. Spectral data

12, 12, 12-Trifluorododec-1-ene (1j)[1]

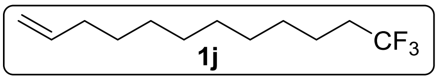

3.0 mmol scale, 79% yield, colourless oil. ^{1}H NMR (CDCl3, 400 MHz) δ 5.87-5.76 (m, 1H), 5.02-4.92 (m, 2H), 2.12-2.00 (m, 4H), 1.59-1.51 (m, 2H), 1.38-1.29 (m, 12H); ^{13}C NMR (CDCl3, 100 MHz) δ 139.3, 127.5 (q, *J* = 276.4 Hz), 114.3, 33.9, 33.9 (q, *J* = 28.3 Hz), 29.5, 29.5, 29.3, 29.2, 29.1, 28.9, 22.0 (q, *J* = 2.8 Hz).

Decyldiphenylsilane (3a)[7]

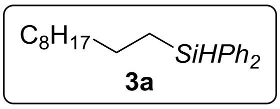

Yield: 134.5 mg, 83%, colorless oil; R_f = 0.57 (hexane); ^{1}H NMR ($CDCl_3$, 400 MHz) δ 7.64-7.62 (m, 4H), 747-7.39 (m, 6H), 4.94 (t, *J* = 3.8 Hz, 1H), 1.58-1.50 (m, 2H), 1.47-1.31 (m, 14H), 1.24-1.19 (m, 2H) 0.96 (t, *J* = 6.7 Hz, 3H); ^{13}C NMR ($CDCl_3$, 100 MHz) δ 135.3, 134.9, 129.6, 128.1, 33.3, 32.1, 29.8, 29.7, 29.5, 29.4, 24.6, 22.9, 14.3, 12.3; ^{29}Si NMR ($CDCl_3$, 79 MHz) δ -13.72.

Octyldiphenylsilane (3b) [8]

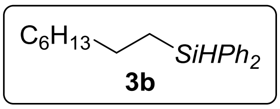

Yield: 132.9 mg, 90%, colorless oil; R_f = 0.54 (hexane); ^{1}H NMR ($CDCl_3$, 400 MHz) δ 7.56-7.54 (m, 4H), 7.40-7.32 (m, 6H), 4.85 (t, *J* = 3.7 Hz, 1H), 1.48-1.42 (m, 2H), 1.37-1.32 (m, 2H), 1.27-1.23 (m, 8H), 1.16-1.11 (m, 2H), 0.86 (t, *J* = 6.8 Hz, 3H); ^{13}C NMR ($CDCl_3$, 100 MHz) δ 135.3, 134.9, 129.6, 128.1, 33.3, 32.0, 29.4, 29.3, 24.5, 22.8, 14.3, 12.3; ^{29}Si NMR ($CDCl_3$, 79 MHz) δ -13.79.

Diphenyl(3-phenylpropyl)silane (3c) [9]

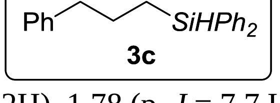

Yield: 117.9 mg, 78%, colorless oil; R_f = 0.33 (hexane); ^{1}H NMR ($CDCl_3$, 400 MHz) δ 7.53-7.51 (m, 4H), 7.37-7.31 (m, 6H), 7.26-7.22 (m, 2H), 7.17-7.11 (m, 3H), 4.86 (t, *J* = 3.7 Hz, 1H), 2.66 (t, *J* = 7.6 Hz, 2H), 1.78 (p, *J* = 7.7 Hz, 2H), 1.29-1.09 (m, 2H); ^{13}C NMR ($CDCl_3$, 100 MHz) δ 142.3, 135.3, 134.5, 129.7, 128.7, 128.4, 128.1, 125.9, 39.4, 26.4, 12.0; ^{29}Si NMR ($CDCl_3$, 79 MHz) δ -13.94.

(3-(2-Methoxyphenyl)propyl)diphenylsilane (3d) [10]

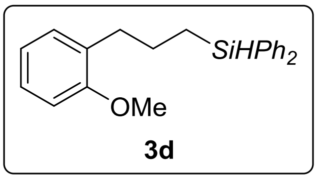

Yield: 134.7 mg, 81%, colorless oil; R_f = 0.22 (hexane : ethyl acetate = 100 : 1); ^{1}H NMR ($CDCl_3$, 400 MHz) δ 7.54-7.52 (m, 4H), 7.37-7.31 (m, 6H), 7.15 (td, *J* = 7.8, 1.8 Hz, 1H), 7.07 (dd, *J* = 7.4, 1.8 Hz, 1H), 6.86-6.80 (m, 2H), 4.86 (t, *J* = 3.7 Hz, 1H), 3.75 (s, 3H), 2.68 (t, *J* = 7.6 Hz, 2H), 1.80-1.72 (m, 2H), 1.22-1.17 (m, 2H); ^{13}C NMR ($CDCl_3$, 100 MHz) δ

157.6, 135.3, 134.7, 130.7, 130.1, 129.6, 128.1, 127.1, 120.4, 110.4, 55.3, 33.8, 24.7, 12.2; ^{29}*Si* NMR ($CDCl_3$, 79 MHz) δ -14.00.

(3-(Naphthalen-1-yl)propyl)diphenylsilane (3e)

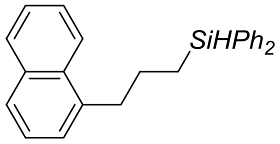

3e

Yield: 144.8 mg, 82%, colorless oil; R_f = 0.29 (hexane : ethyl acetate = 100 : 1); ^{1}H NMR ($CDCl_3$, 400 MHz) δ 7.91-7.89 (m, 1H), 7.82-7.80 (m, 1H), 7.67 (d, *J* = 8.2 Hz, 1H), 7.54-7.51 (m, 4H), 7.43-7.41 (m, 2H), 7.36-7.32 (m, 7H), 7.26-7.24 (m, 1H), 4.89 (t, *J* = 3.8 Hz, 1H), 3.11 (t, *J* = 7.7 Hz, 2H), 1.93 (ddd, *J* = 15.9, 10.3, 6.9 Hz, 2H), 1.31-1.26 (m, 2H); ^{13}*C* NMR ($CDCl_3$, 100 MHz) δ 138.3, 135.2, 134.4, 134.0, 131.9, 129.6, 128.8, 128.1, 126.6, 126.2, 125.7, 125.5, 125.4, 123.9, 36.4, 25.7, 12.4.; ^{29}*Si* NMR ($CDCl_3$, 79 MHz) δ -14.05; HRMS (APPI/LTQ-Orbitrap, *m*/*z*): $[M+H]^+$ calcd for $C_{25}H_{25}Si^+$ = 353.1720, found 353.1721.

(2-Cyclohexylethyl)diphenylsilane (3f) [8]

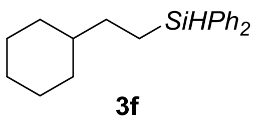

3f

Yield: 140.2 mg, 95%, colorless oil; R_f = 0.46 (hexane); ^{1}H NMR ($CDCl_3$, 400 MHz) δ 7.55-7.53 (m, 4H), 7.39-7.31 (m, 6H), 4.83 (t, *J* = 3.6 Hz, 1H), 1.75-1.60 (m, 5H), 1.37-1.31 (m, 2H), 1.23-1.08 (m, 6H), 0.88-0.78 (m, 2H); ^{13}*C* NMR ($CDCl_3$, 100 MHz) δ 135.3, 134.9, 129.6, 128.1, 40.6, 33.1, 32.0, 26.9, 26.5, 9.3; ^{29}*Si* NMR ($CDCl_3$, 79 MHz) δ -13.06.

(3,3-Dimethylbutyl)diphenylsilane (3g)[12]

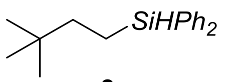

3g

Yield: 124.1 mg, 92%, colorless oil; R_f = 0.51 (hexane); ^{1}H NMR ($CDCl_3$, 400 MHz) δ 7.56-7.54 (m, 4H), 7.39-7.33 (m, 6H), 4.82 (t, *J* = 3.6 Hz, 1H), 1.35-1.30 (m, 2H), 1.11-1.05 (m, 2H), 0.87 (s, 9H); ^{13}*C* NMR ($CDCl_3$, 100 MHz) δ 135.3, 134.8, 129.6, 128.1, 38.5, 31.4, 28.9, 6.6; ^{29}*Si* NMR ($CDCl_3$, 79 MHz) δ -12.07.

(2-(Cyclohex-3-en-1-yl)ethyl)diphenylsilane (3h) [8]

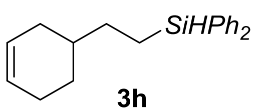

3h

Yield: 130.5 mg, 89%, colorless oil; R_f = 0.40 (hexane); ^{1}H NMR ($CDCl_3$, 400 MHz) δ 7.56-7.54 (m, 4H), 7.37-7.32 (m, 6H), 5.66-5.60 (m, 2H), 4.85 (t, *J* = 3.7 Hz, 1H), 2.14-2.09 (m, 1H), 2.02-1.97 (m, 2H), 1.77-1.72 (m, 1H), 1.65-1.48 (m, 2H), 1.45-1.39 (m, 2H), 1.19-1.14 (m, 3H); ^{13}*C* NMR ($CDCl_3$, 100 MHz) δ 135.3, 134.7, 129.6, 128.1, 127.2, 126.7, 36.5, 31.7, 31.2, 28.6, 25.4, 9.4; ^{29}*Si* NMR ($CDCl_3$, 79 MHz) δ -13.16.

(10-Chlorodecyl)diphenylsilane (3i)

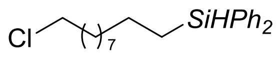

3i

Yield: 153.1 mg, 86%, colorless oil; R_f = 0.34 (hexane); ^{1}H NMR ($CDCl_3$, 400 MHz) δ 7.56-7.53 (m, 4H), 7.37-7.31 (m, 6H), 4.85 (t, *J* = 3.7 Hz, 1H), 3.49 (t, *J* = 6.8 Hz, 2H), 1.73 (p, *J* = 6.9 Hz, 2H), 1.49ff-1.33 (m, 6H), 1.24 (br., 8H), 1.16-1.11 (m. 2H); ^{13}*C* NMR ($CDCl_3$, 100 MHz) δ 135.2, 134.8, 129.6, 128.1, 45.3, 33.2, 32.8, 29.6, 29.5, 29.3, 29.0, 27.0, 24.5, 12.3; ^{29}*Si* NMR ($CDCl_3$, 79 MHz) δ -13.76; HRMS (APPI/LTQ-Orbitrap, *m*/*z*): $[M+H]^+$ calcd for $C_{22}H_{30}ClSi^+$ = 357.1800, found 357.1808.

Diphenyl(12,12,12-trifluorododecyl)silane (3j)

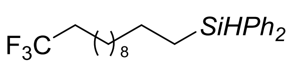

3j

Yield: 140.6 mg, 66%, colorless oil; R_f = 0.40 (hexane); ^{1}H NMR ($CDCl_3$, 400 MHz) δ 7.56-7.54 (m, 4H), 7.38-7.32 (m, 6H), 4.84 (t, *J* = 3.7 Hz, 1H), 2.10-1.98 (m, 2H), 1.57-1.49 (m, 2H), 1.47-1.41 (m, 2H), 1.37-1.31 (m, 4H), 1.29-1.21 (m, 10H)， 1.16-1.11 (m, 2H); *^{13}C* NMR ($CDCl_3$, 100 MHz) δ 135.3, 134.9, 129.6, 128.1, 127.46 (q, *J* = 276.2 Hz), 33.9 (q, *J* = 28.3 Hz), 33.3, 29.7, 29.6, 29.5, 29.3, 28.8, 24.5, 22.0 (q, *J* = 2.8 Hz), 12.3; *^{29}Si* NMR ($CDCl_3$, 79 MHz) δ -13.77; HRMS (APPI/LTQ-Orbitrap, *m/z*): $[M+H]^+$ calcd for $C_{24}H_{32}F_3Si^+$ = 405.2220, found 405.2227.

7-(Diphenylsilyl)heptanenitrile (3k)

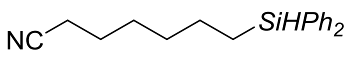

3k

Yield: 88.1 mg, 60%, colorless oil; R_f = 0.21 (hexane : ethyl acetate = 15 : 1); ^{1}H NMR ($CDCl_3$, 400 MHz) δ 7.56-7.53 (m, 4H), 7.40-7.33 (m, 6H), 4.85 (t, *J* = 3.7 Hz, 1H), 2.24 (t, *J* = 7.1 Hz, 2H), 1.61-1.54 (m, 2H), 1.50-1.34 (m, 6H), 1.16-1.12 (m, 2H); *^{13}C* NMR ($CDCl_3$, 100 MHz) δ 135.2, 134.5, 129.7, 128.1, 119.9, 32.2, 28.3, 25.3, 24.2, 17.1, 12.1; *^{29}Si* NMR ($CDCl_3$, 79 MHz) δ -13.84; HRMS (APPI/LTQ-Orbitrap, m/z): $[M+H]^+$ calcd for $C_{19}H_{22}NSi^+$ = 292.1516, found 292.1526.

(3-(Diphenylsilyl)propyl)trimethylsilane (3l)

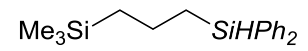

3l

Yield: 141.8 mg, 95%, colorless oil; R_f = 0.44 (hexane); ^{1}H NMR ($CDCl_3$, 400 MHz) δ 7.68-7.65 (m, 4H), 7.50-7.43 (m, 6H), 4.99 (t, *J* = 3.7 Hz, 1H), 1.67-1.59 (m, 2H), 1.35-1.30 (m, 2H), 0.76-0.72 (m, 2H), 0.05 (s, 9H); *^{13}C* NMR ($CDCl_3$, 100 MHz) δ 135.3, 134.9, 129.6, 128.1, 21.0, 19.2, 16.7, -1.4; *^{29}Si* NMR ($CDCl_3$, 79 MHz) δ -0.72, -14.61; HRMS (/LTQ-Orbitrap, m/z): $[M]^+$ calcd for $C_{18}H_{25}Si_2^+$ = 297.1489, found 297.1498.

(2-(Diphenylsilyl)ethyl)dimethyl(phenyl)silane (3m)

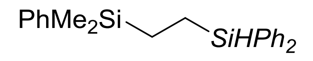

3m

Yield: 158.4 mg, 92%, colorless oil; R_f = 0.34 (hexane); ^{1}H NMR ($CDCl_3$, 400 MHz) δ 7.66-7.64 (m, 4H), 7.62-7.59 (m, 2H), 7.49-7.43 (m, 9H), 4.97 (t, *J* = 3.6 Hz, 1H), 1.23-1.18 (m, 2H), 0.97-0.93 (m, 2H), 0.40 (s, 6H); *^{13}C* NMR ($CDCl_3$, 100 MHz) δ 139.1, 135.3, 134.6, 133.8, 129.7, 129.0, 128.1, 127.9, 8.9, 4.7, -3.4; *^{29}Si* NMR ($CDCl_3$, 79 MHz) δ -1.26, -10.82; HRMS (APPI/LTQ-Orbitrap, m/z): $[M]^+$ calcd for $C_{22}H_{25}Si_2^+$ = 345.1489, found 345.1500.

1-(2-(Diphenylsilyl)ethyl)-1,1,3,3,3-pentamethyldisiloxane (3n) [11]

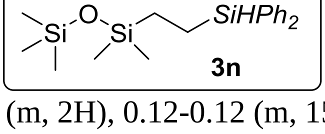

3n

Yield: 151.6 mg, 85%, colorless oil; R_f = 0.60 (hexane : ethyl acetate = 100 : 1); ^{1}H NMR ($CDCl_3$, 400 MHz) δ 7.64-7.62 (m, 4H), 7.45-7.40 (m, 6H), 4.91 (t, *J* = 3.6 Hz, 1H), 1.17-1.12 (m, 2H), 0.66-0.62 (m, 2H), 0.12-0.12 (m, 15H); *^{13}C* NMR ($CDCl_3$, 100 MHz) δ 135.3, 134.8, 129.6, 128.1, 11.4, 4.0, 2.1, -0.1; *^{29}Si* NMR ($CDCl_3$, 79 MHz) δ 7.90, 7.41, -10.66.

Tert-butyl((6-(diphenylsilyl)hexyl)oxy)dimethylsilane (3o)

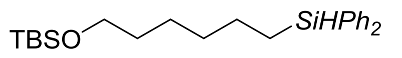

3o

Yield: 144.9 mg, 73%, colorless oil; R_f = 0.31 (hexane : ethyl acetate = 100 : 1); ^{1}H NMR ($CDCl_3$, 400 MHz) δ 7.57-7.55 (m, 4H), 7.40-7.34 (m, 6H), 4.86 (t, *J* = 3.7 Hz, 1H), 3.58 (t, *J* = 6.6 Hz, 2H), 1.52-1.44 (m, 4H), 1.41-1.29 (m,

4H), 1.18-1.13 (m, 2H), 0.90 (s, 9H), 0.05 (s, 6H); ^{13}C NMR ($CDCl_3$, 100 MHz) δ 135.3, 134.8, 129.6, 128.1, 63.4, 33.1, 32.9, 26.1, 25.5, 24.5, 18.5, 12.3, -5.1; ^{29}Si NMR ($CDCl_3$, 79 MHz) δ 18.30, -13.78; HRMS (APPI/LTQ-Orbitrap, m/z): $[M+H]^+$ calcd for $C_{24}H_{37}OSi^+$ = 397.2377, found 397.2393.

(3-(1,4-Dioxaspiro[4.5]decan-6-yl)propyl)diphenylsilane (3p)

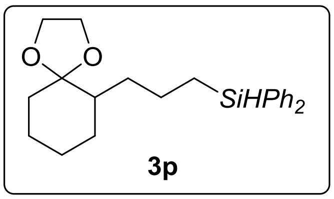

3p

Yield: 136.0 mg, 74%, light yellow oil; R_f = 0.24 (hexane : ethyl acetate = 15 : 1); ^1H NMR ($CDCl_3$, 400 MHz) δ 7.56-7.53 (m, 4H), 7.40-7.32 (m, 6H), 4.84 (t, J = 3.7 Hz, 1H), 3.93-3.81 (m, 4H), 1.74-1.69 (m, 2H), 1.65-1.50 (m, 5H), 1.43-1.28 (m, 3H), 1.22-1.10 (m, 5H); ^{13}C NMR ($CDCl_3$, 100 MHz) δ 135.3, 135.3, 134.9, 134.8, 129.6, 129.6, 128.1, 111.0, 64.9, 64.8, 44.5, 34.9, 31.9, 29.2, 24.7, 24.0, 22.5, 12.7; ^{29}Si NMR ($CDCl_3$, 79 MHz) δ -13.76; HRMS (APPI/LTQ-Orbitrap, m/z): $[M+H]^+$ calcd for $C_{23}H_{30}O_2Si^+$ = 366.2010, found 366.2018.

6-(Diphenylsilyl)hexyl acetate (3q)

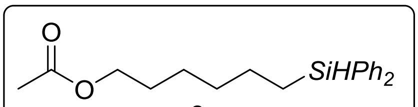

3q

Yield: 98.3 mg, 63%, colorless oil; R_f = 0.31 (hexane : ethyl acetate = 15 : 1); ^1H NMR ($CDCl_3$, 400 MHz) δ 7.56-7.53 (m, 4H), 7.38-7.33 (m, 6H), 4.85 (t, J = 3.7 Hz, 1H), 4.02 (t, J = 6.7 Hz, 2H), 2.02 (s, 3H), 1.60-1.53 (m, 2H), 1.49-1.30 (m, 6H), 1.16-1.12 (m, 2H); ^{13}C NMR ($CDCl_3$, 100 MHz) δ 171.3, 135.2, 134.7, 129.6, 128.1, 64.7, 32.8, 28.6, 25.6, 24.4, 21.1, 12.2; ^{29}Si NMR ($CDCl_3$, 79 MHz) δ -13.84; HRMS (APPI/LTQ-Orbitrap, m/z): $[M+H]^+$ calcd for $C_{20}H_{25}O_2Si^+$ = 325.1618, found 325.1632.

Methyl 11-(diphenylsilyl)undecanoate (3r)

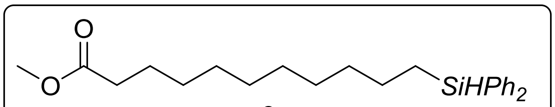

3r

Yield: 177.6 mg, 93%, colorless oil; R_f = 0.39 (hexane : ethyl acetate = 15 : 1); ^1H NMR ($CDCl_3$, 400 MHz) δ 7.56-7.53 (m, 4H), 7.39-731 (m, 6H), 4.85 (t, J = 3.7 Hz, 1H), 3.64 (s, 3H), 2.28 (t, J = 7.5 Hz, 2H), 1.64-1.56 (m, 2H), 1.49-1.41 (m, 2H), 1.38-1.33 (m, 2H), 1.28-1.23 (m, 10H), 1.16-1.11 (m, 2H); ^{13}C NMR ($CDCl_3$, 100 MHz) δ 174.4, 135.2, 134.8, 129.5, 128.0, 51.5, 34.2, 33.2, 29.5, 29.5, 29.3, 29.3, 29.2, 25.1, 24.5, 12.2; ^{29}Si NMR ($CDCl_3$, 79 MHz) δ -13.73; HRMS (APPI/LTQ-Orbitrap, m/z): $[M+H]^+$ calcd for $C_{24}H_{33}O_2Si^+$ = 381.2244, found 381.2256.

(4-(Oxiran-2-yl)butyl)diphenylsilane (3s)

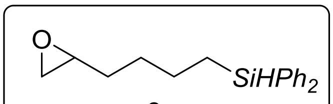

3s

Yield: 106.3 mg, 75%, colorless oil; R_f = 0.33 (hexane : ethyl acetate = 30 : 1); ^1H NMR ($CDCl_3$, 400 MHz) δ 7.56-7.53 (m, 4H), 7.40-7.32 (m, 6H), 4.86 (t, J = 3.7 Hz, 1H), 2.84 (t, J = 3.8 Hz, 1H), 2.69 (t, J = 4.5 Hz, 1H), 2.40 (dd, J = 5.0, 2.8 Hz, 1H), 1.54-1.49 (m, 6H), 1.18-1.15 (m, 2H); ^{13}C NMR ($CDCl_3$, 100 MHz) δ 135.2, 134.5, 129.7, 128.1, 52.3, 47.2, 32.2, 29.5, 24.4, 12.2; ^{29}Si NMR ($CDCl_3$, 79 MHz) δ -13.90; HRMS (APPI/LTQ-Orbitrap, m/z): $[M+H]^+$ calcd for $C_{18}H_{21}OSi^+$ = 281.1356, found 281.1365.

(2-(7-Oxabicyclo[4.1.0]heptan-3-yl)ethyl)diphenylsilane (3t)[8]

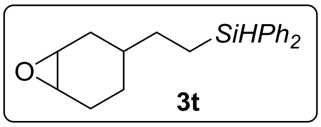

Yield: 117.4 mg, 76%, colorless oil; R_f = 0.17 (hexane : ethyl acetate = 30 : 1); ^{1}H NMR ($CDCl_3$, 400 MHz) δ 7.54-7.52 (m, 4H), 7.40-7.32 (m, 6H), 4.83 (t, *J* = 3.3 Hz, 1H), 3.13-3.08 (m, 2H), 2.20-1.91 (m, 2H), 1.83-1.60 (m, 1H), 1.53-1.27 (m, 4H), 1.25-0.82 (m, 4H); ^{13}C NMR ($CDCl_3$, 100 MHz) δ 135.2, 134.5, 129.7, 128.1, 53.3, 52.8, 52.0, 51.9, 35.3, 32.4, 31.6, 31.3, 30.8, 30.4, 26.8, 25.4, 24.0, 23.6, 9.3, 9.1; ^{29}Si NMR ($CDCl_3$, 79 MHz) δ -13.24, -13.32. (Because of diastereoisomers)

(3-Phenoxypropyl)diphenylsilane (3u) [9]

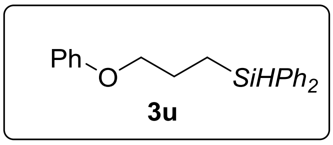

Yield: 86.1 mg, 54%, colorless oil; R_f = 0.48 (hexane : ethyl acetate = 30 : 1); ^{1}H NMR ($CDCl_3$, 400 MHz) δ 7.57-7.55 (m, 4H), 7.38-7.32 (m, 6H), 7.26-7.22 (m, 2H), 6.92-6.83 (m, 3H), 4.92 (t, *J* = 3.6 Hz, 1H), 3.92 (t, *J* = 6.4 Hz, 2H), 1.96-1.89 (m, 2H), 1.31-1.26 (m, 2H); ^{13}C NMR ($CDCl_3$, 100 MHz) δ 159.1, 135.3, 134.2, 129.8, 129.5, 128.2, 120.6, 114.6, 69.9, 24.4, 8.6; ^{29}Si NMR ($CDCl_3$, 79 MHz) δ -13.72.

3-(Diphenylsilyl)-N,N-dimethylpropan-1-amine (3v) [11]

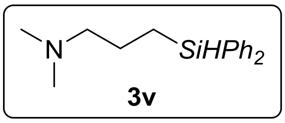

Yield: 103.0 mg, 77%, colorless oil; R_f = 0.63 (hexane: ethyl acetate : triethylamine = 4 : 2 : 1); ^{1}H NMR ($CDCl_3$, 400 MHz) δ 7.56-7.54 (m, 4H), 7.39-7.31 (m, 6H), 4.87 (t, *J* = 3.7 Hz, 1H), 2.27 (t, *J* = 7.4 Hz, 2H), 2.15 (s, 6H), 1.65-1.57 (m, 2H), 1.17-1.12 (m, 2H); ^{13}C NMR ($CDCl_3$, 100 MHz) δ 135.2, 134.5, 129.6, 128.1, 62.8, 45.5, 22.7, 9.9; ^{29}Si NMR ($CDCl_3$, 79 MHz) δ -13.64.

1-(3-(diphenylsilyl)propyl)-1H-indole (3w)

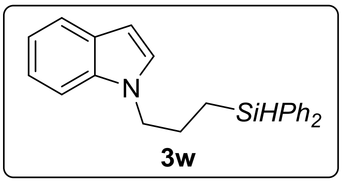

Yield: 120.2 mg, 70%, yellow oil; R_f = 0.47 (hexane : ethyl acetate = 15 : 1); ^{1}H NMR ($CDCl_3$, 400 MHz) δ 7.61 (d, *J* = 7.8 Hz, 1H), 7.48 (d, *J* = 7.8 Hz, 4H). 7.38-7.29 (m, 6H), 7.22 (d, *J* = 8.2 Hz, 1H), 7.15 (t, *J* = 7.5 Hz, 1H), 7.07 (t, *J* = 7.4 Hz, 1H), 7.01 (d, *J* = 3.1 Hz, 1H), 6.45 (d, *J* = 3.1 Hz, 1H), 4.85 (t, *J* = 3.7 Hz, 1H), 4.07 (t, *J* = 7.1 Hz, 2H), 2.00-1.92 (m, 2H), 1.12-1.07 (m, 2H); ^{13}C NMR ($CDCl_3$, 100 MHz) δ 136.1, 135.2, 133.8, 129.9, 128.7, 128.2, 127.9, 121.5, 121.1, 119.3, 109.5, 101.1, 49.0, 25.4, 9.5; ^{29}Si NMR ($CDCl_3$, 79 MHz) δ -14.09; HRMS (APPI/LTQ-Orbitrap, m/z): $[M+H]^+$ calcd for $C_{23}H_{24}NSi^+$ = 342.1673, found 342.1681.

Phenethyldiphenylsilane (3x) and diphenyl(1-phenylethyl)silane (3x') mixture [10]

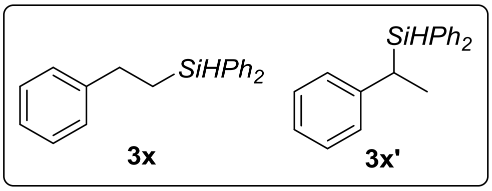

Yield: 122.2 mg, 85%, colorless oil; R_f = 0.19 (hexane); ^{1}H NMR ($CDCl_3$, 400 MHz) δ 7.71-7.63 (m, 3H), 7.53-7.43 (m, 6H), 7.38 (d, *J* = 7.4 Hz, 2H), 7.31-7.18 (m, 3H), 7.14-7.12 (m, 1H), 5.03 (t, *J* = 3.7 Hz, 0.53H), 4.97 (d, *J* = 3.4 Hz, 0.47H), 2.96-2.93 (m, 0.45H), 2.91-2.87 (m, 1.17H), 1.66-1.61 (m, 1.14H), 1.59 (d, *J* = 7.5 Hz, 1.44H); ^{13}C NMR ($CDCl_3$, 100 MHz) δ 144.5, 144.5, 136.0, 135.8, 135.7, 135.3, 134.2, 133.2, 129.8, 129.8, 129.7, 128.5, 128.3, 128.2, 128.0, 128.0, 127.9, 127.8, 125.9, 125.0, 30.6, 27.1, 16.6, 14.4^{29}Si NMR ($CDCl_3$, 79 MHz) δ -8.32, -14.09.

Diphenyl(2-phenylpropyl)silane (3y) [8]

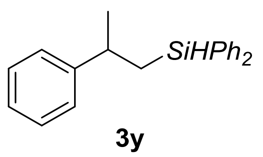

3y

Yield: 79.6 mg, 53%, colorless oil; R_f = 0.15 (hexane); ^{1}H NMR ($CDCl_3$, 400 MHz) δ 7.53-7.48 (m, 4H), 7.38-7.29 (m, 6H), 7.26-7.22 (m, 2H), 7.17-7.15 (m, 3H), 4.78 (t, *J* = 3.9 Hz, 1H), 2.99-2.90 (m, 1H), 1.61-1.49 (m, 2H), 1.31 (d, *J* = 6.9 Hz, 3H); ^{13}*C* NMR ($CDCl_3$, 100 MHz) δ 149.2, 135.3, 135.2, 134.8, 134.6, 129.7, 129.6, 128.5, 128.1, 128.1, 126.7, 126.1, 36.3, 25.3, 22.7; ^{29}*Si* NMR ($CDCl_3$, 79 MHz) δ -15.67.

(2,2-diphenylethyl)diphenylsilane (3z) [10]

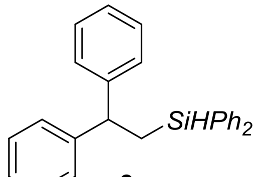

3z

Yield: 107.5 mg, 59%, colorless oil; R_f = 0.48 (petroleum : ethyl acetate = 20 : 1); ^{1}H NMR ($CDCl_3$, 400 MHz) δ 7.47-7.45 (m, 4H), 7.38-7.28 (m, 6H), 7.22-7.19 (m, 8H), 7.15-7.11 (m, 2H), 4.57 (t, *J* = 3.8 Hz, 1H), 4.11 (t, *J* = 8.0 Hz, 1H), 1.99 (dd, *J* = 8.0, 3.9 Hz, 2H), ; ^{13}*C* NMR ($CDCl_3$, 100 MHz) δ 146.3, 135.2, 134.3, 129.7, 128.5, 128.1, 127.8, 126.3, 47.2, 20.4; ^{29}*Si* NMR ($CDCl_3$, 79 MHz) δ -15.38.

Octyldiphenylsilane (3b) [8]

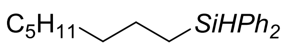

3b'

Yield: 90.1 mg, 61%, colorless oil; R_f = 0.54 (hexane); ^{1}H NMR ($CDCl_3$, 400 MHz) δ 7.56-7.54 (m, 4H), 7.39-7.31 (m, 6H), 4.85 (t, *J* = 3.7 Hz, 1H), 1.48-1.42 (m, 2H), 1.37-1.33 (m, 2H), 1.27-1.23 (m, 8H), 1.16-1.11 (m, 2H), 0.86 (t, *J* = 6.9 Hz, 3H); ^{13}*C* NMR ($CDCl_3$, 100 MHz) δ 135.3, 134.9, 129.6, 128.1, 33.3, 32.0, 29.4, 29.3, 24.6, 22.8, 14.3, 12.3; ^{29}*Si* NMR ($CDCl_3$, 79 MHz) δ -13.76.

((1S,4R)-Bicyclo[2.2.1]heptan-2-yl)diphenylsilane (3ac) [8]

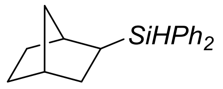

3ac

Yield: 115.7 mg, 83%, colorless oil; R_f= 0.39 (hexane); ^{1}H NMR ($CDCl_3$, 400 MHz) δ 7.58-7.54 (m, 4H), 7.37-7.30 (m, 6H), 4.69 (d, *J* = 5.3 Hz, 1H), 2.28 (d, *J* = 21.8 Hz, 2H), 1.60-1.49 (m, 4H), 1.34-1.22 (m, 4H), 1.13-1.10 (m, 1H); ^{13}*C* NMR ($CDCl_3$, 100 MHz) δ 135.5, 135.4, 135.1, 134.4, 129.4, 127.9, 127.9, 38.5, 37.6, 37.3, 33.9, 33.6, 29.2, 25.7; ^{29}*Si* NMR ($CDCl_3$, 79 MHz) δ -10.94.

Cyclopentyldiphenylsilane (3ad)[12]

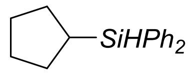

3ad

Yield: 84.3 mg, 67%, colorless oil; R_f = 0.49 (hexane); ^{1}H NMR ($CDCl_3$, 400 MHz) δ 7.58-7.56 (m, 4H), 7.39-7.31 (m, 6H), 4.77 (d, *J* = 3.3 Hz, 1H), 1.94-1.87 (m, 2H), 1.60-1.44 (m, 7H); ^{13}*C* NMR ($CDCl_3$, 100 MHz) δ 135.6, 134.8, 129.5, 128.0, 29.3, 27.1, 22.8; ^{29}*Si* NMR ($CDCl_3$, 79 MHz) δ -9.83.

Octyldiphenylsilanol (4)

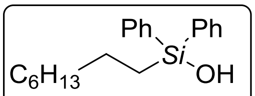

4

Yield: 113.9 mg, 73%, light yellow oil; R*f* = 0.39 (hexane : ethyl acetate = 15 : 1); ^{1}H NMR ($CDCl_3$, 400 MHz) δ 7.57-7.54 (m, 4H), 7.39-7.28 (m, 6H), 2.82 (brs, 1H), 1.57-1.49 (m, 2H), 1.44-1.33 (m, 10H), 1.23-1.19 (m, 2H), 0.97 (t, J = 6.8 Hz, 3H); ^{13}*C* NMR ($CDCl_3$, 100 MHz) δ 136.5, 134.3, 129.9, 128.0, 33.6, 32.0, 29.4, 29.3, 23.1, 22.8, 15.2, 14.2; ^{29}*Si* NMR ($CDCl_3$, 79 MHz) δ -3.43; HRMS (APPI/LTQ-Orbitrap, m/z): $[M+H]^+$ calcd for $C_{20}H_{29}OSi^+$ = 313.1982, found 313.1991.

(3-(2-Methoxyphenyl)propyl)diphenylsilane-d (*D*-3d)

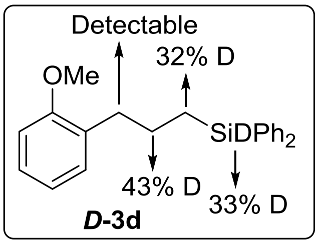

Yield: 51.9 mg (0.25 mmol scale), 62%; 1H NMR (CDCl3, 400 MHz) δ 7.54-7.52 (m, 4H), 7.38-7.30 (m, 6H), 7.16-7.12 (m, 1H), 7.06 (d, J = 7.3 Hz, 1H), 6.86-6.79 (m, 2H), 4.87-4.86 (m, 0.67 H), 3.74 (s, 3H), 2.70-2.66 (m, 1.97 H), 1.78-1.72 (m, 1.14H), 1.19-1.17 (m, 1.34H); 13C NMR (CDCl3, 100 MHz) δ 157.6, 135.3, 134.7, 130.7, 130.1, 129.6, 128.1, 127.1, 120.4, 110.4, 55.3, 33.8-33.6 (m, 1C), 24.7-24.3 (m, 1C), 12.2-12.0 (m, 1C). 2H NMR (CDCl3, 61.4 MHz, TMS-d12 = 0 ppm) 4.75 (brs, 0.25D), 2.48 (brs, 0.08D), 1.55 (brs, 1D), 1.02 (brs, 0.68D).

8. Reference

1. Tyrra, W.; Naumann, D.; Quadt, S.; Buslei, S.; Yagupolskii, Y. L.; Kremlev, M. M.; *J. Fluorine Chem.* **2007**, *128*, 813.
2. Schleicher, K. D.; Jamison, T. F. *Org. Lett.* **2007**, *9*, 875.
3. Schank, K.; Beljan, P. *J. Prakt. Chem.* **1995**, *337*, 558.
4. Xu, S.; Huang, X.; Hong, X.; Xu, B. *Org. Lett.* **2012**, *14*, 4614.
5. Safronov, A. V.; Shlyakhtina, N. I.; Everett, T. A.; VanGordon, M. R.; Sevryugina, Y. V.; Jalisatgi, S. S.; Hawthorne, M. F. *Inorg. Chem.* **2014**, *53*, 10045.
6. Zhao, S. L.; Wang, Y.; Dong, J. C.; He, C. T.; Yin, H. J.; An, P. F.; Zhao, K.; Zhang, X. F.; Gao, C.; Zhang, L. J.; Lv, J. W.; Wang, J. X.; Zhang, J. Q.; Khattak, A. M.; Khan, N. A.; Wei, Z. X.; Zhang, J.; Liu, S. Q.; Zhao, H. J.; Tang, Z. Y., *Nat. Energy* **2016,** *1*, 1.
7. Takahashi, T.; Hasegawa, M.; Suzuki, N.; Saburi, M.; Rousset, C. J.; Fanwick, P. E.; Negishi, E. *J. Am. Chem. Soc*. **1991**, *113*, 8564.
8. Buslov, I.; Becouse, J.; Mazza, S.; Montandon-Clerc, M.; Hu, X. *Angew. Chem. Int. Ed.* **2015**, *54*, 14523.
9. Vijaykumar, G.; Pariyar, A.; Ahmed, J.; Shaw, B. K. ; Adhikari, D.; Mandal, S. K. *Chem. Sci.* **2018**, *9*, 2817.
10. Kuznetsov, A.; Gevorgyan, V. *Org. Lett*. **2012**. *14*, 914.
11. Toya, Y.; Hayasaka, K.; Nakazawa, H. *Organometallics* **2017**, *36*, 1727.
12. The regents of the University of California; Tilley, T. D.; Yang, J. WO/2013/120057, **2013**, A1.

9. ^{1}H, ^{13}C and ^{29}Si spectra

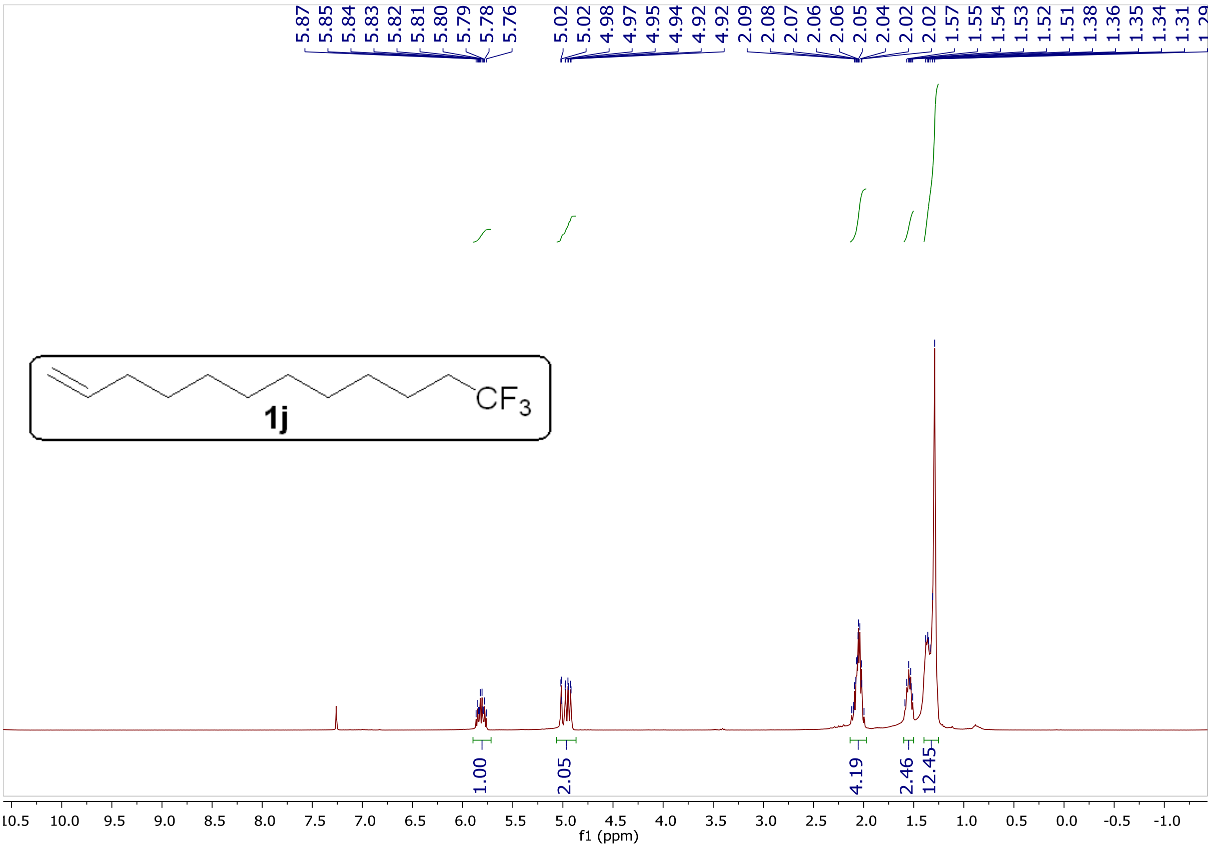

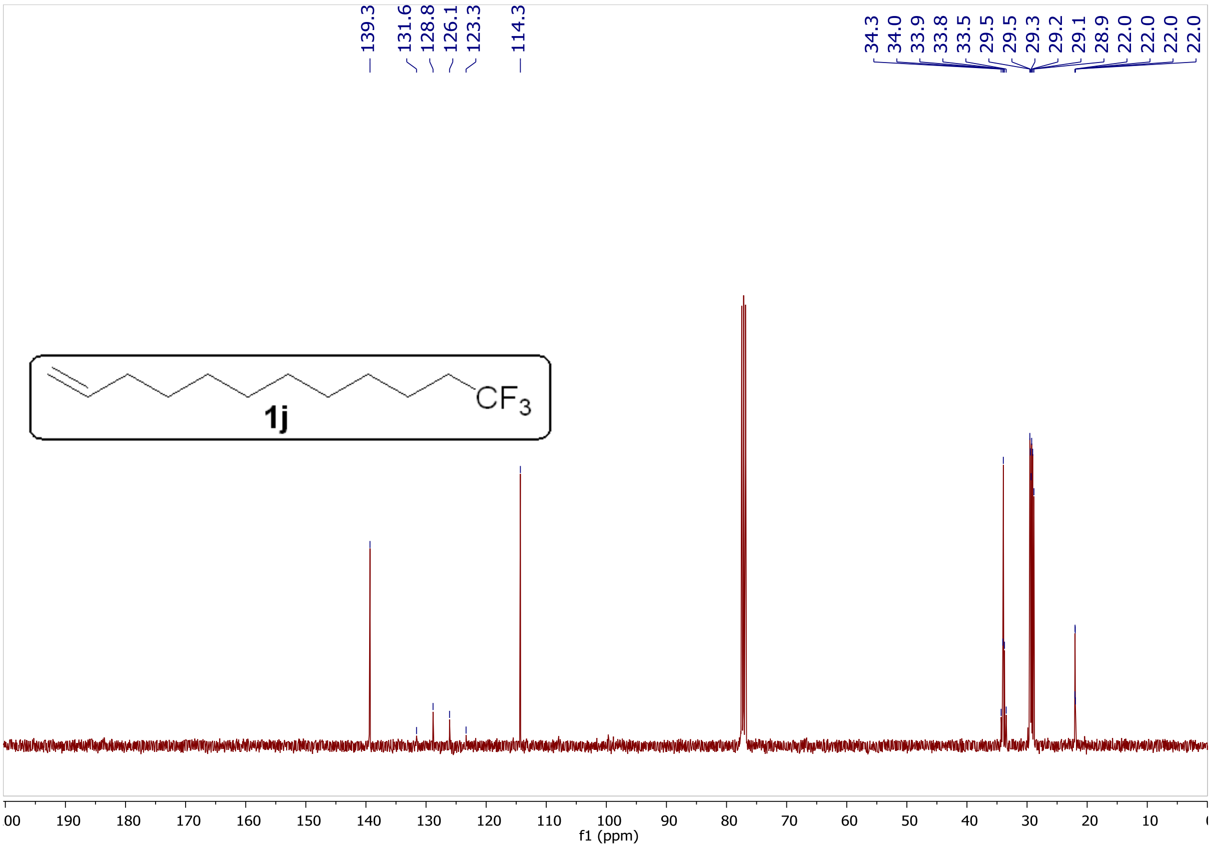

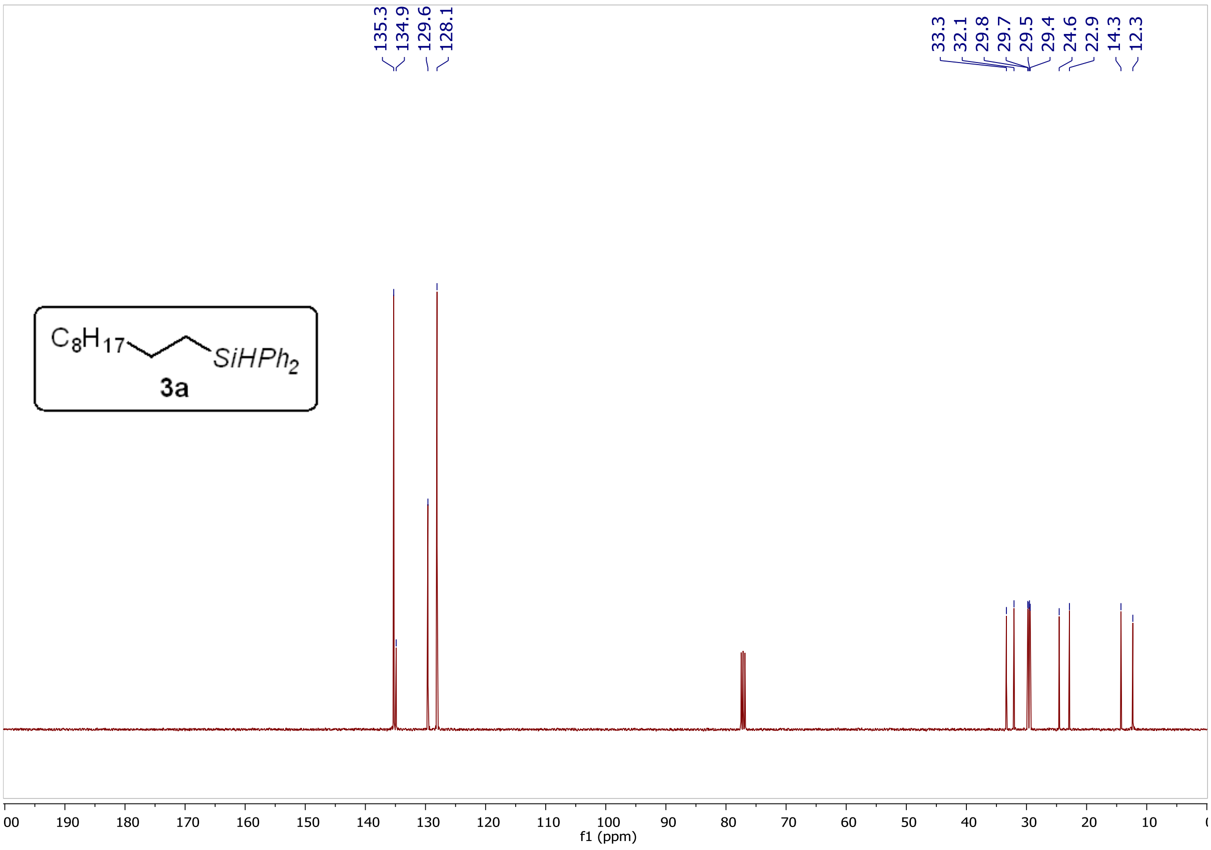
135.3
134.9
129.6
128.1
33.3
32.1
29.8
29.7
29.5
29.4
24.6
22.9
14.3
12.3
C8H17 SiHPh2
3a
f1 (ppm)

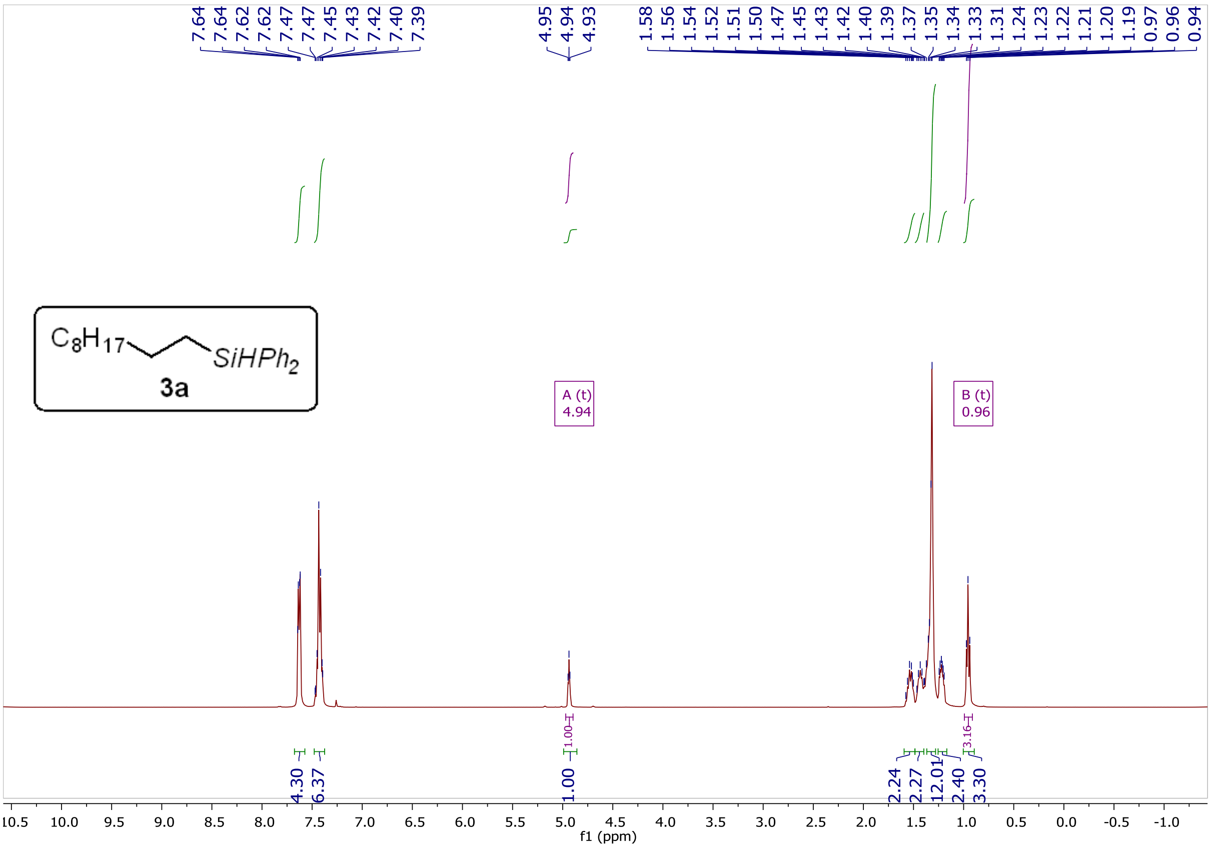
7.64
7.64
7.62
7.62
7.47
7.47
7.45
7.43
7.42
7.40
7.39
4.95
4.94
4.93
1.58
1.56
1.54
1.52
1.51
1.50
1.47
1.45
1.43
1.42
1.40
1.39
1.37
1.35
1.34
1.33
1.31
1.24
1.23
1.22
1.21
1.20
1.19
0.97
0.96
0.94
C8H17 SiHPh2
3a
A (t)
4.94
B (t)
0.96
4.30
6.37
1.00
2.24
2.27
12.01
2.40
3.30
f1 (ppm)

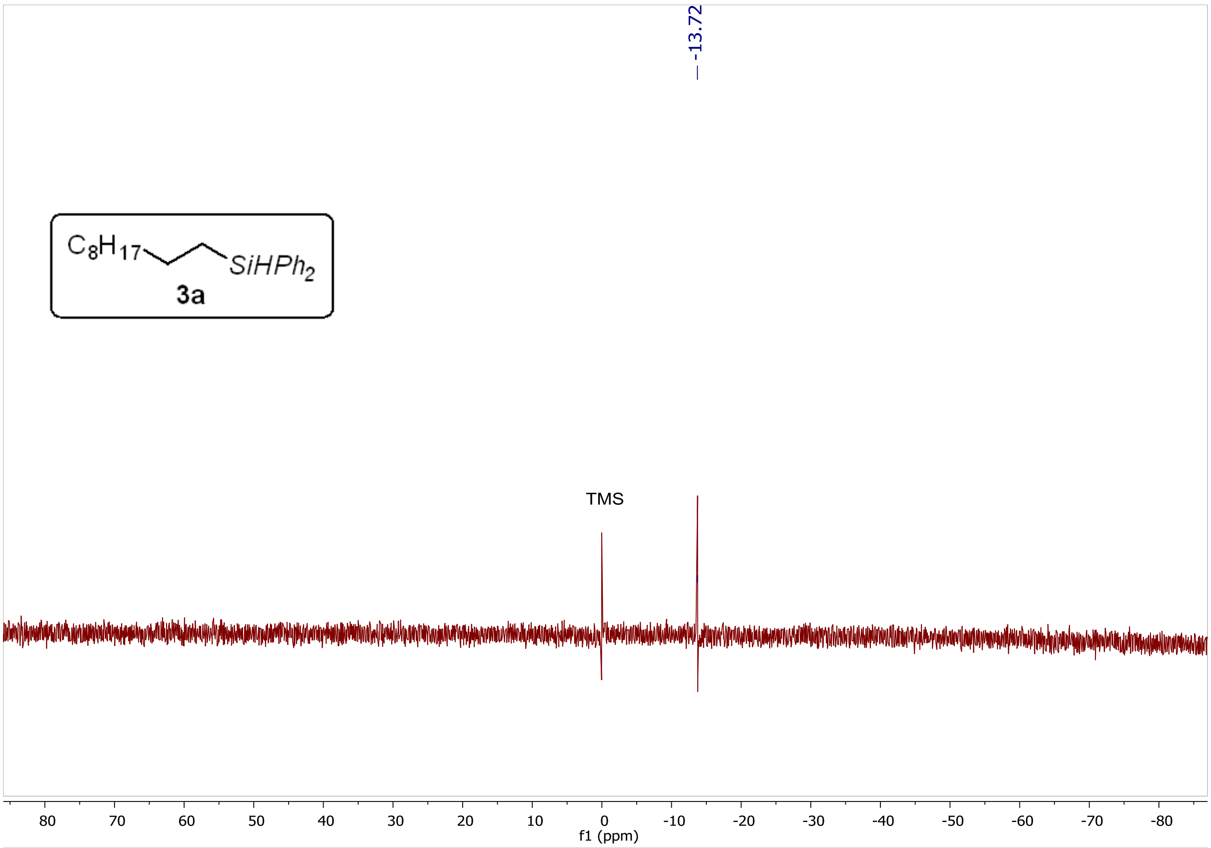

-13.72
C8H17 SiHPh2
3a
TMS
80 70 60 50 40 30 20 10 0 -10 -20 -30 -40 -50 -60 -70 -80
f1 (ppm)

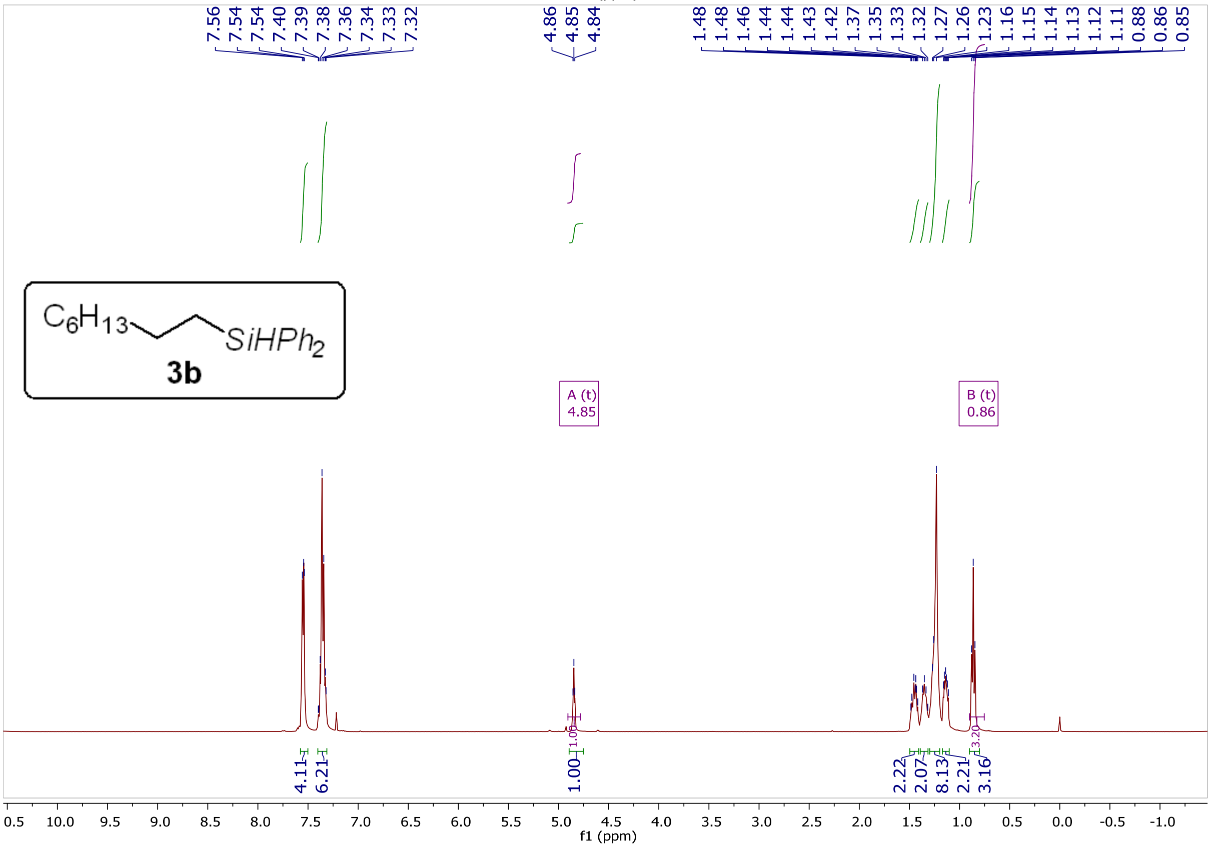

7.56 7.54 7.54 7.40 7.39 7.38 7.36 7.34 7.33 7.32
4.86 4.85 4.84
1.48 1.48 1.46 1.44 1.44 1.43 1.42 1.37 1.35 1.33 1.32 1.27 1.26 1.23 1.16 1.15 1.14 1.13 1.12 1.11 0.88 0.86 0.85
C6H13 SiHPh2
3b
A (t) 4.85
B (t) 0.86
4.11 6.21 1.00 2.22 2.07 8.13 2.21 3.16
f1 (ppm)

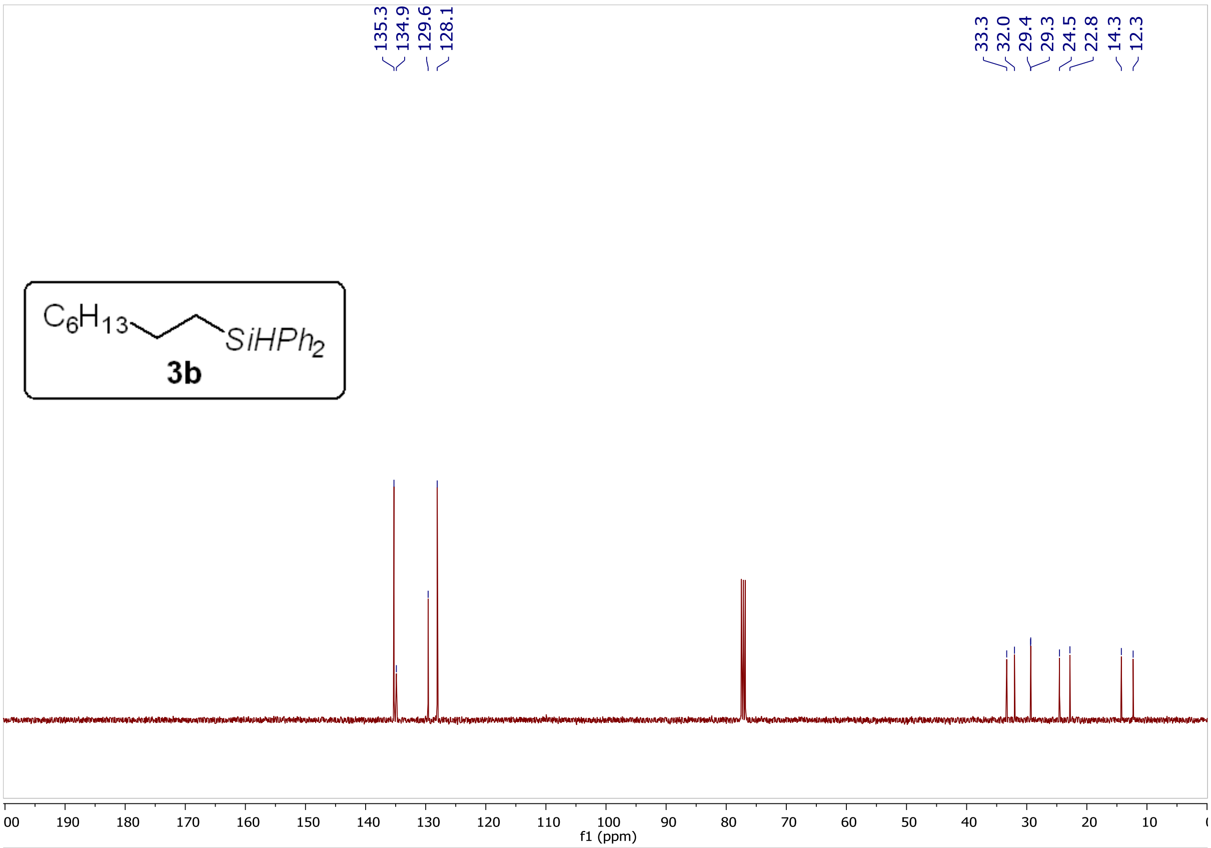
135.3
134.9
129.6
128.1
33.3
32.0
29.4
29.3
24.5
22.8
14.3
12.3
C6H13
SiHPh2
3b
f1 (ppm)

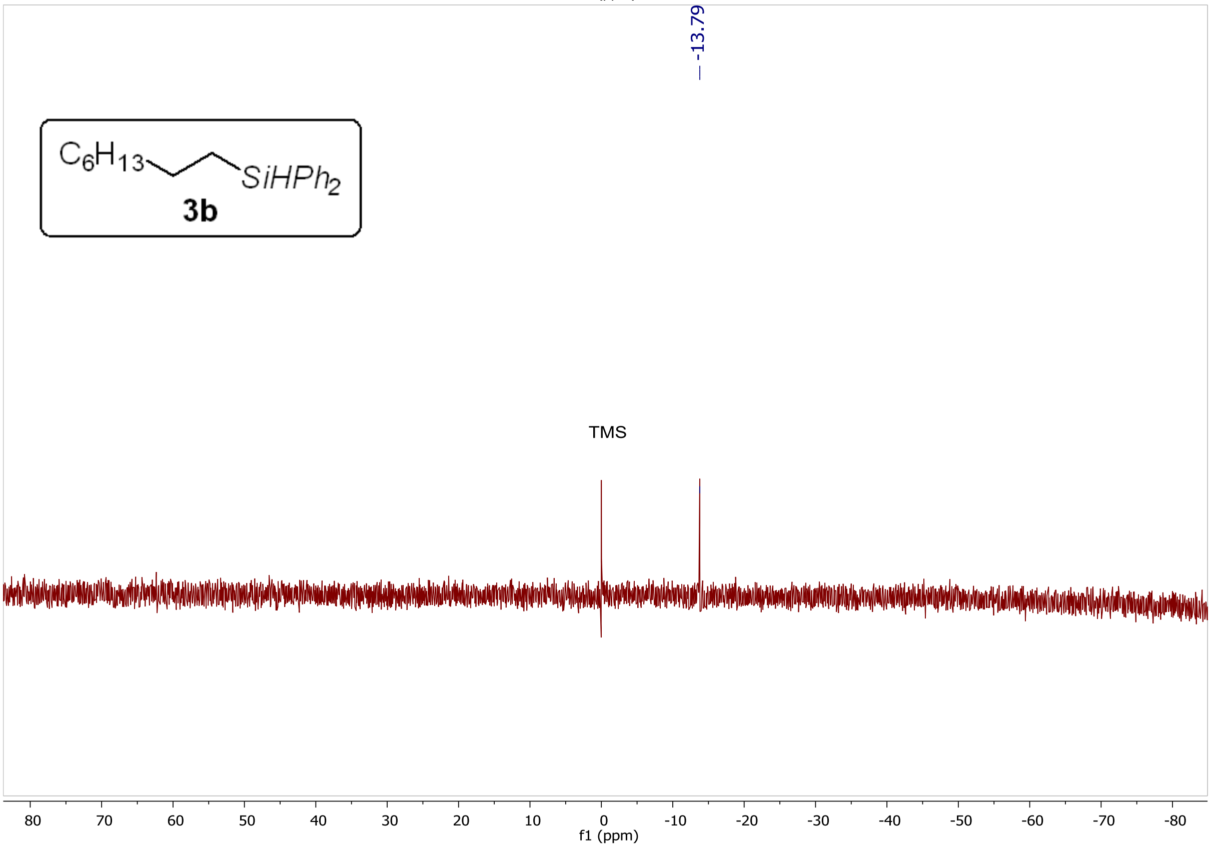
-13.79
C6H13
SiHPh2
3b
TMS
f1 (ppm)

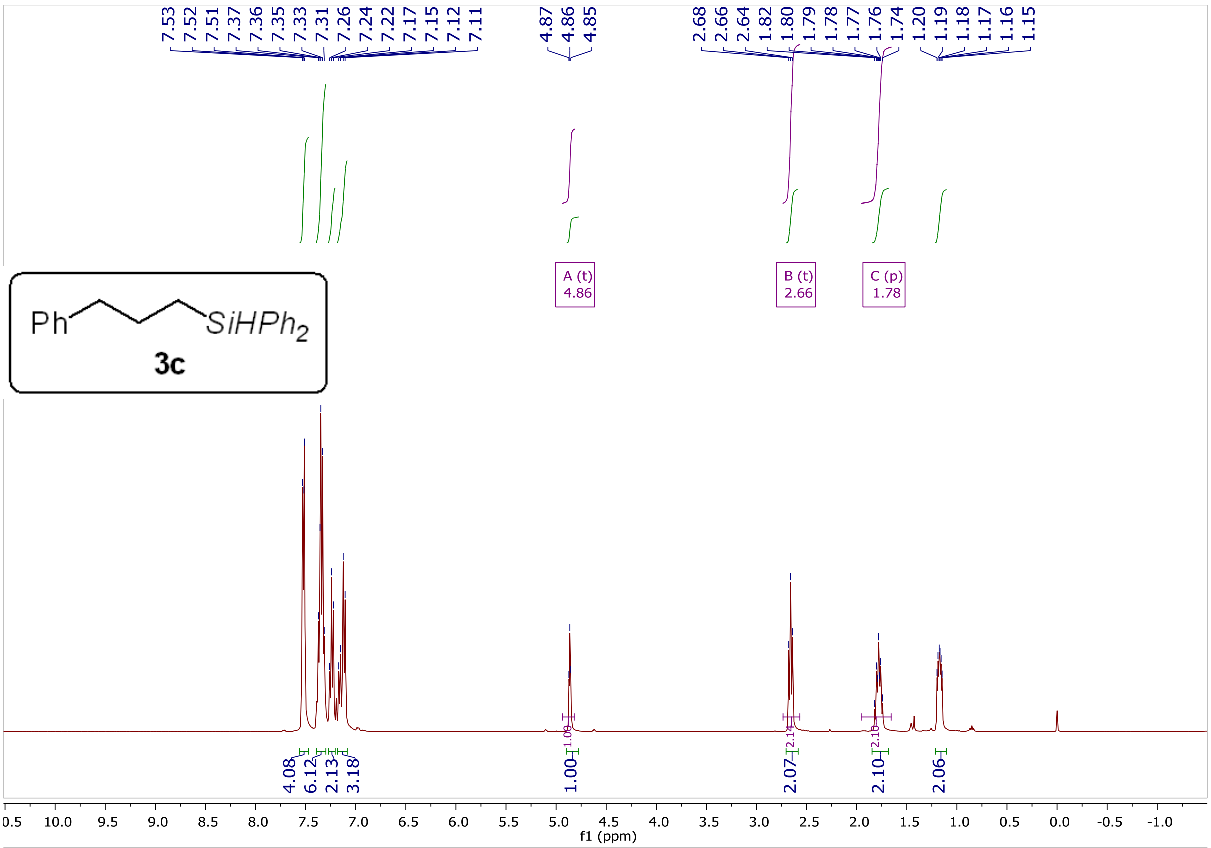
7.53 7.52 7.51 7.37 7.36 7.35 7.33 7.31 7.26 7.24 7.22 7.17 7.15 7.12 7.11
4.87 4.86 4.85
2.68 2.66 2.64 1.82 1.80 1.79 1.78 1.77 1.76 1.74 1.20 1.19 1.18 1.17 1.16 1.15
A (t) 4.86
B (t) 2.66
C (p) 1.78
Ph
SiHPh2
3c
4.08 6.12 2.13 3.18
1.00
2.07
2.10
2.06
f1 (ppm)

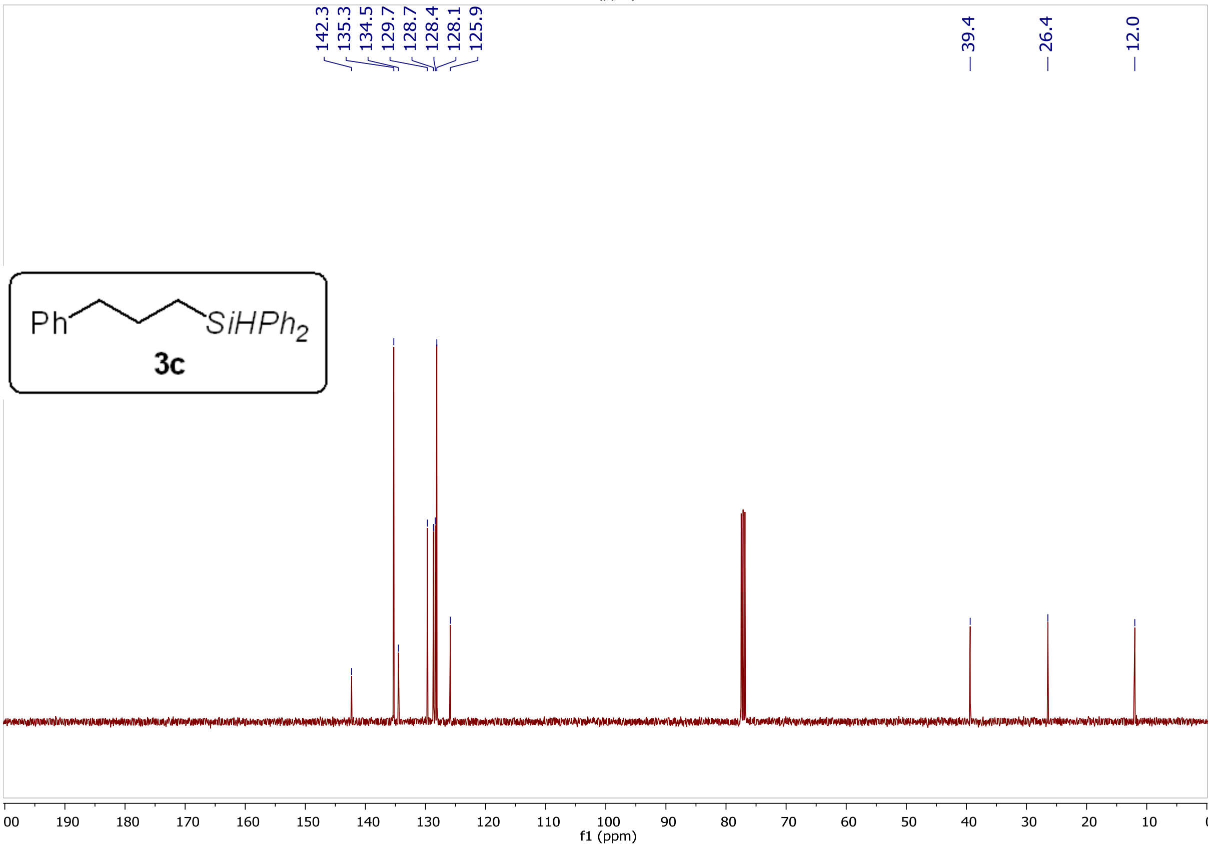
142.3 135.3 134.5 129.7 128.7 128.4 128.1 125.9
39.4
26.4
12.0
Ph
SiHPh2
3c
f1 (ppm)

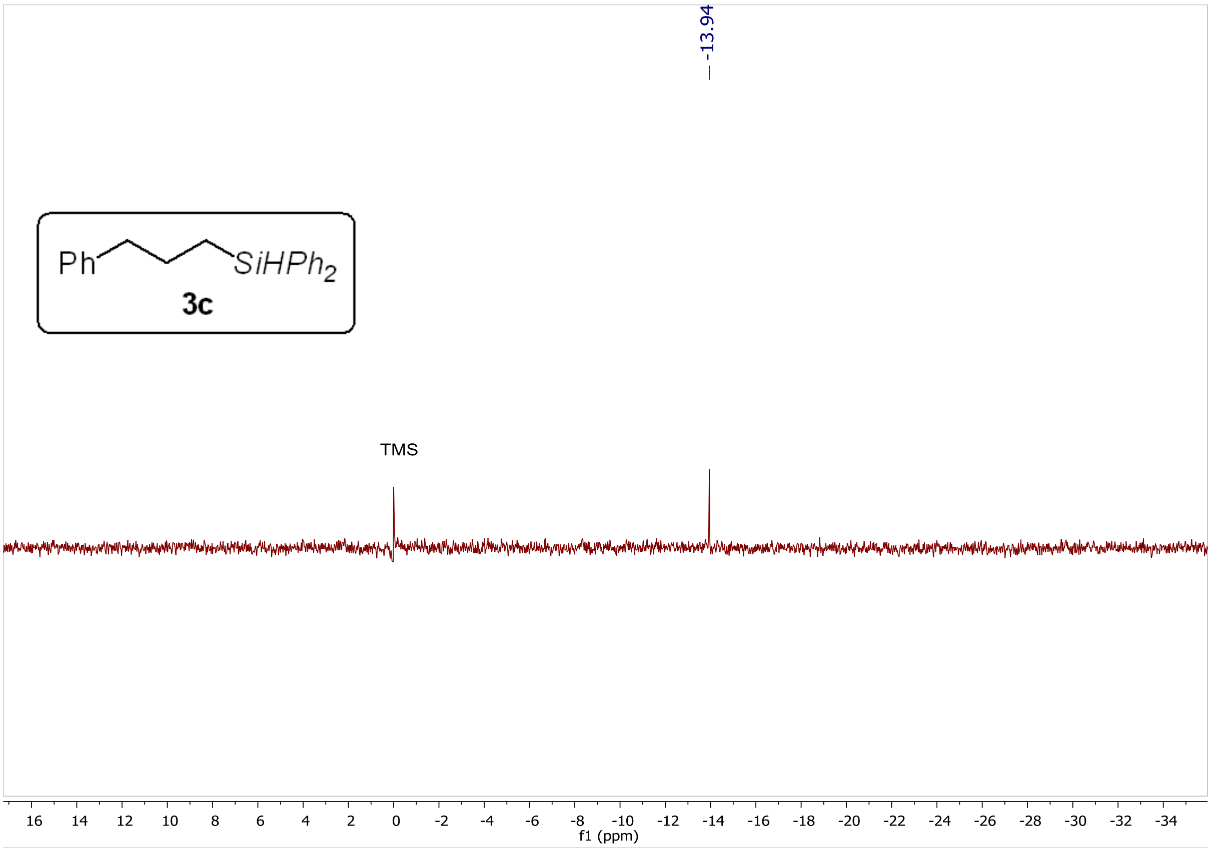

-13.94
Ph SiHPh2
3c
TMS
f1 (ppm)

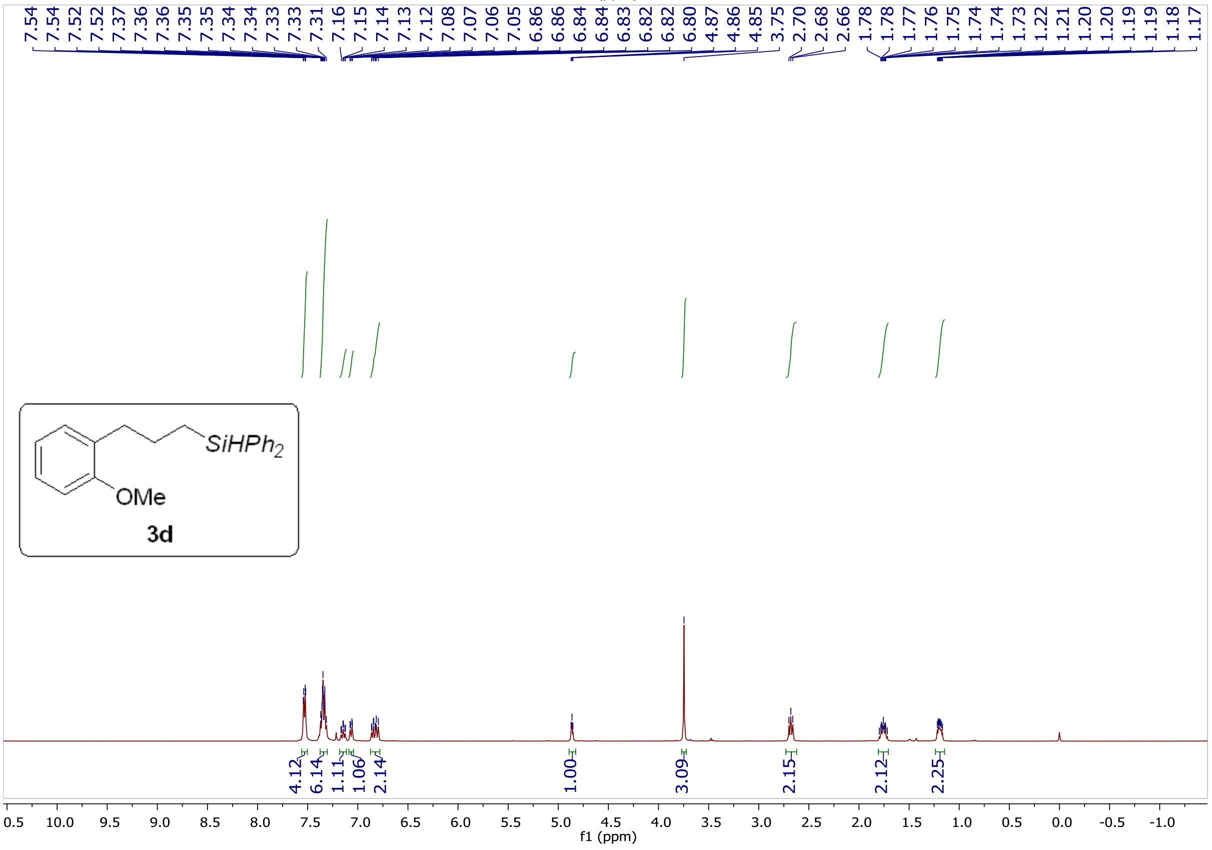

SiHPh2
OMe
3d
4.12
6.14
1.11
1.06
2.14
1.00
3.09
2.15
2.12
2.25
f1 (ppm)

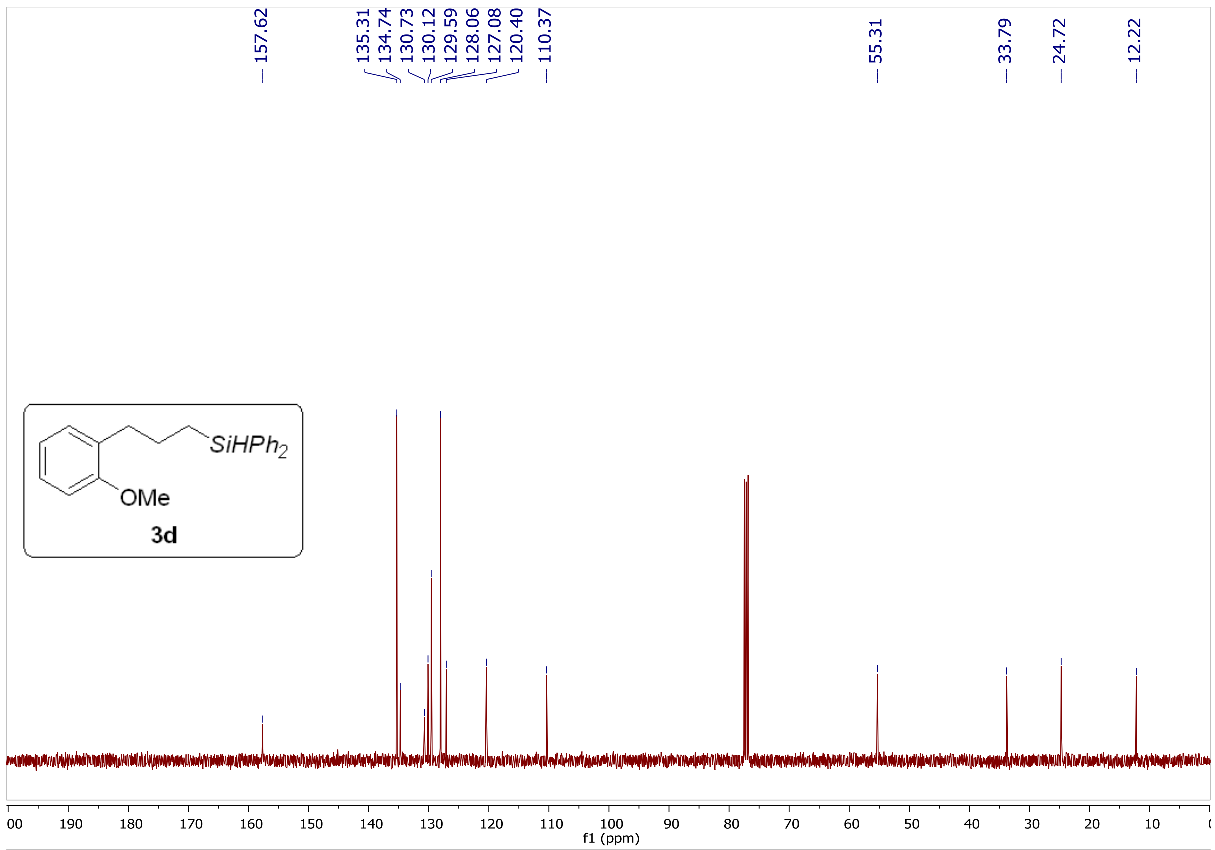
157.62
135.31
134.74
130.73
130.12
129.59
128.06
127.08
120.40
110.37
55.31
33.79
24.72
12.22
SiHPh2
OMe
3d
f1 (ppm)

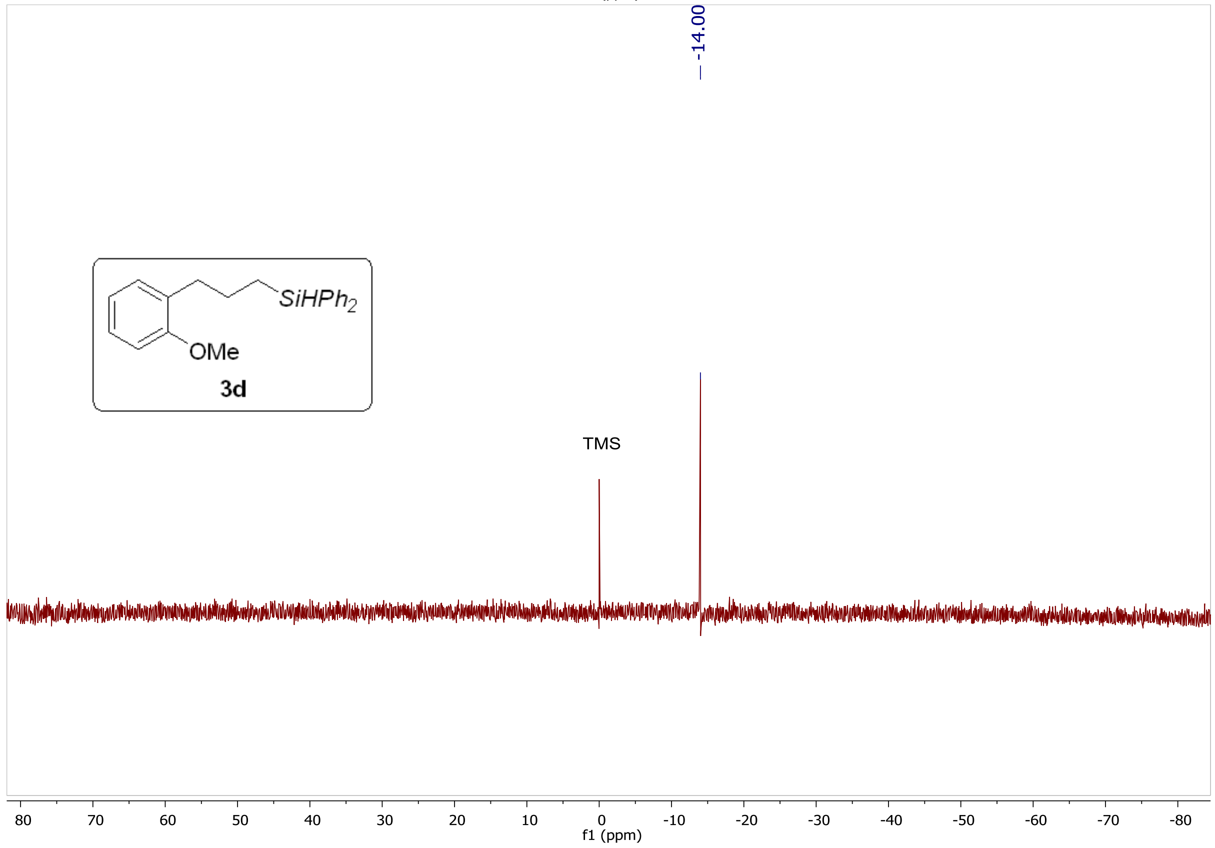
-14.00
SiHPh2
OMe
3d
TMS
f1 (ppm)

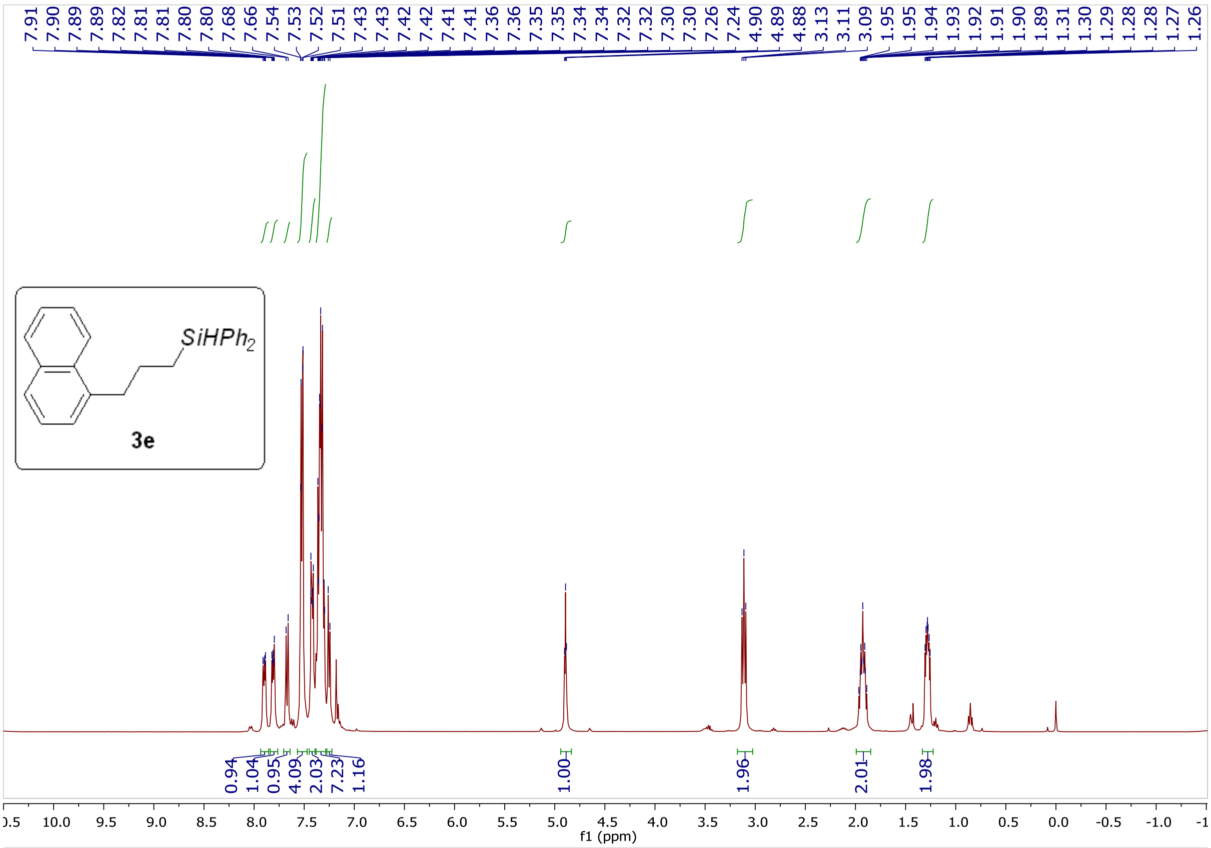
7.91 7.90 7.89 7.89 7.82 7.81 7.81 7.80 7.80 7.68 7.66 7.54 7.53 7.52 7.51 7.43 7.43 7.42 7.42 7.41 7.41 7.36 7.36 7.35 7.35 7.34 7.34 7.32 7.32 7.30 7.30 7.26 7.24 4.90 4.89 4.88 3.13 3.11 3.09 1.95 1.95 1.94 1.93 1.92 1.91 1.90 1.89 1.31 1.30 1.29 1.28 1.28 1.27 1.26
SiHPh2
3e
0.94 1.04 0.95 4.09 2.03 7.23 1.16
1.00
1.96
2.01
1.98
f1 (ppm)

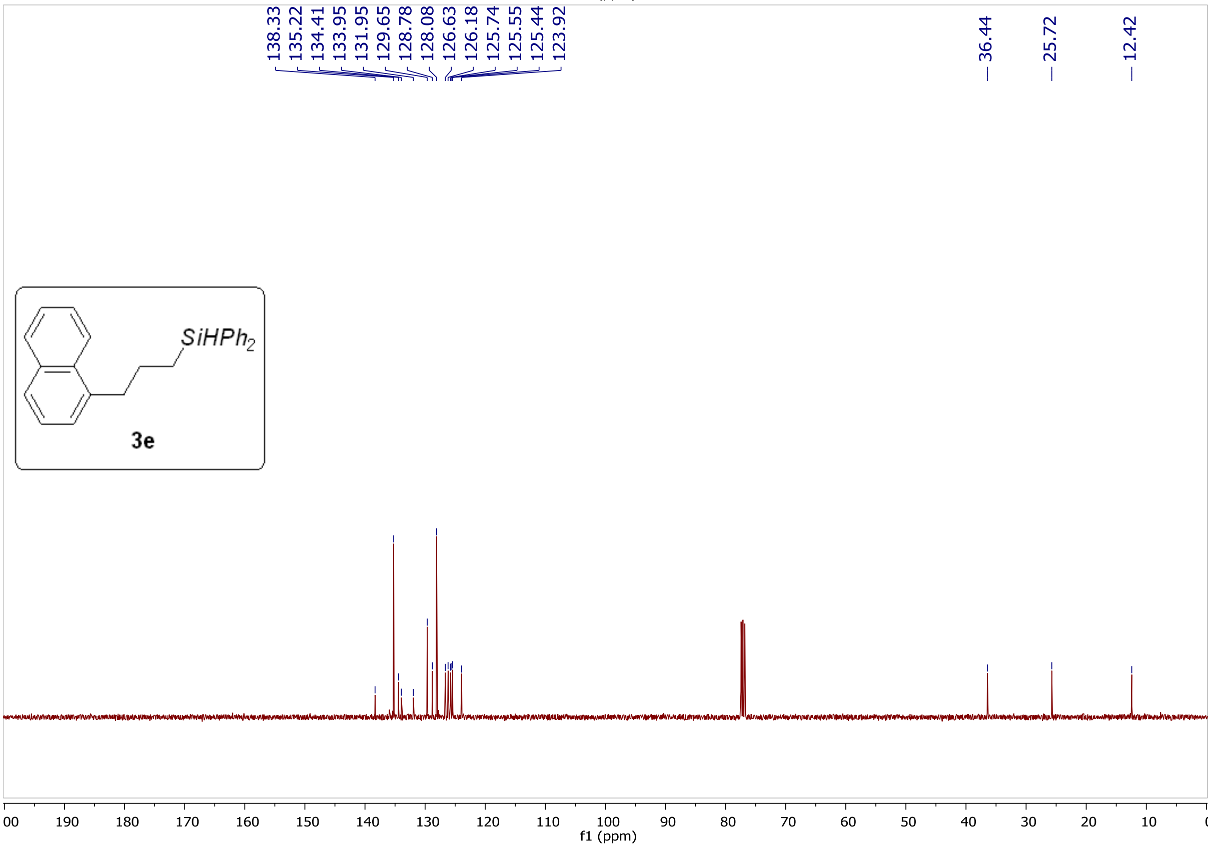
138.33 135.22 134.41 133.95 131.95 129.65 128.78 128.08 126.63 126.18 125.74 125.55 125.44 123.92
36.44
25.72
12.42
SiHPh2
3e
f1 (ppm)

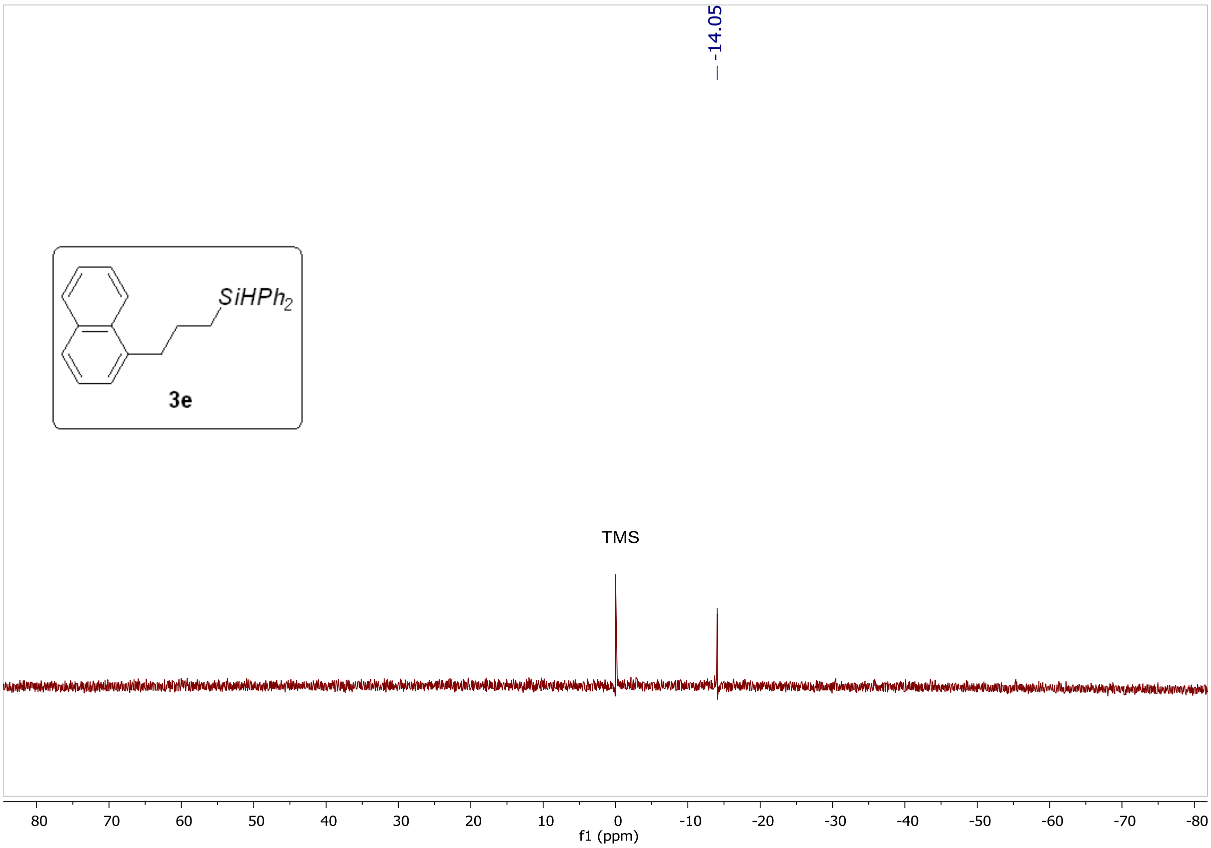
-14.05
SiHPh2
3e
TMS
80 70 60 50 40 30 20 10 0 -10 -20 -30 -40 -50 -60 -70 -80
f1 (ppm)

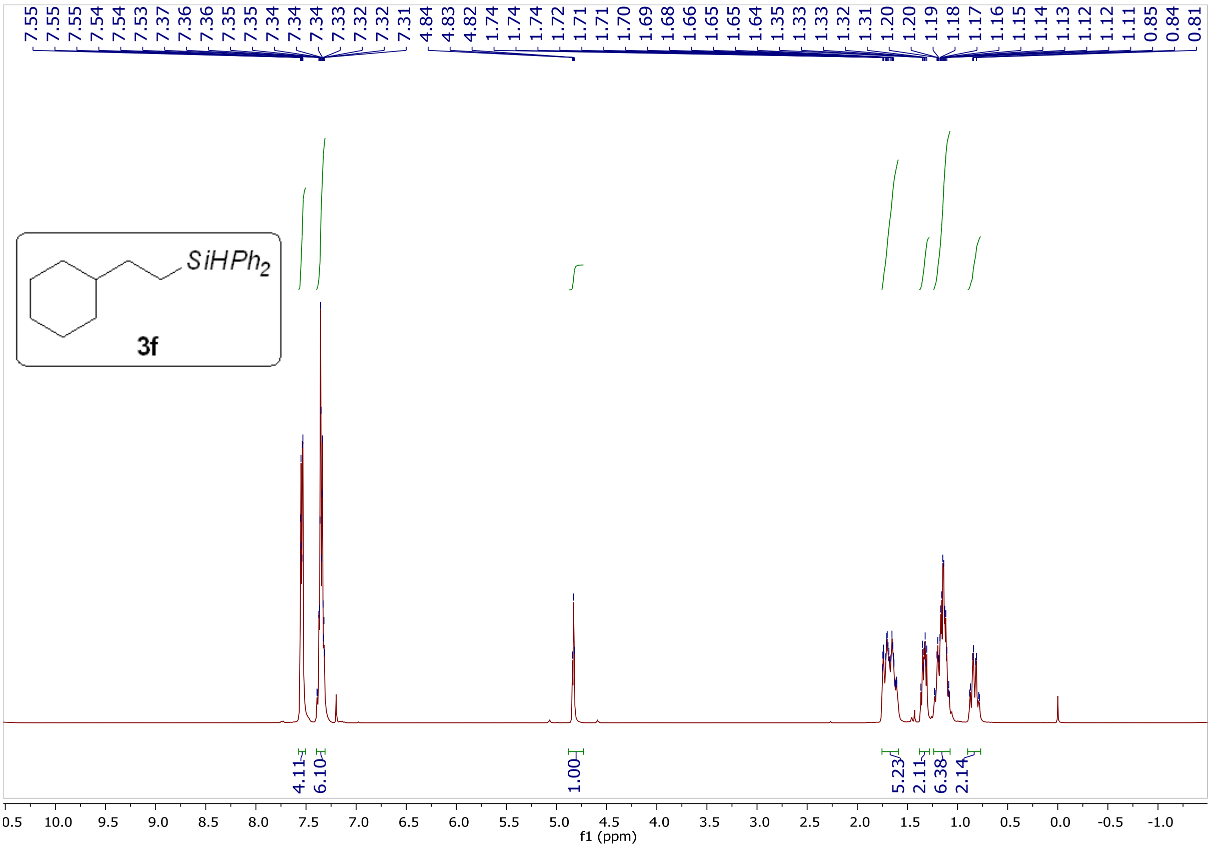
7.55 7.55 7.55 7.54 7.54 7.53 7.37 7.36 7.36 7.35 7.35 7.34 7.34 7.34 7.33 7.32 7.32 7.31 4.84 4.83 4.82 1.74 1.74 1.74 1.72 1.71 1.71 1.70 1.69 1.68 1.66 1.65 1.65 1.64 1.35 1.33 1.33 1.32 1.31 1.20 1.20 1.19 1.18 1.17 1.16 1.15 1.14 1.13 1.12 1.12 1.11 0.85 0.84 0.81
SiHPh2
3f
4.11 6.10 1.00 5.23 2.11 6.38 2.14
0.5 10.0 9.5 9.0 8.5 8.0 7.5 7.0 6.5 6.0 5.5 5.0 4.5 4.0 3.5 3.0 2.5 2.0 1.5 1.0 0.5 0.0 -0.5 -1.0
f1 (ppm)

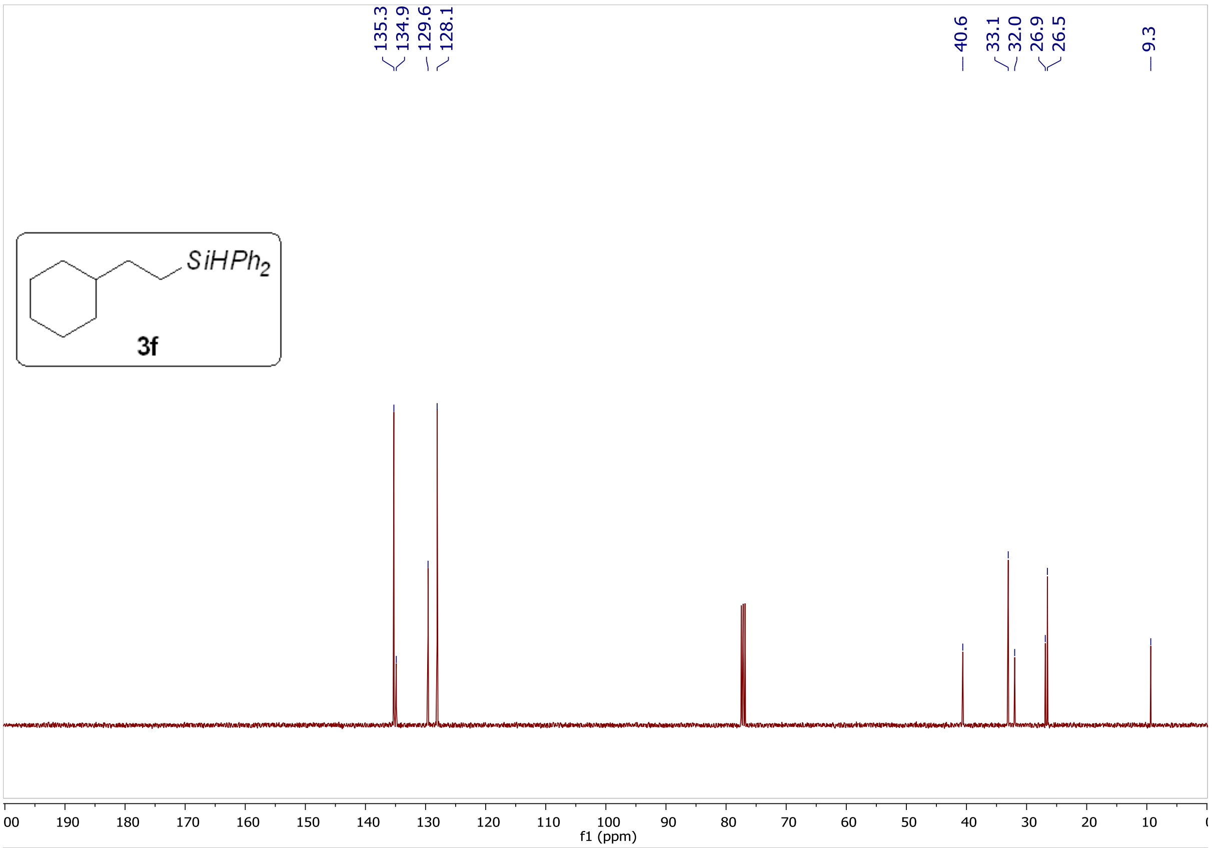
135.3
134.9
129.6
128.1
40.6
33.1
32.0
26.9
26.5
9.3
SiHPh2
3f
f1 (ppm)

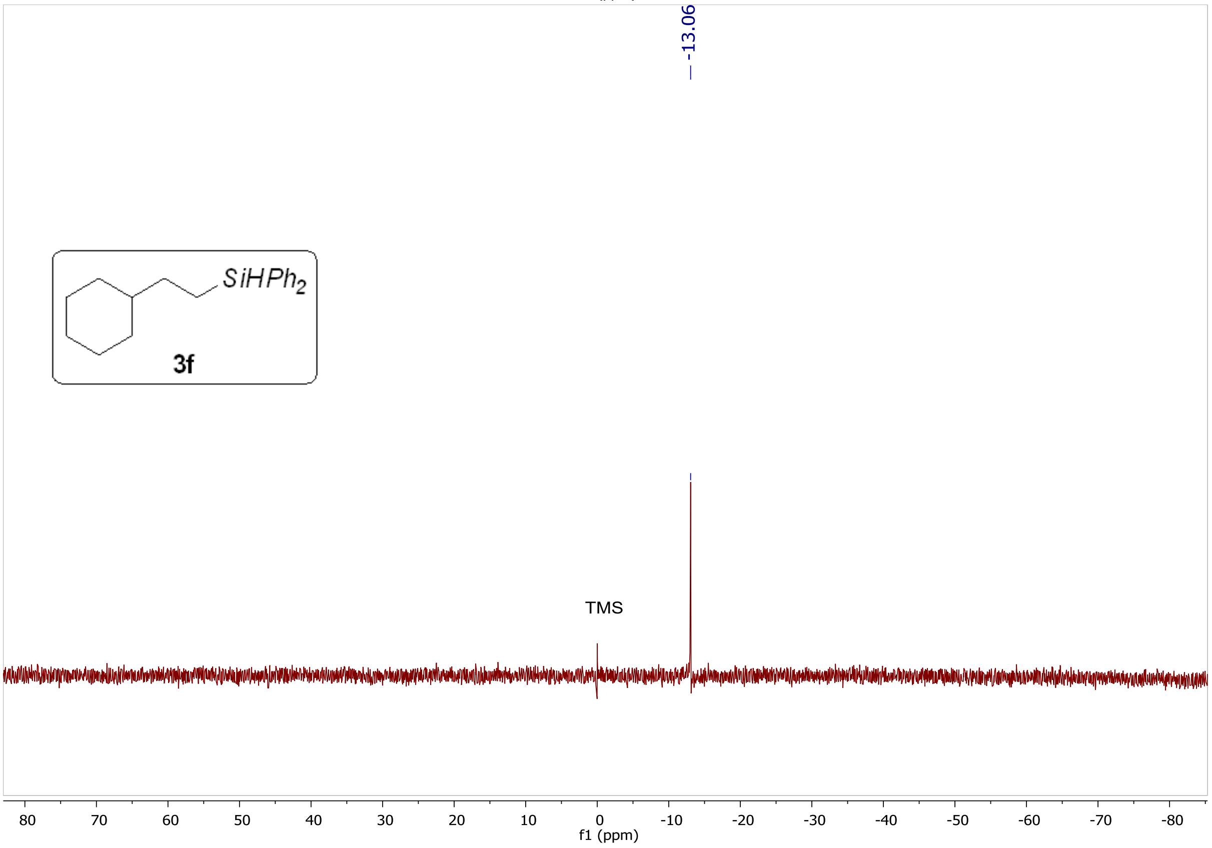
-13.06
SiHPh2
3f
TMS
f1 (ppm)

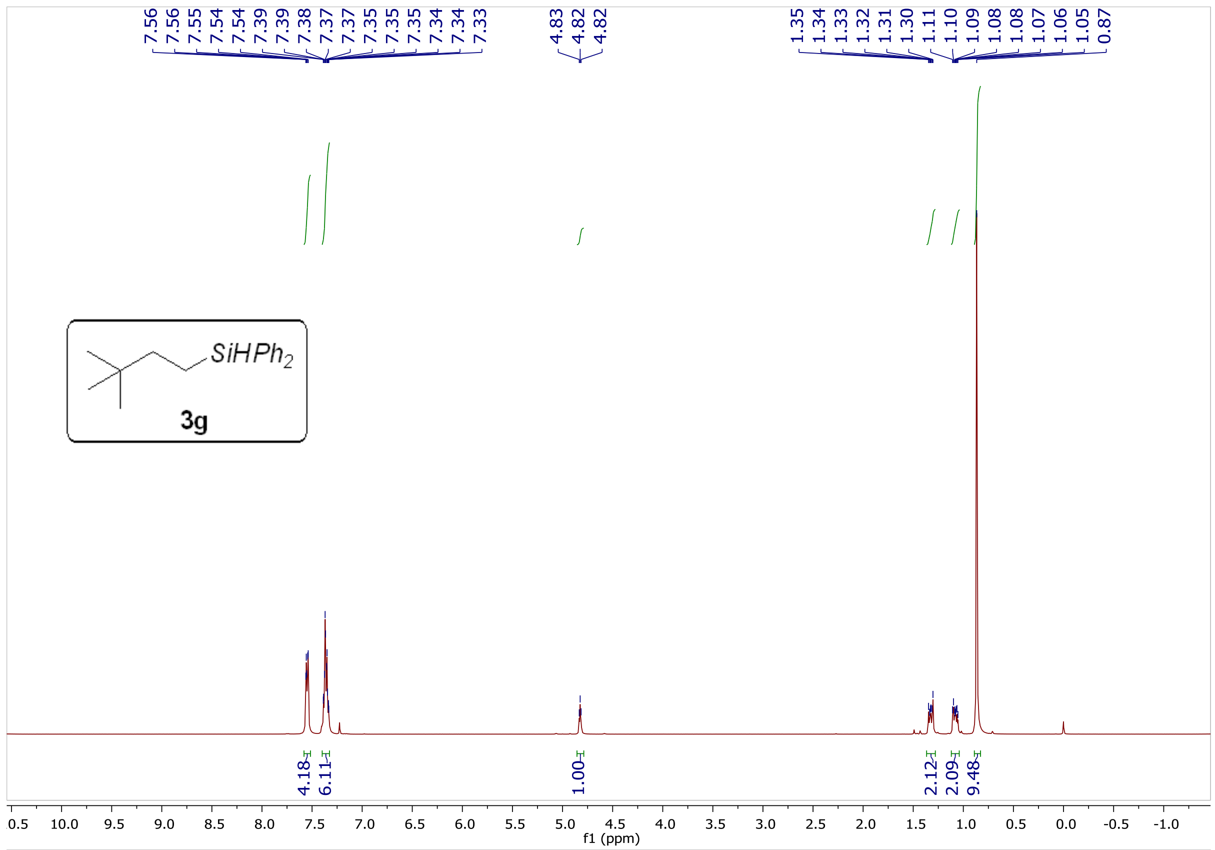
7.56 7.56 7.55 7.54 7.54 7.39 7.39 7.38 7.37 7.37 7.35 7.35 7.35 7.34 7.34 7.33
4.83 4.82 4.82
1.35 1.34 1.33 1.32 1.31 1.30 1.11 1.10 1.09 1.08 1.08 1.07 1.06 1.05 0.87
SiHPh2
3g
4.18 6.11 1.00 2.12 2.09 9.48
f1 (ppm)

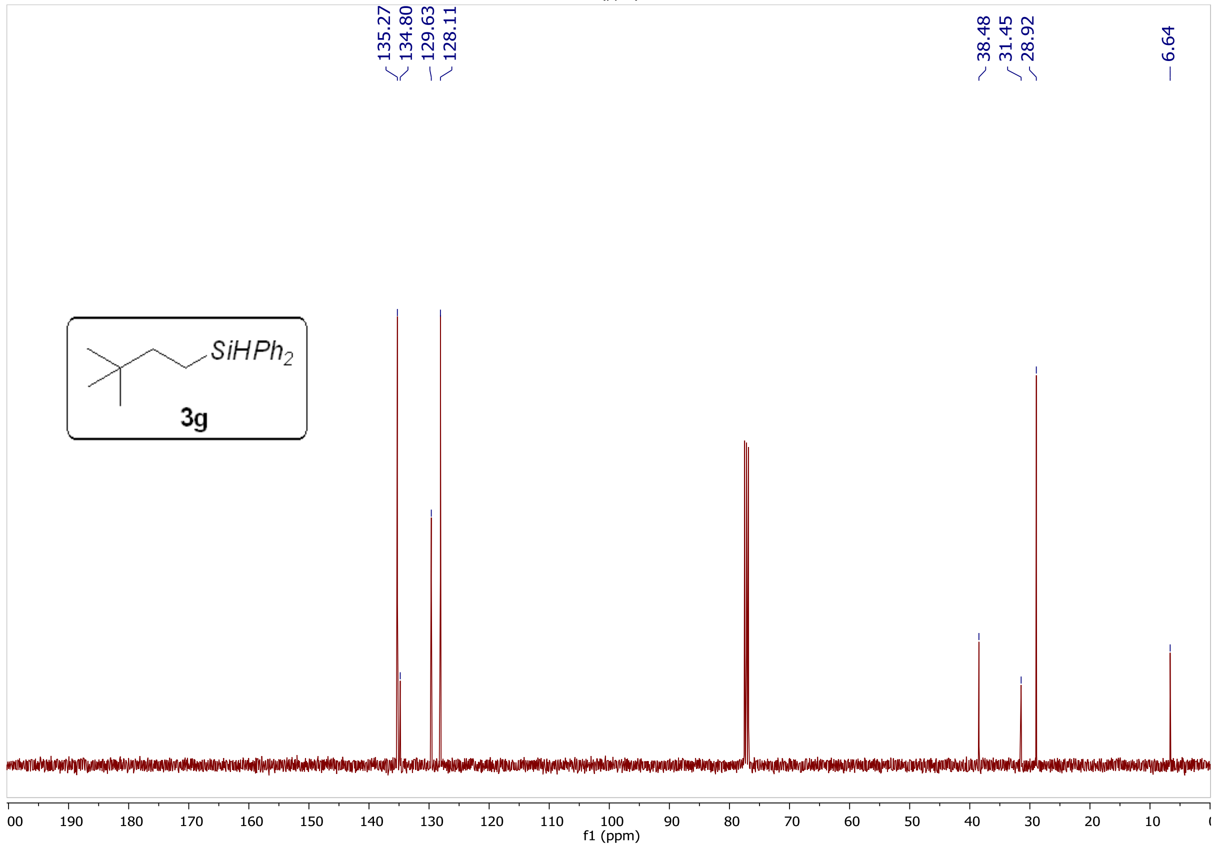
135.27 134.80 129.63 128.11
38.48 31.45 28.92
6.64
SiHPh2
3g
f1 (ppm)

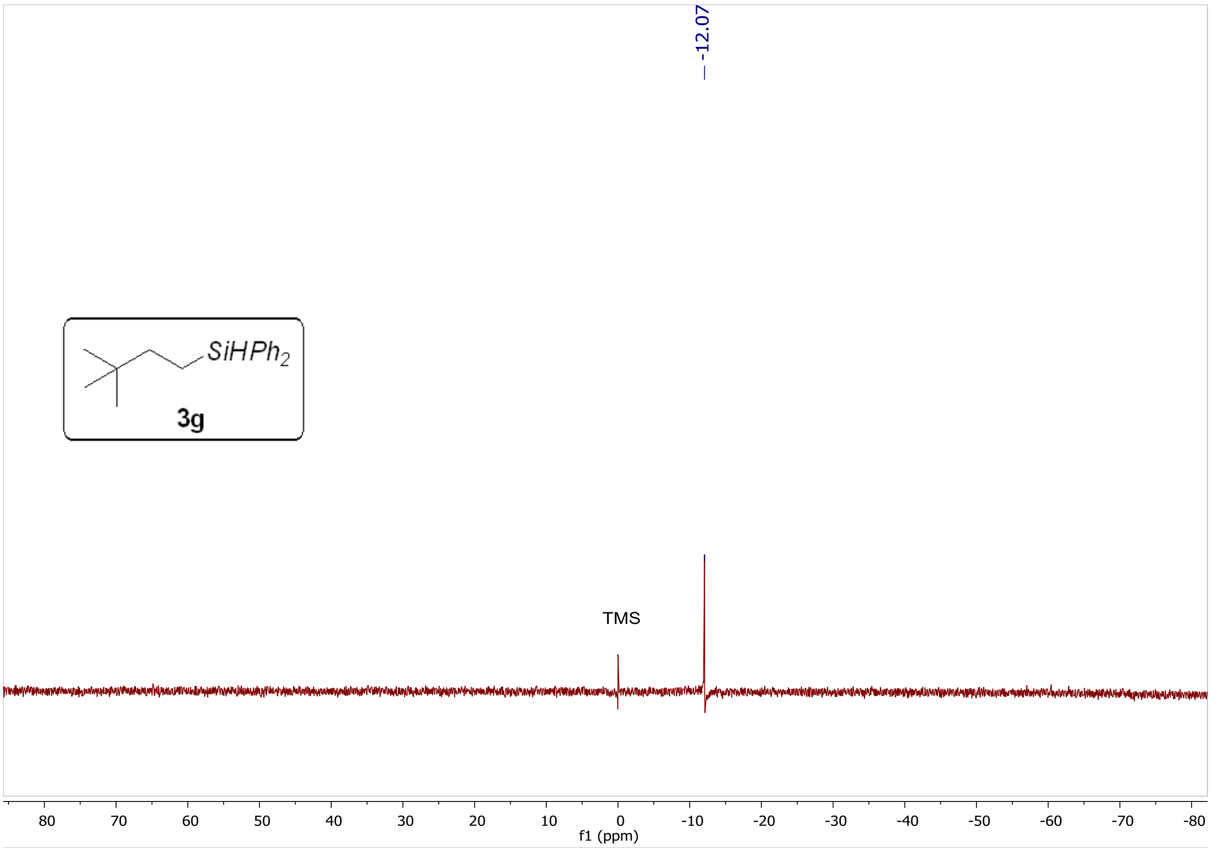

-12.07
SiHPh2
3g
TMS
f1 (ppm)

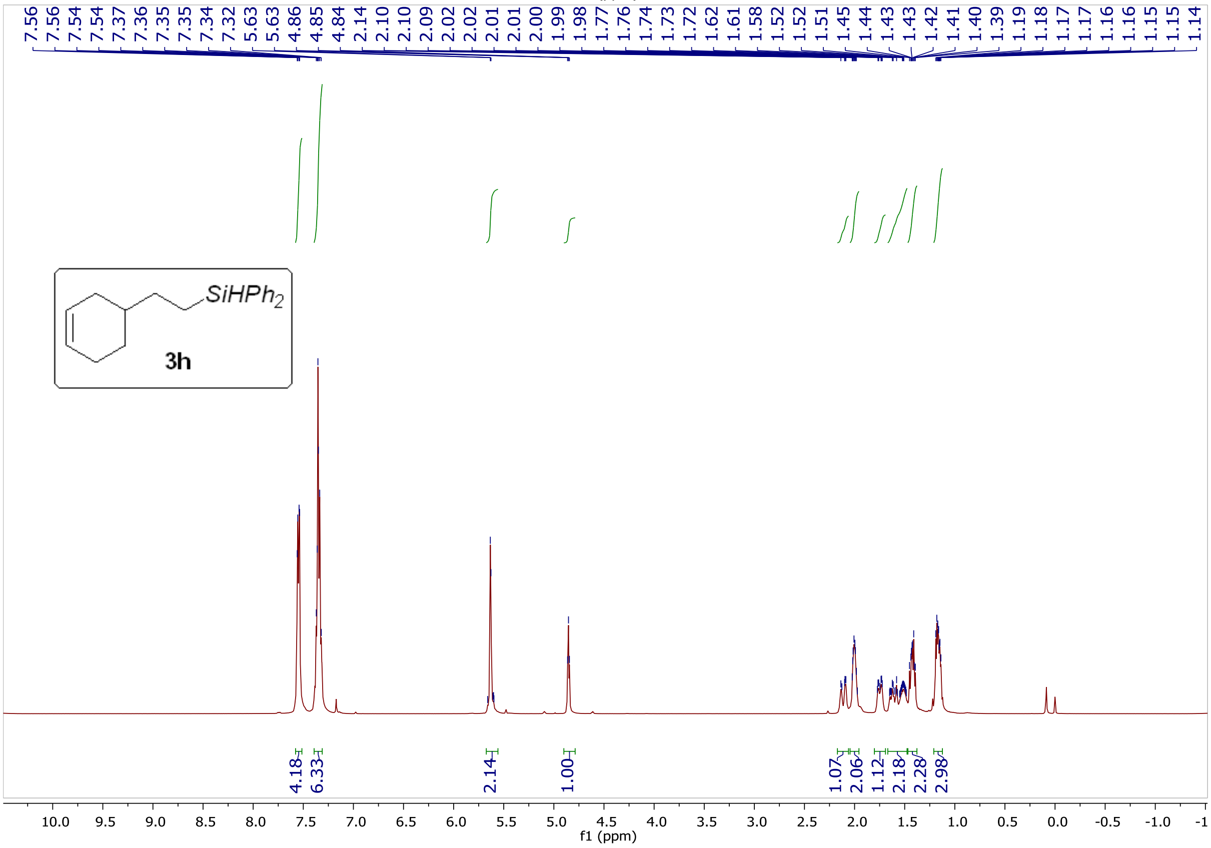

7.56 7.56 7.54 7.54 7.37 7.36 7.35 7.35 7.34 7.32 5.63 5.63 4.86 4.85 4.84 2.14 2.10 2.09 2.02 2.02 2.01 2.01 2.00 1.99 1.98 1.77 1.76 1.74 1.73 1.72 1.62 1.61 1.58 1.52 1.52 1.51 1.45 1.44 1.43 1.43 1.42 1.41 1.40 1.39 1.19 1.18 1.17 1.17 1.16 1.16 1.15 1.15 1.14
SiHPh2
3h
4.18 6.33 2.14 1.00 1.07 2.06 1.12 2.18 2.28 2.98
f1 (ppm)

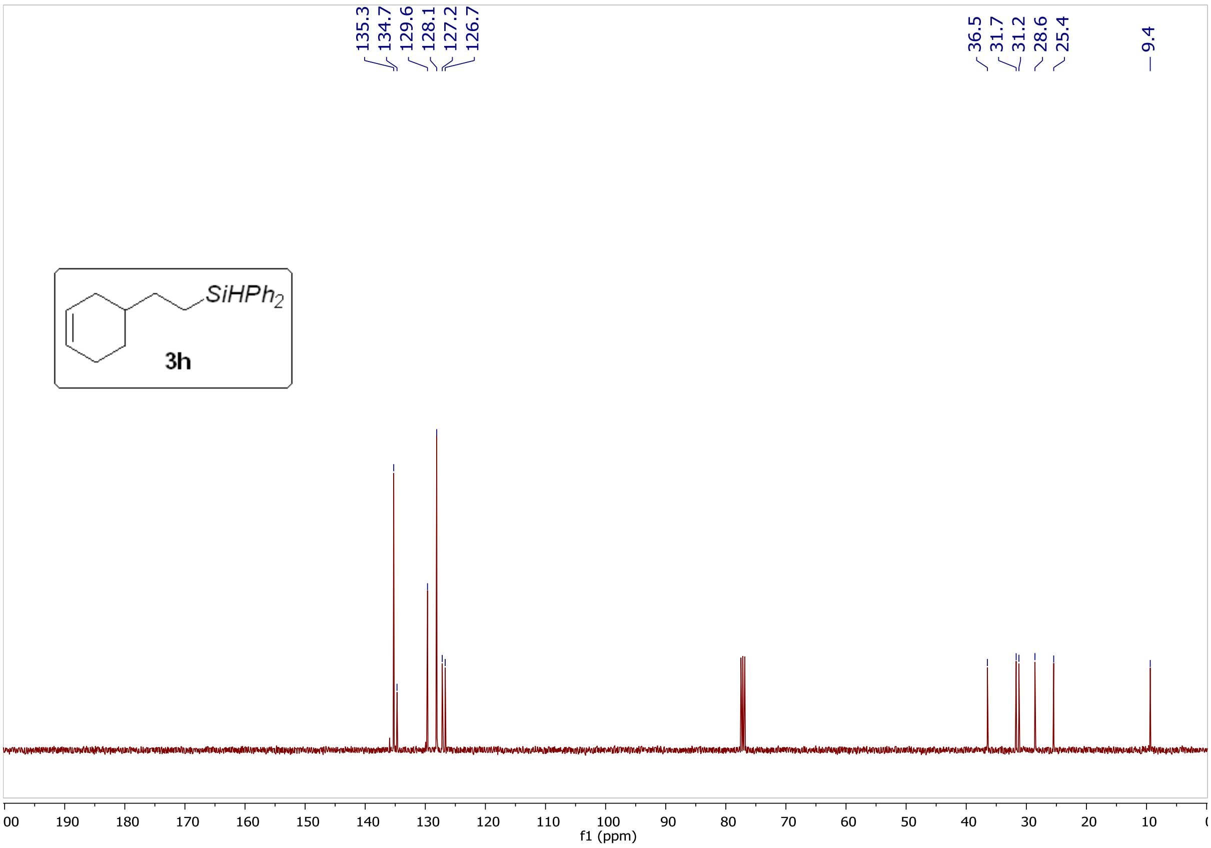
135.3
134.7
129.6
128.1
127.2
126.7
36.5
31.7
31.2
28.6
25.4
9.4
SiHPh2
3h
00 190 180 170 160 150 140 130 120 110 100 90 80 70 60 50 40 30 20 10
f1 (ppm)

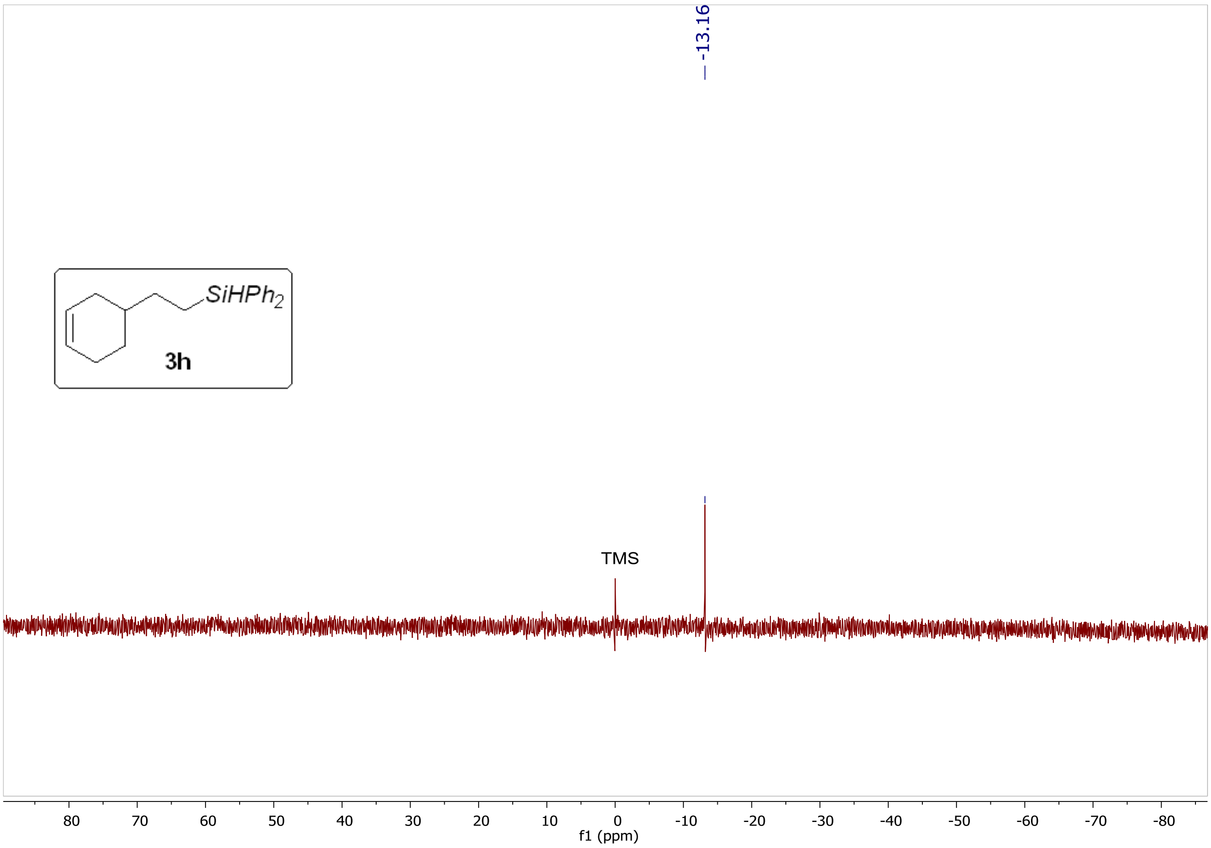
-13.16
SiHPh2
3h
TMS
80 70 60 50 40 30 20 10 0 -10 -20 -30 -40 -50 -60 -70 -80
f1 (ppm)

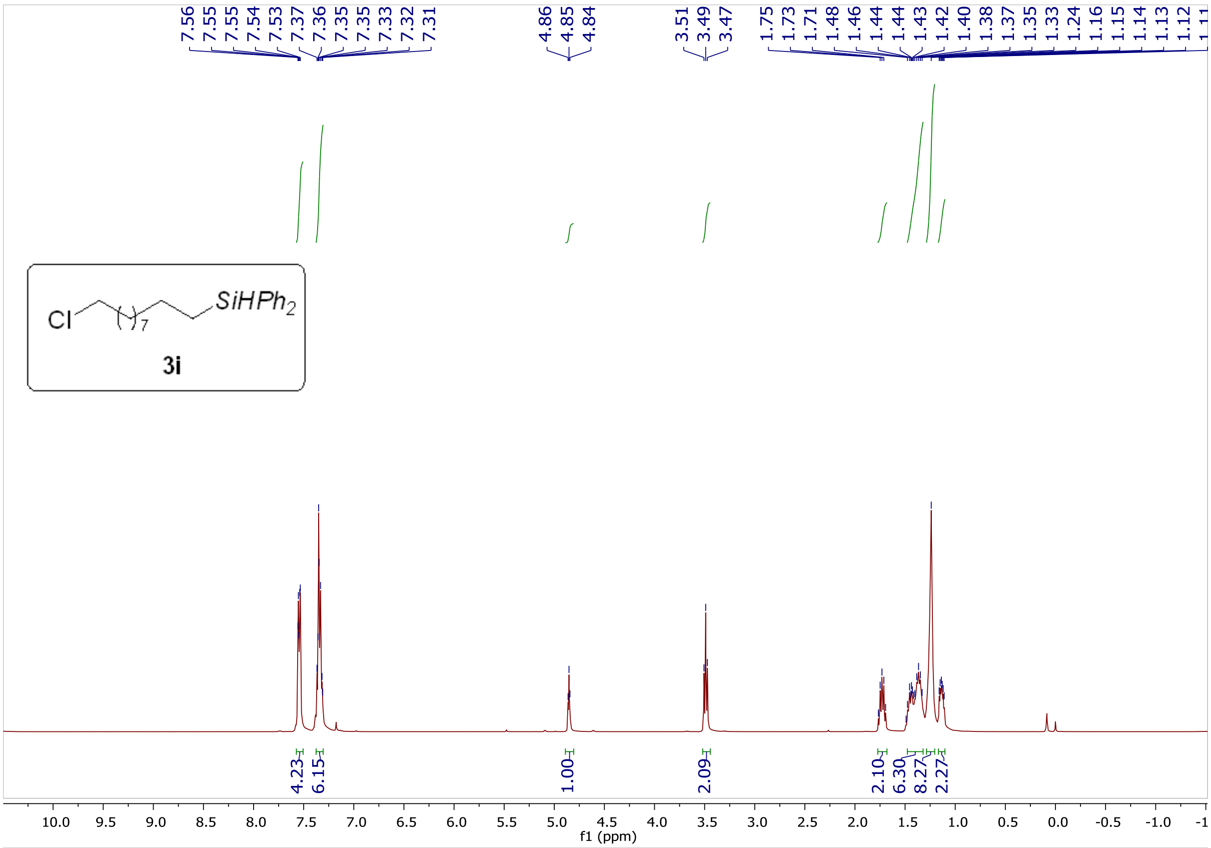
7.56 7.55 7.55 7.54 7.53 7.37 7.36 7.35 7.35 7.33 7.32 7.31
4.86 4.85 4.84
3.51 3.49 3.47
1.75 1.73 1.71 1.48 1.46 1.44 1.44 1.43 1.42 1.40 1.38 1.37 1.35 1.33 1.24 1.16 1.15 1.14 1.13 1.12
Cl
7
SiHPh2
3i
4.23 6.15 1.00 2.09 2.10 6.30 8.27 2.27
f1 (ppm)

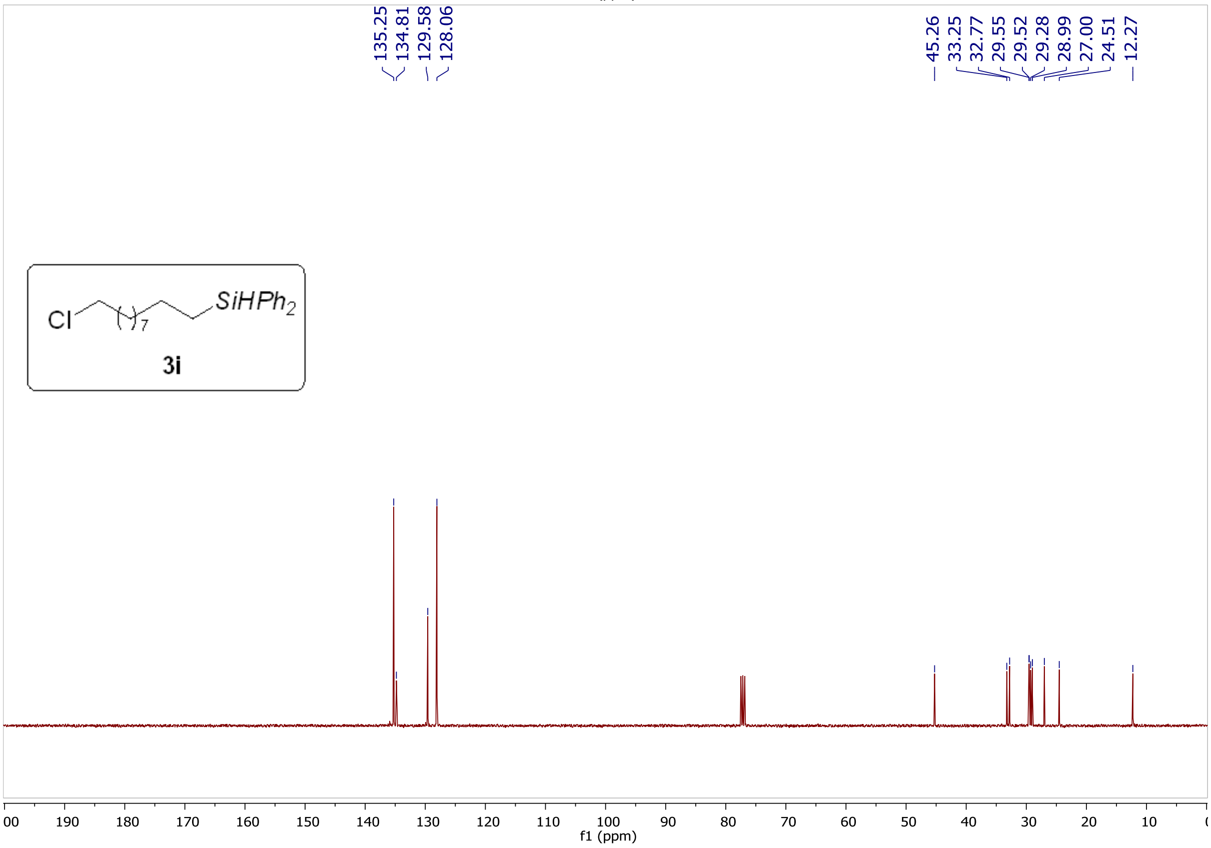
135.25 134.81 129.58 128.06
45.26 33.25 32.77 29.55 29.52 29.28 28.99 27.00 24.51 12.27
Cl
7
SiHPh2
3i
f1 (ppm)

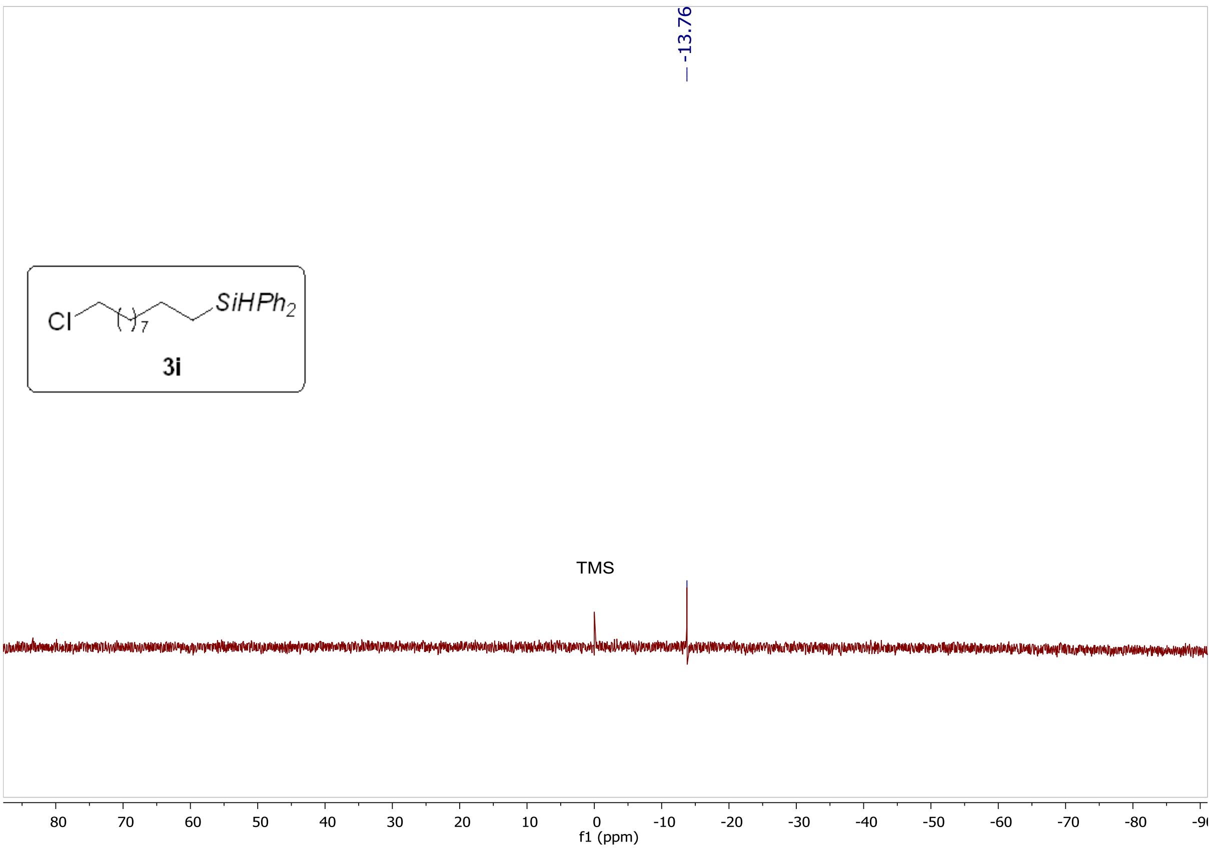
-13.76
TMS
Cl
7
SiHPh2
3i
f1 (ppm)

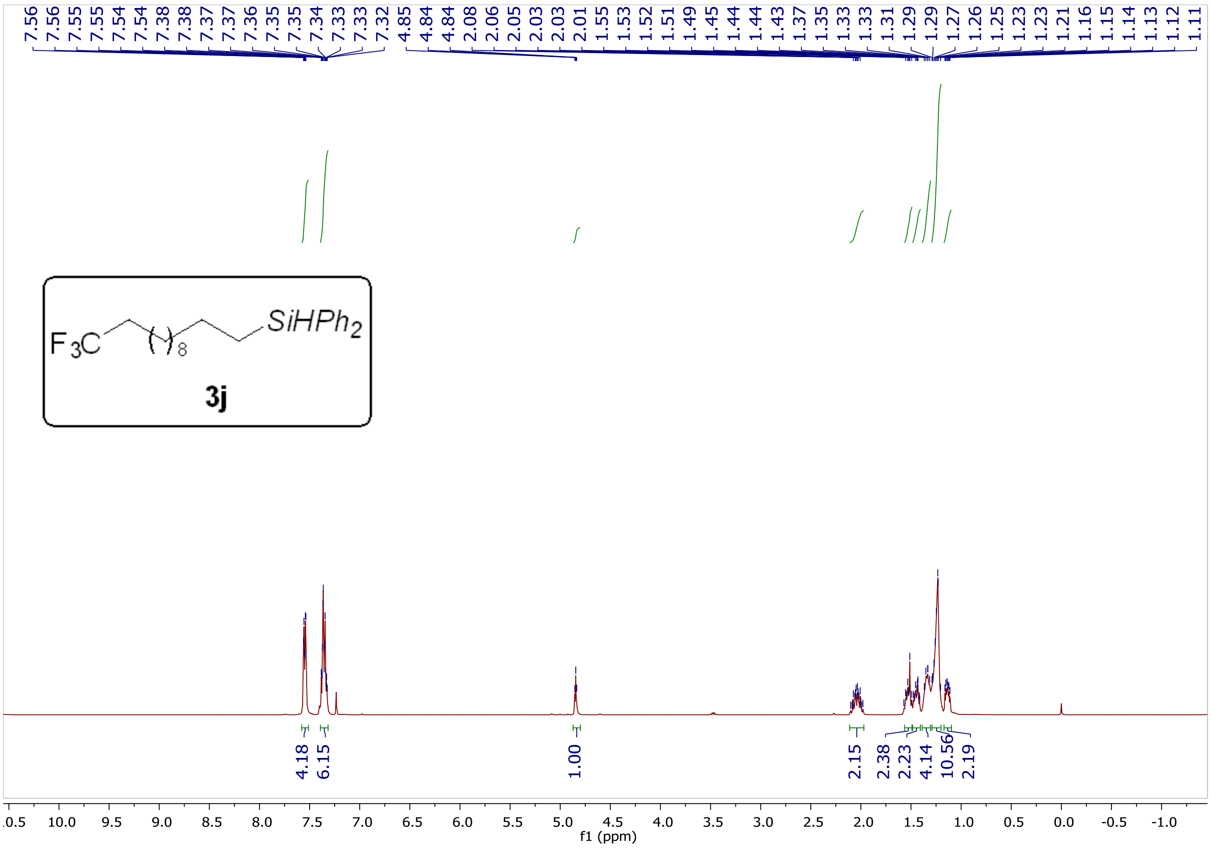
F3C
8
SiHPh2
3j
f1 (ppm)

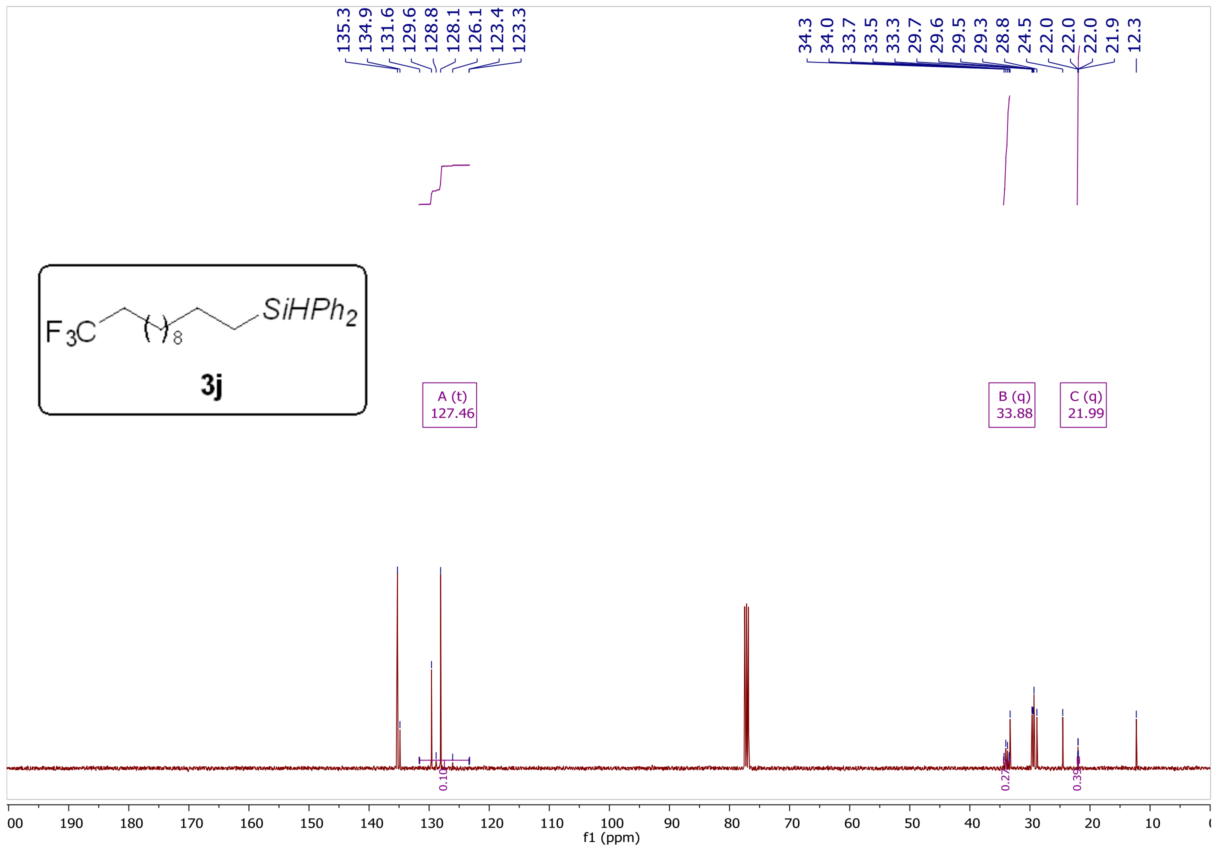
135.3
134.9
131.6
129.6
128.8
128.1
126.1
123.4
123.3
34.3
34.0
33.7
33.5
33.3
29.7
29.6
29.5
29.3
28.8
24.5
22.0
22.0
22.0
21.9
12.3
F_3C ( )8 $SiHPh_2$
3j
A (t)
127.46
B (q)
33.88
C (q)
21.99
f1 (ppm)

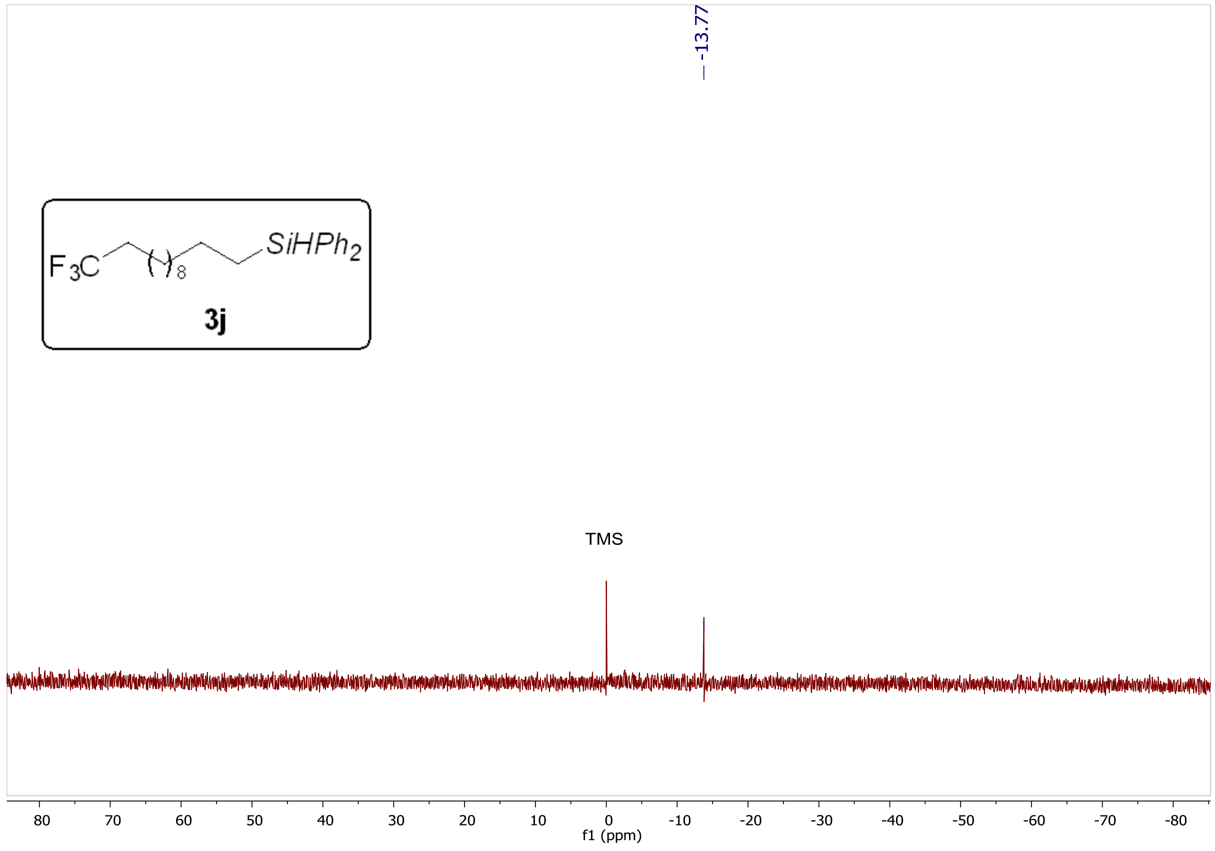
-13.77
F_3C ( )8 $SiHPh_2$
3j
TMS
f1 (ppm)

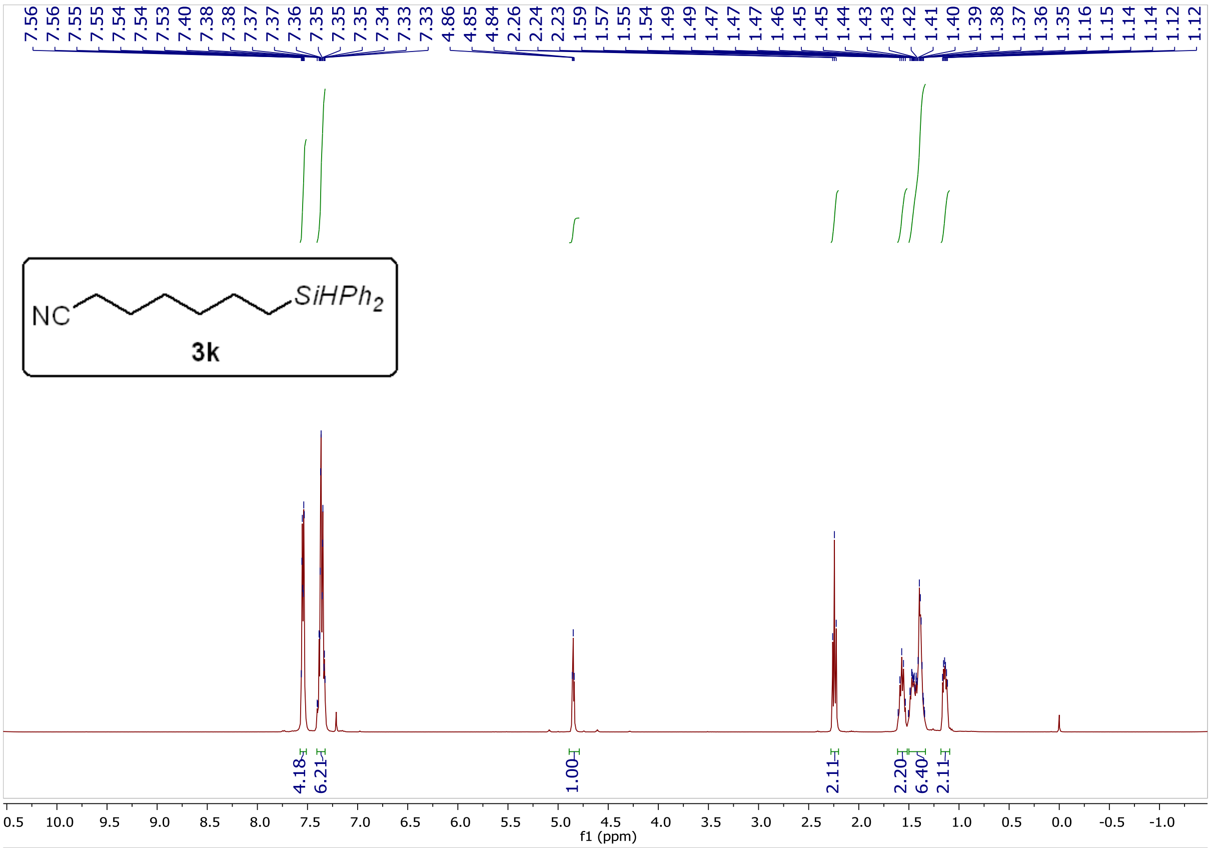
7.56 7.56 7.55 7.55 7.54 7.53 7.40 7.38 7.38 7.37 7.36 7.35 7.35 7.35 7.34 7.33 7.33 4.86 4.84 2.26 2.24 2.23 1.59 1.57 1.55 1.54 1.49 1.49 1.47 1.47 1.47 1.46 1.45 1.45 1.44 1.43 1.43 1.42 1.41 1.40 1.39 1.38 1.37 1.36 1.35 1.16 1.15 1.14 1.14 1.12 1.12
NC
SiHPh2
3k
4.18
6.21
1.00
2.11
2.20
6.40
2.11
f1 (ppm)

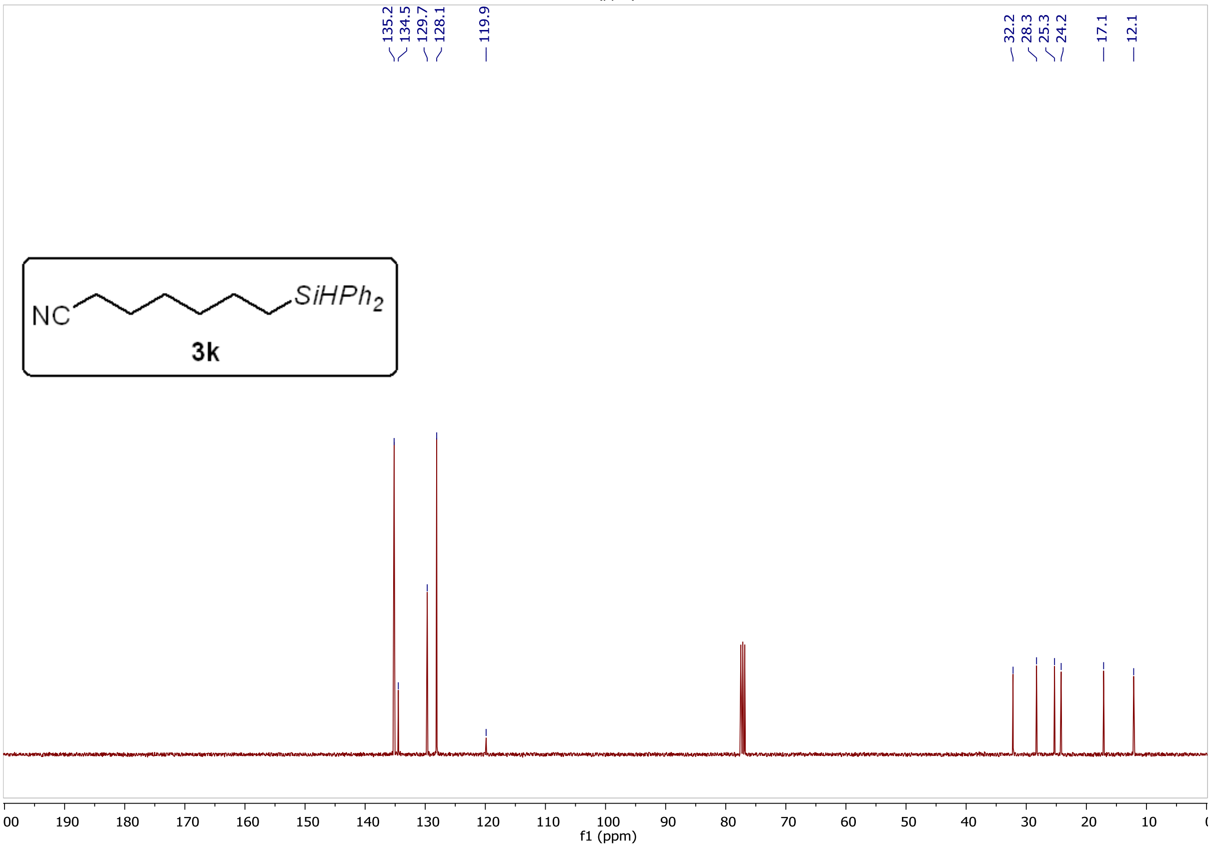
135.2 134.5 129.7 128.1 119.9
32.2 28.3 25.3 24.2 17.1 12.1
NC
SiHPh2
3k
f1 (ppm)

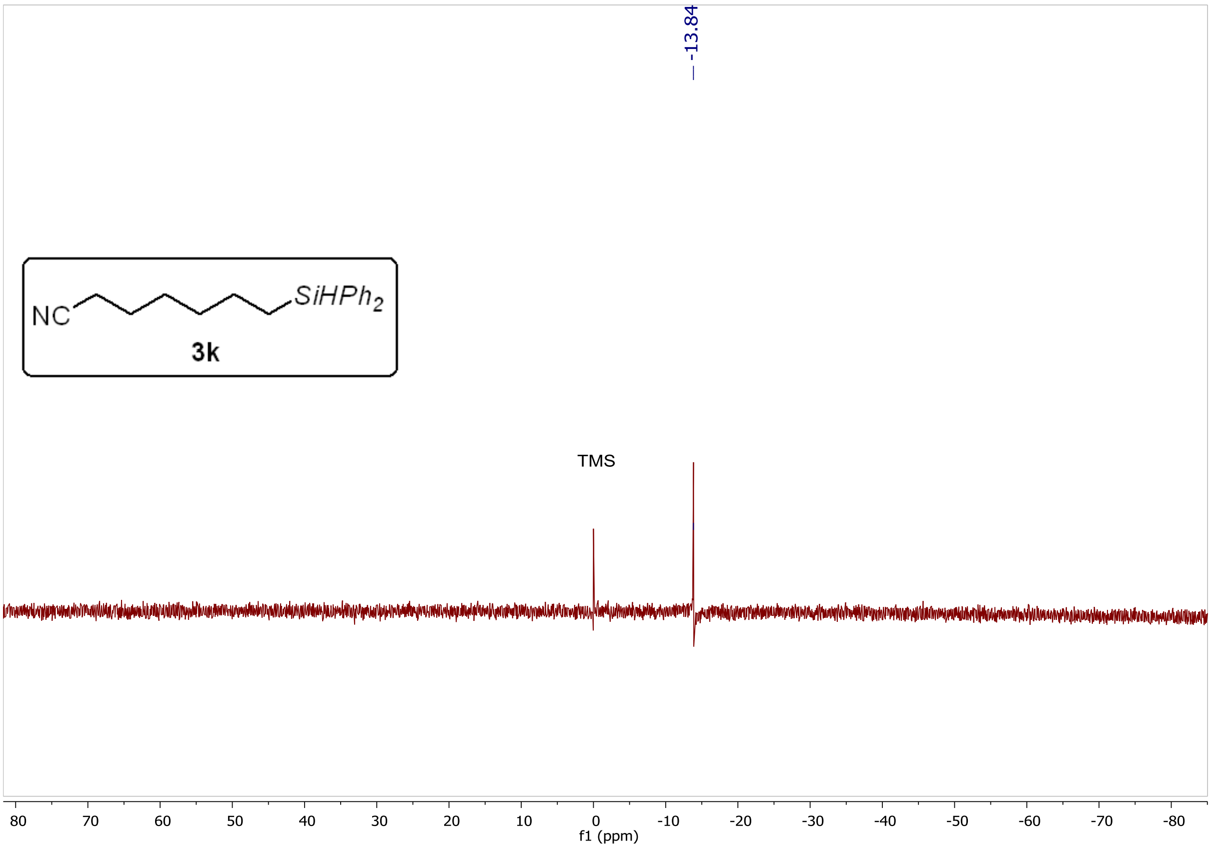
-13.84
NC
SiHPh2
3k
TMS
f1 (ppm)

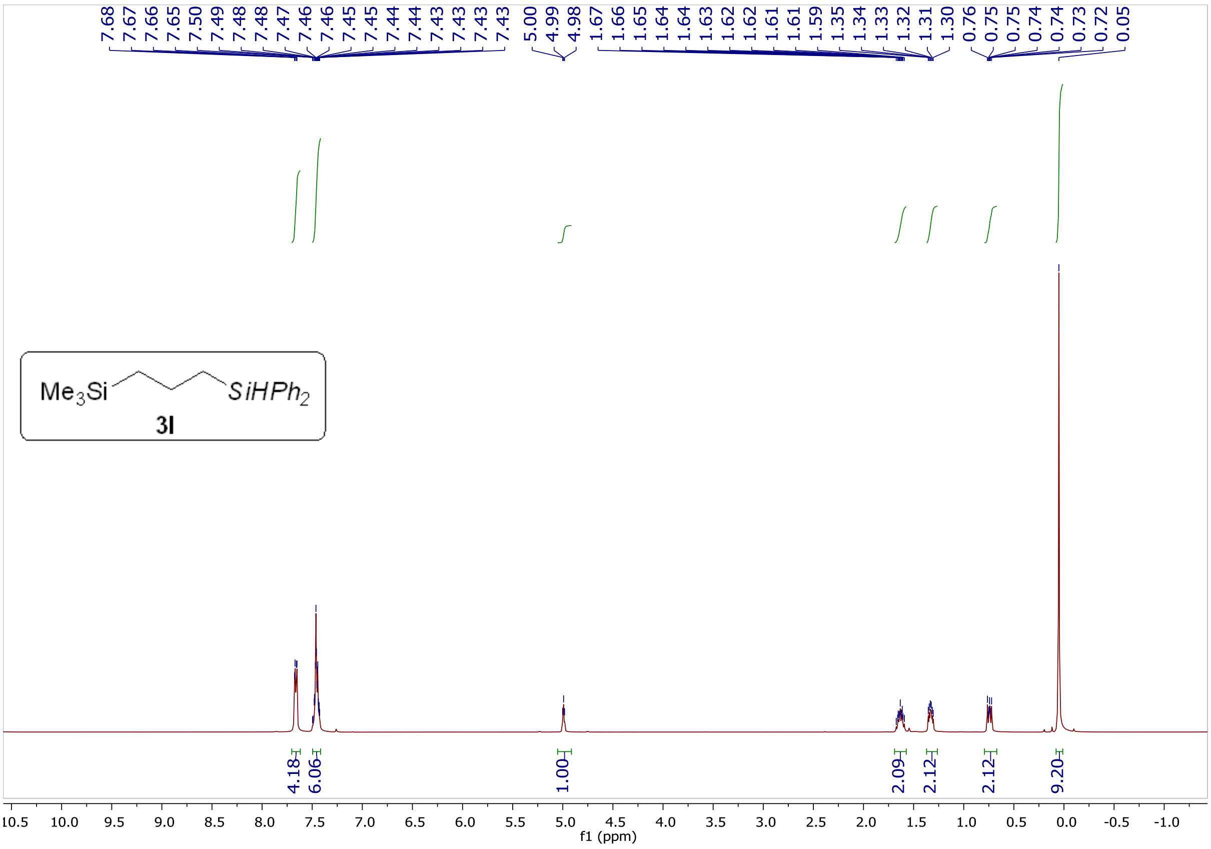
7.68
7.67
7.66
7.65
7.50
7.49
7.48
7.48
7.47
7.46
7.46
7.45
7.45
7.44
7.44
7.43
7.43
7.43
7.43
5.00
4.99
4.98
1.67
1.66
1.65
1.64
1.64
1.63
1.62
1.62
1.61
1.61
1.59
1.35
1.34
1.33
1.32
1.31
1.30
0.76
0.75
0.75
0.74
0.74
0.73
0.72
0.05
Me3Si
SiHPh2
3l
4.18
6.06
1.00
2.09
2.12
2.12
9.20
f1 (ppm)

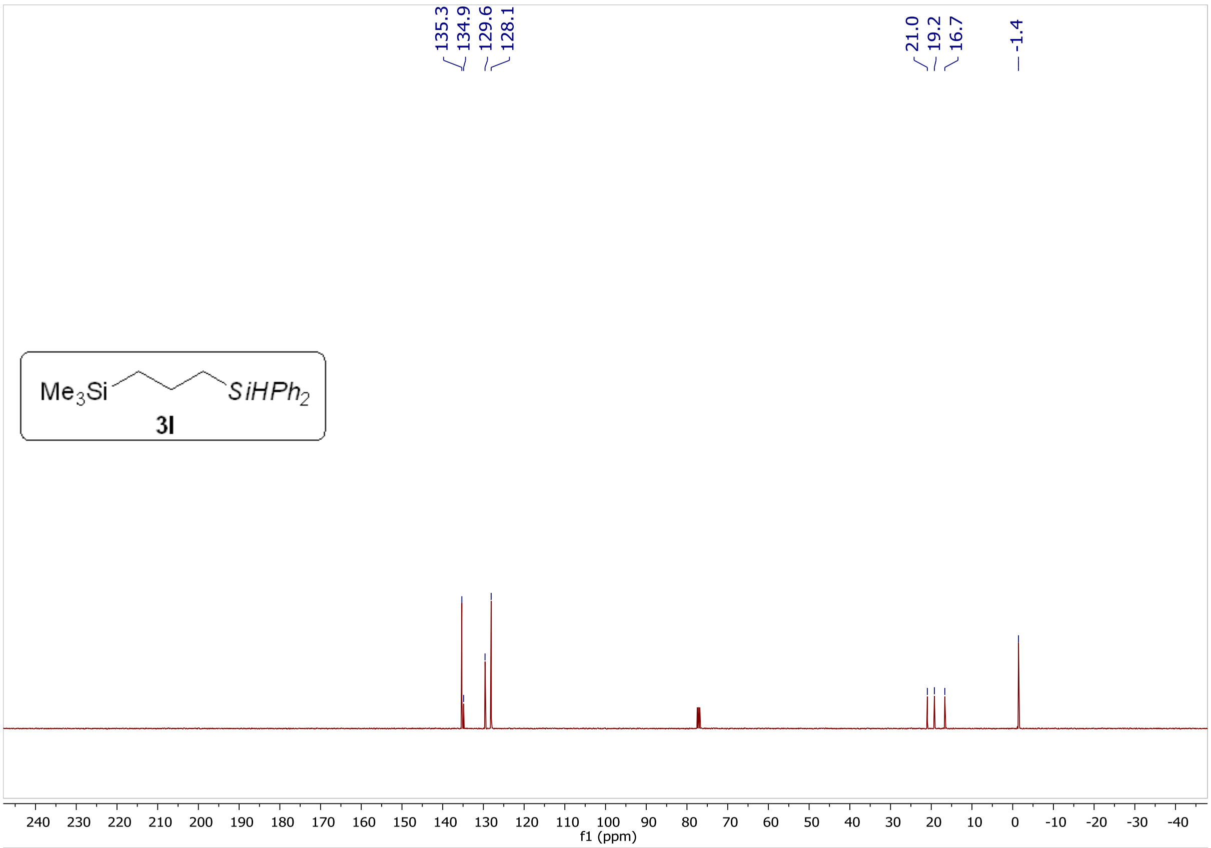
135.3
134.9
129.6
128.1
21.0
19.2
16.7
-1.4
Me3SiH
SiHPh2
3l
240
230
220
210
200
190
180
170
160
150
140
130
120
110
100
90
80
70
60
50
40
30
20
10
0
-10
-20
-30
-40
f1 (ppm)

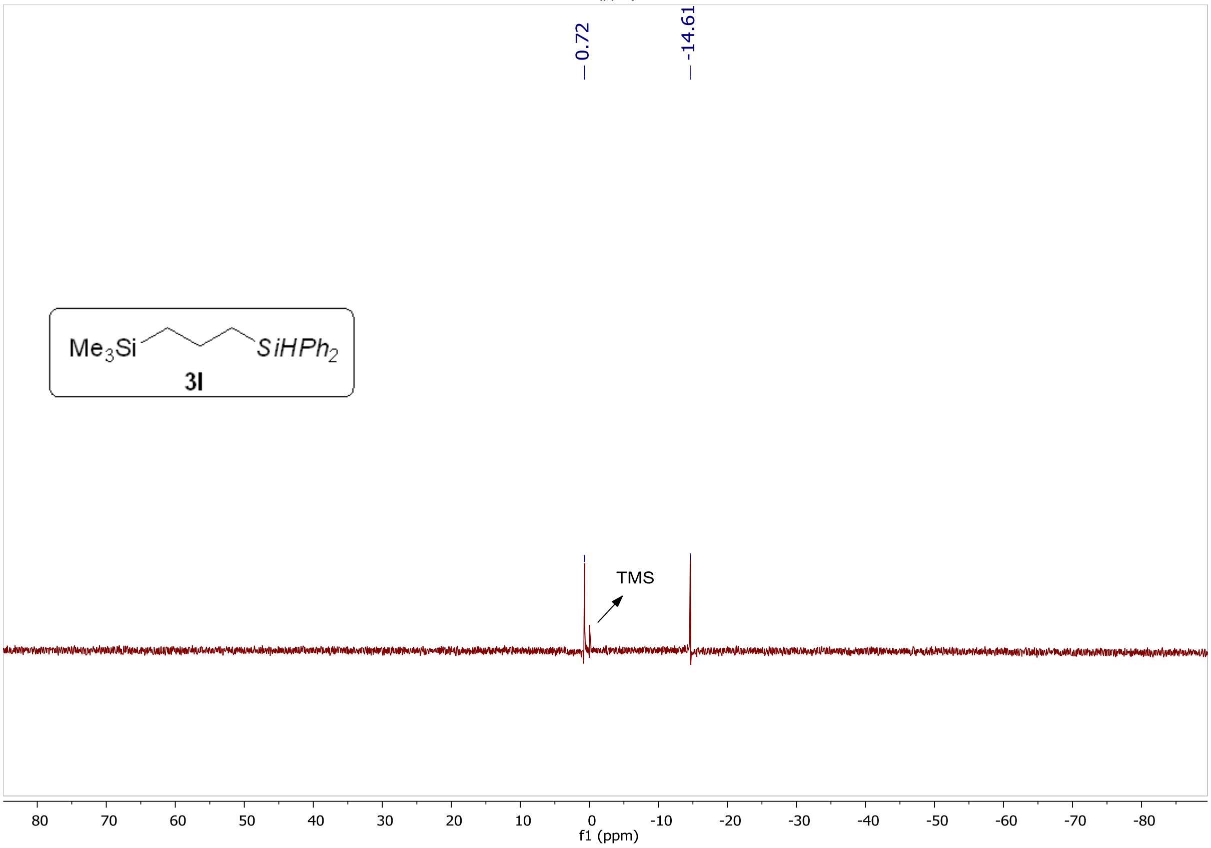
0.72
-14.61
Me3Si
SiHPh2
3l
TMS
80
70
60
50
40
30
20
10
0
-10
-20
-30
-40
-50
-60
-70
-80
f1 (ppm)

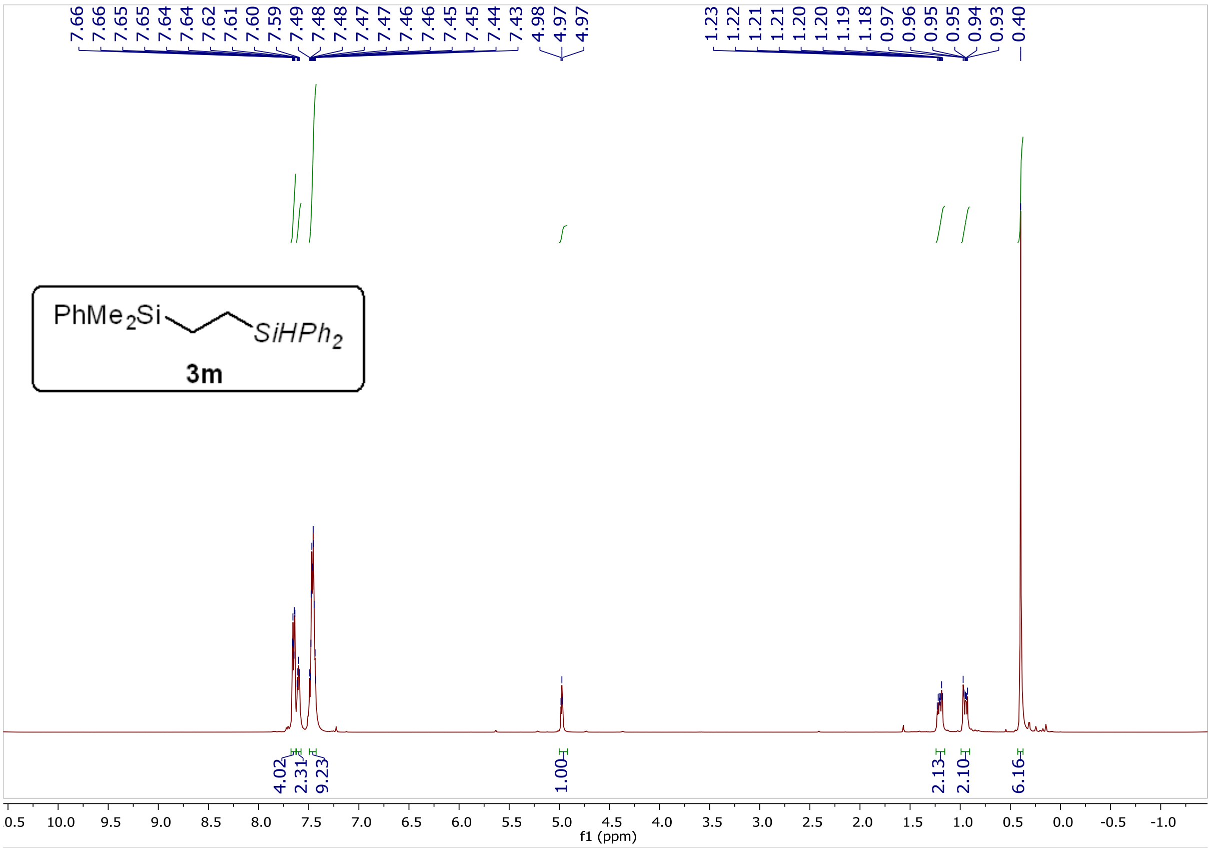
7.66
7.66
7.65
7.65
7.64
7.64
7.62
7.61
7.60
7.59
7.49
7.48
7.48
7.47
7.46
7.46
7.45
7.45
7.44
7.43
4.98
4.97
4.97
1.23
1.22
1.21
1.21
1.20
1.20
1.19
1.18
0.97
0.96
0.95
0.95
0.94
0.93
0.40
PhMe2Si SiHPh2
3m
4.02
2.31
9.23
1.00
2.13
2.10
6.16
f1 (ppm)

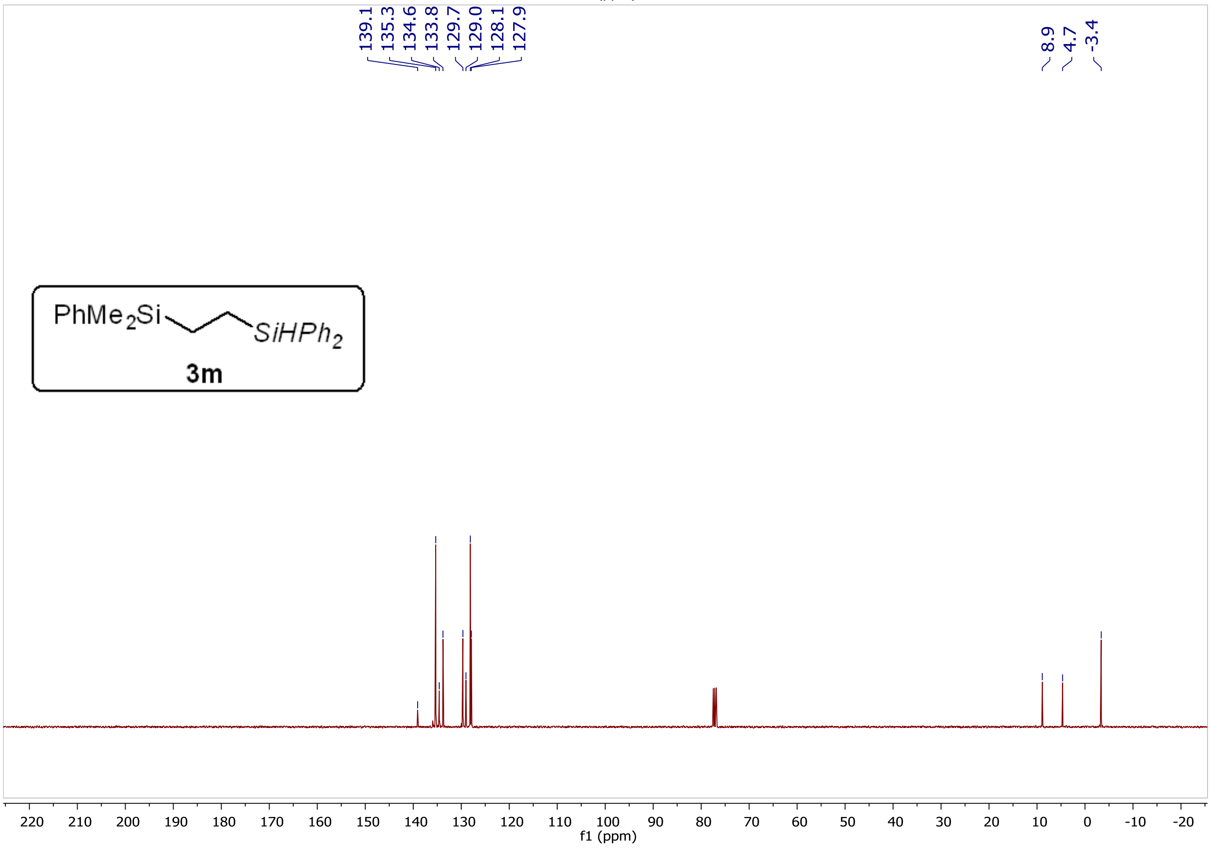
139.1
135.3
134.6
133.8
129.7
129.0
128.1
127.9
8.9
4.7
-3.4
PhMe2Si SiHPh2
3m
f1 (ppm)

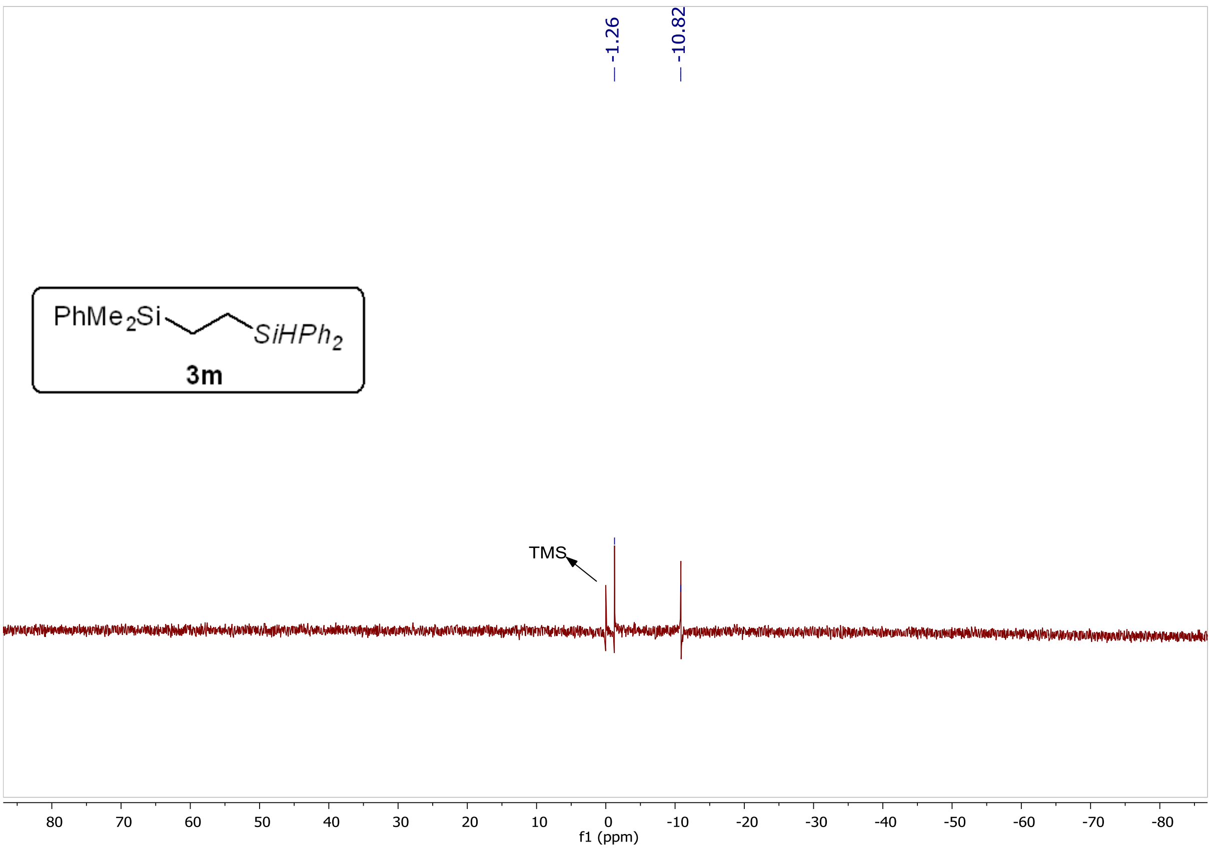
-1.26
-10.82
PhMe2Si
SiHPh2
3m
TMS
80 70 60 50 40 30 20 10 0 -10 -20 -30 -40 -50 -60 -70 -80
f1 (ppm)

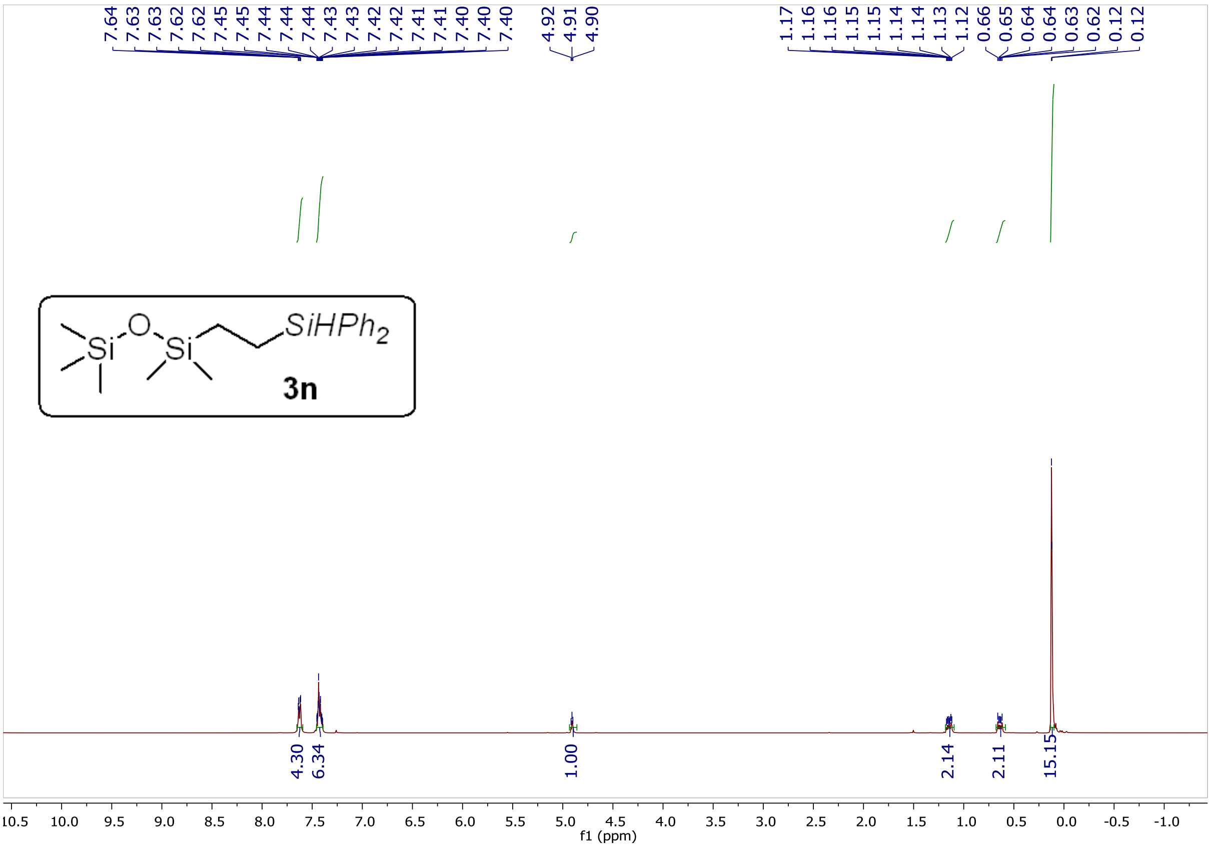
7.64 7.63 7.63 7.62 7.62 7.45 7.45 7.44 7.44 7.44 7.43 7.43 7.42 7.42 7.41 7.41 7.40 7.40 7.40
4.92 4.91 4.90
1.17 1.16 1.16 1.15 1.15 1.14 1.14 1.13 1.12 0.66 0.65 0.64 0.64 0.63 0.62 0.12 0.12
Si O Si
SiHPh2
3n
4.30 6.34 1.00 2.14 2.11 15.15
10.5 10.0 9.5 9.0 8.5 8.0 7.5 7.0 6.5 6.0 5.5 5.0 4.5 4.0 3.5 3.0 2.5 2.0 1.5 1.0 0.5 0.0 -0.5 -1.0
f1 (ppm)

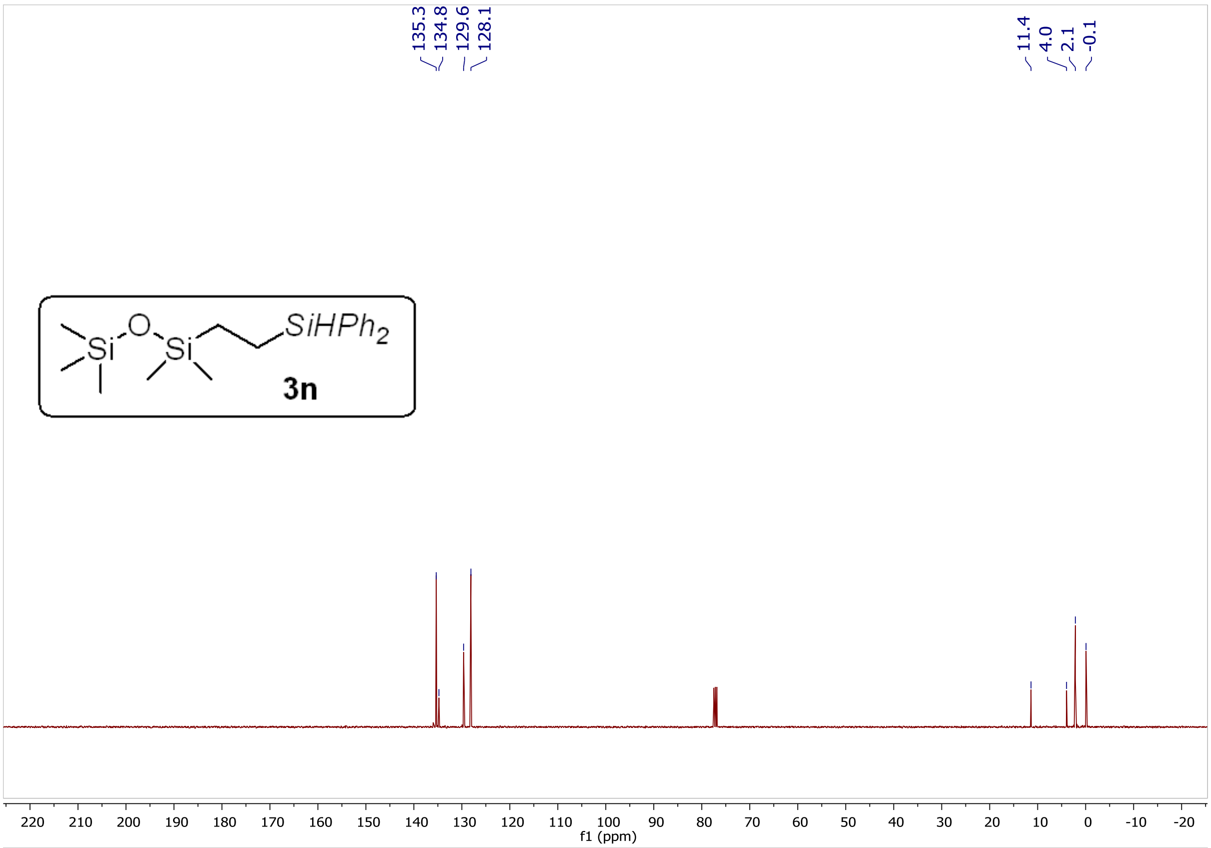
135.3
134.8
129.6
128.1
11.4
4.0
2.1
-0.1
SiHPh2
O
Si
Si
3n
f1 (ppm)

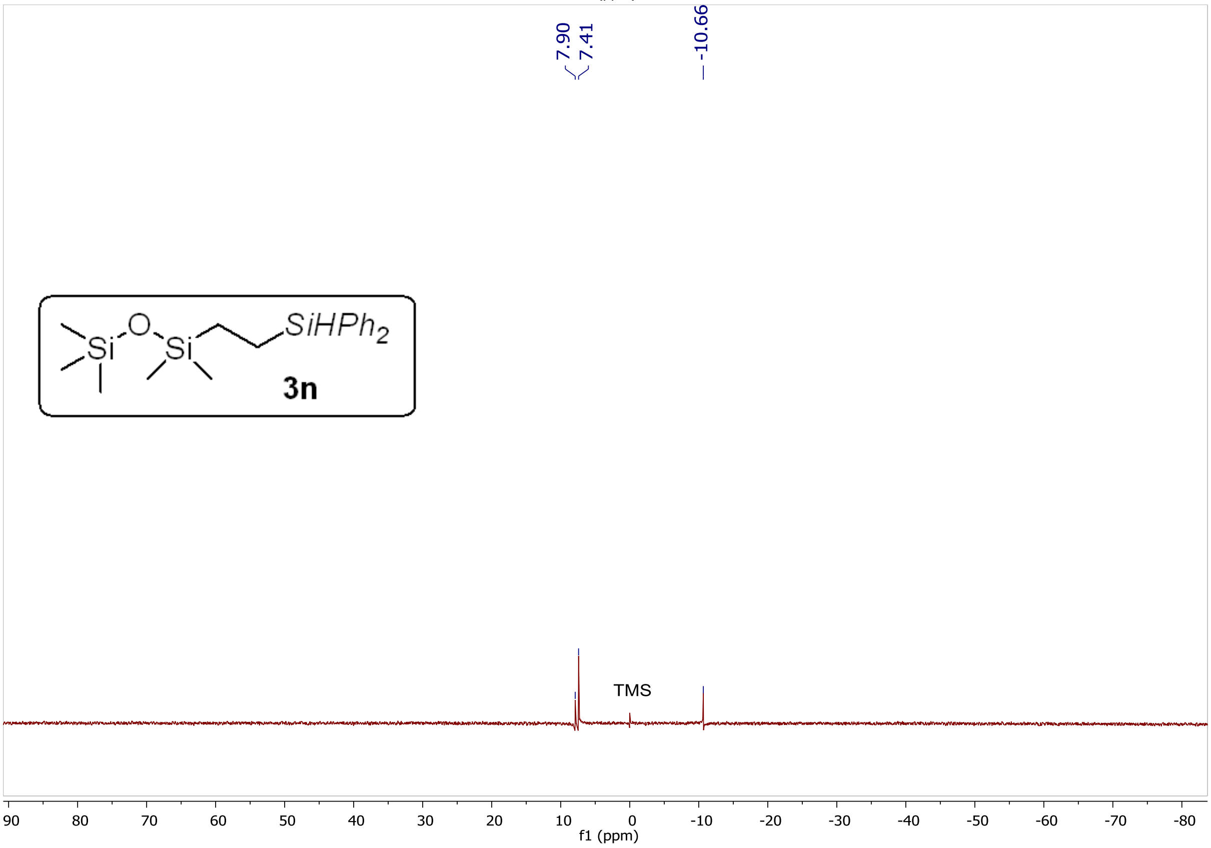
7.90
7.41
-10.66
SiHPh2
O
Si
Si
3n
TMS
f1 (ppm)

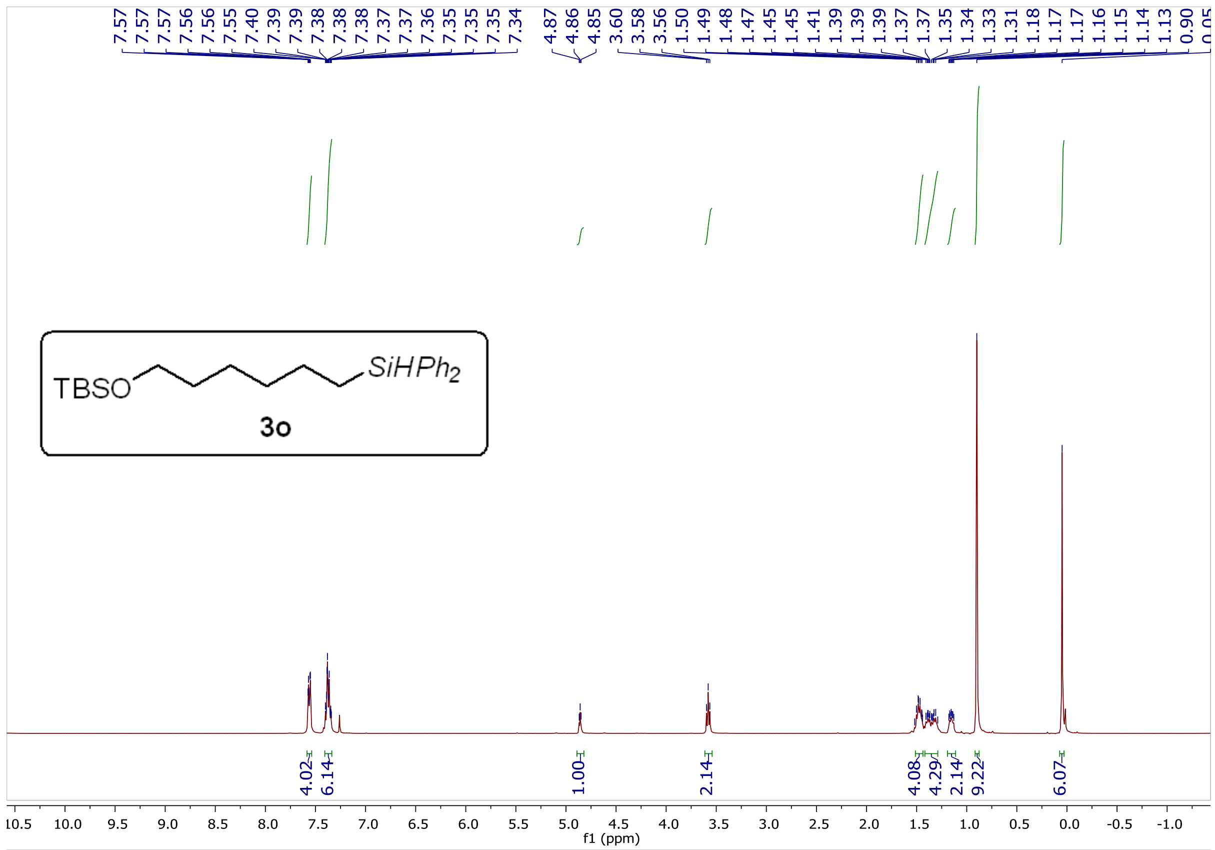

TBSO
SiHPh2
3o
7.57
7.57
7.56
7.56
7.55
7.40
7.39
7.39
7.38
7.38
7.38
7.37
7.37
7.36
7.35
7.35
7.35
7.34
4.87
4.86
4.85
3.60
3.58
3.56
1.50
1.49
1.48
1.47
1.45
1.45
1.41
1.39
1.39
1.39
1.37
1.37
1.35
1.34
1.33
1.31
1.18
1.17
1.17
1.16
1.15
1.14
1.13
0.90
4.02
6.14
1.00
2.14
4.08
4.29
2.14
9.22
6.07
f1 (ppm)

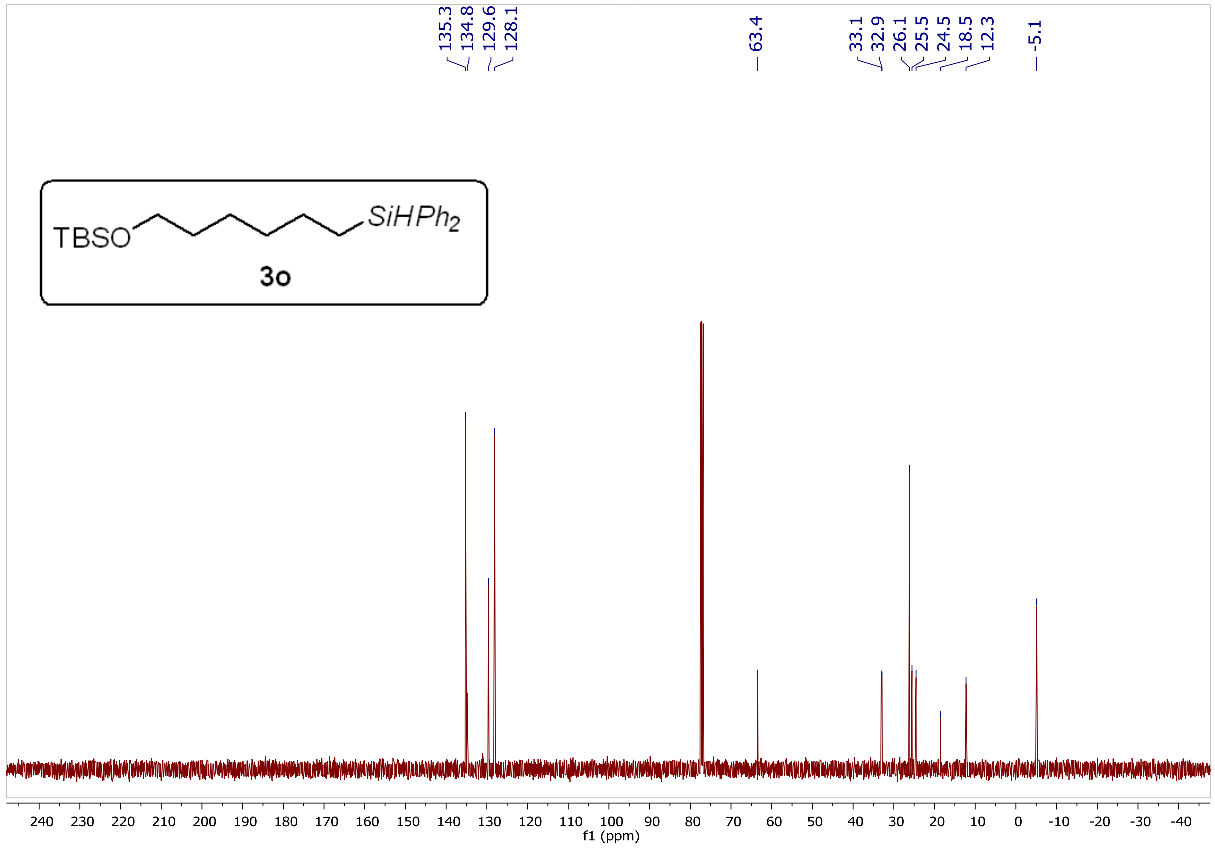

TBSO
SiHPh2
3o
135.3
134.8
129.6
128.1
63.4
33.1
32.9
26.1
25.5
24.5
18.5
12.3
-5.1
f1 (ppm)

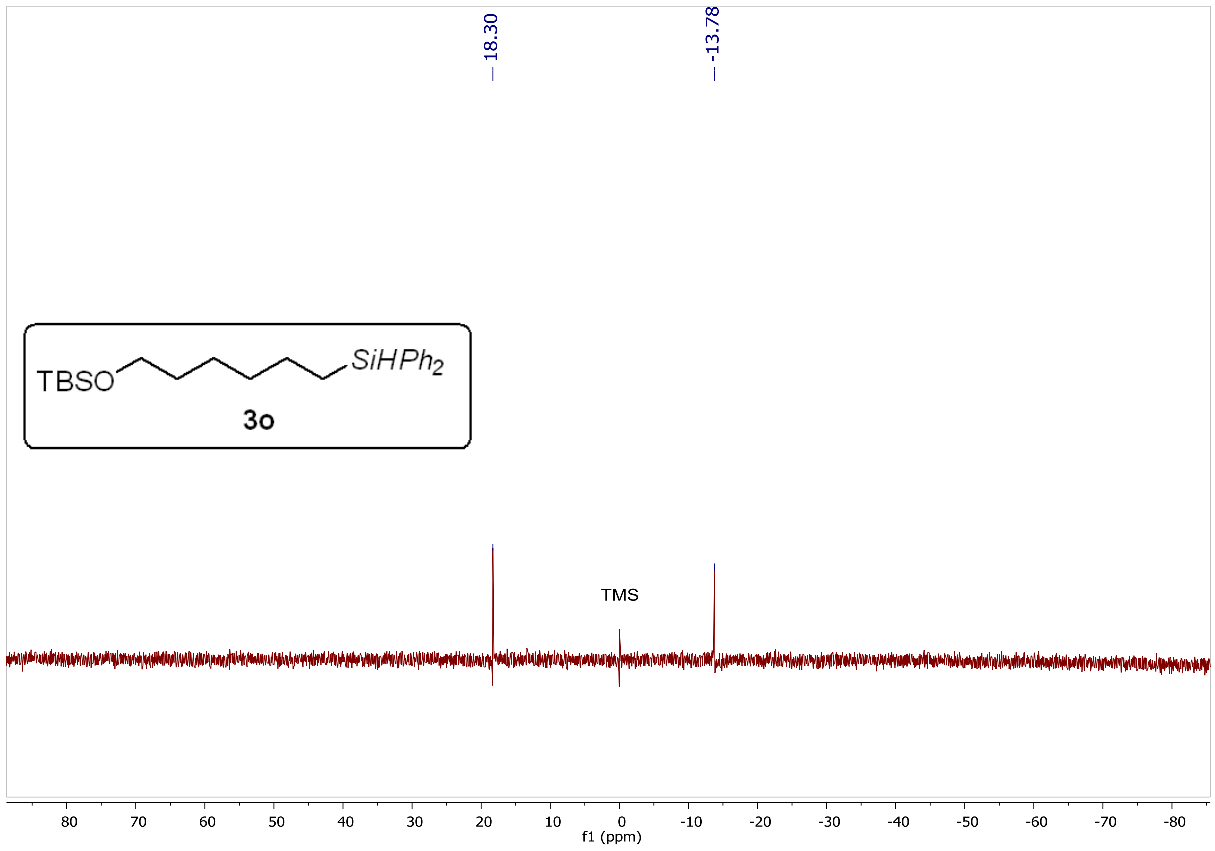

18.30
-13.78
TBSO
SiHPh2
3o
TMS
f1 (ppm)

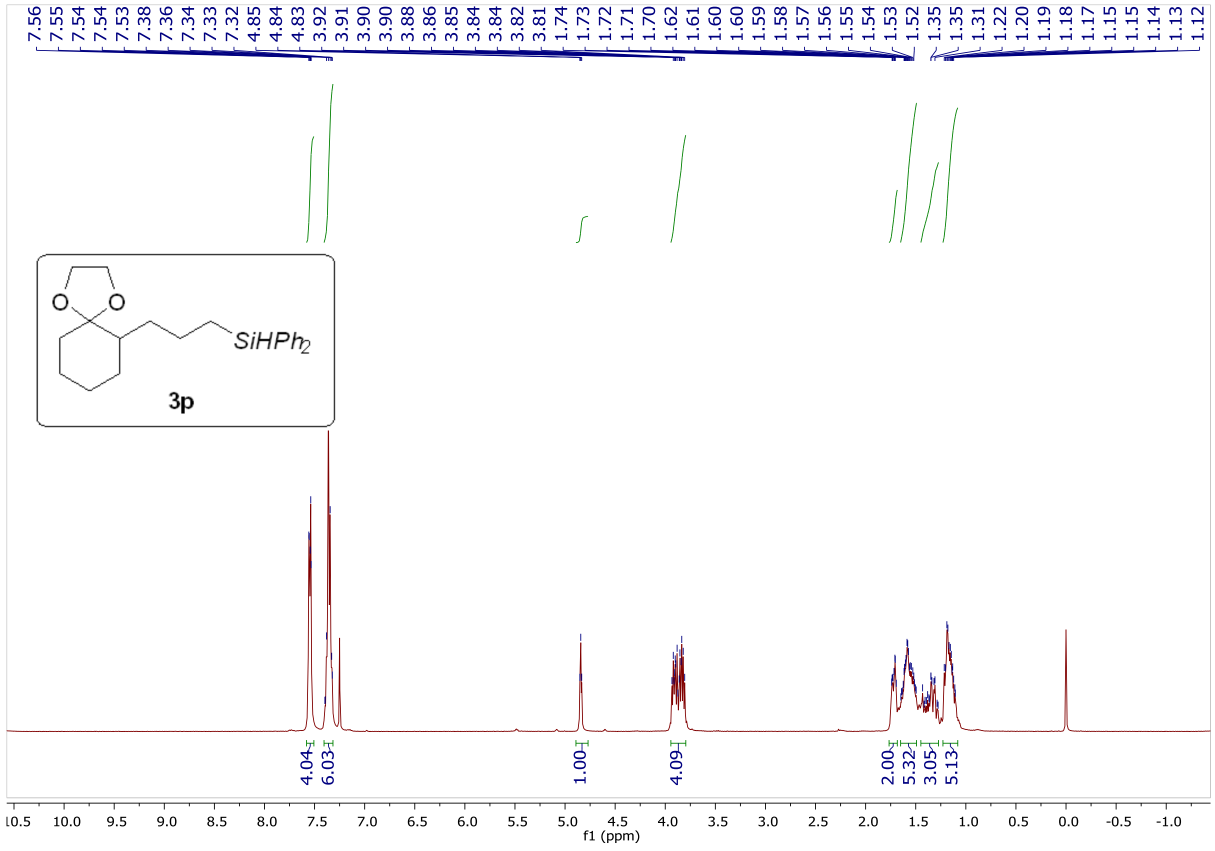

7.56 7.55 7.54 7.54 7.53 7.38 7.36 7.34 7.33 7.32 4.85 4.84 4.83 3.92 3.91 3.90 3.90 3.88 3.86 3.85 3.84 3.84 3.82 3.81 1.74 1.73 1.72 1.71 1.70 1.62 1.61 1.60 1.60 1.59 1.58 1.57 1.56 1.55 1.54 1.53 1.52 1.35 1.35 1.31 1.22 1.20 1.19 1.18 1.17 1.15 1.15 1.14 1.13 1.12
O O
SiHPh2
3p
4.04 6.03 1.00 4.09 2.00 5.32 3.05 5.13
f1 (ppm)

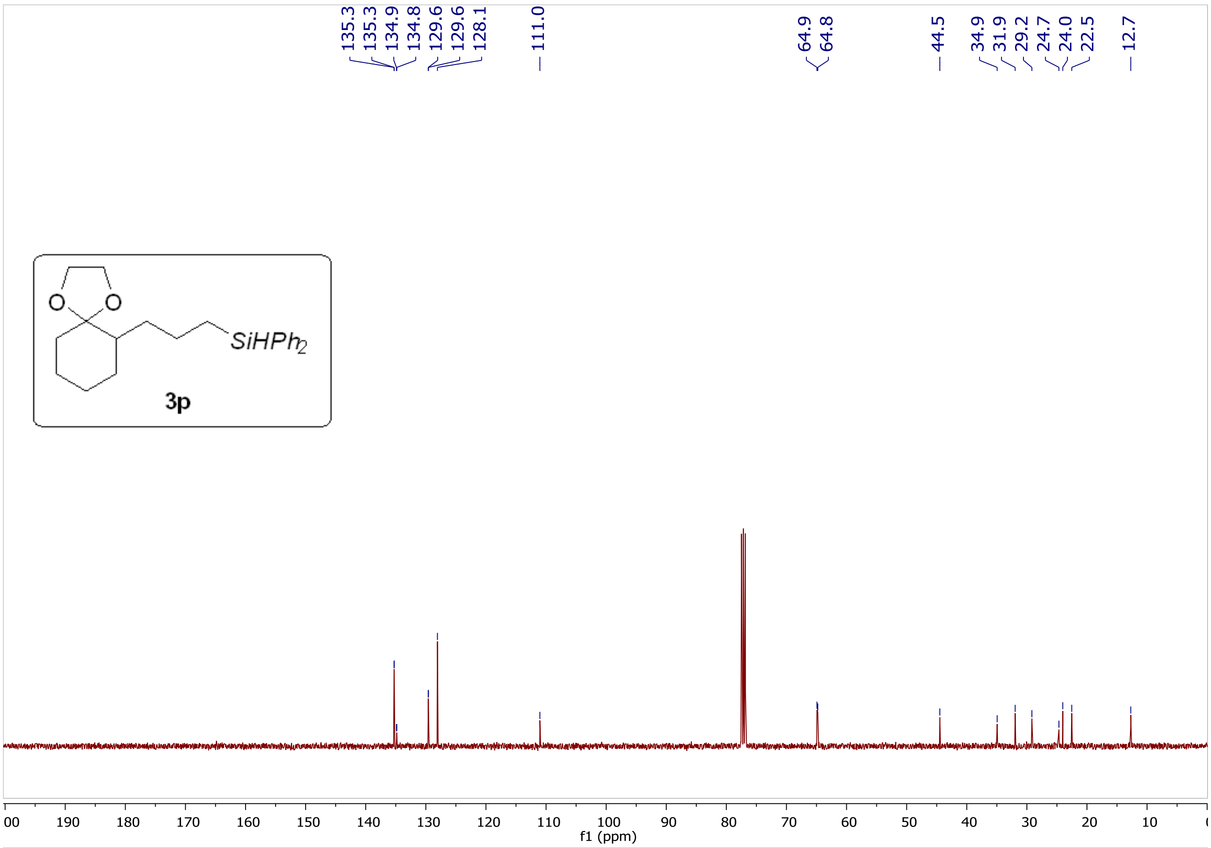
135.3
135.3
134.9
134.8
129.6
129.6
128.1
111.0
64.9
64.8
44.5
34.9
31.9
29.2
24.7
24.0
22.5
12.7
O
O
SiHPh2
3p
190
180
170
160
150
140
130
120
110
100
90
80
70
60
50
40
30
20
10
f1 (ppm)

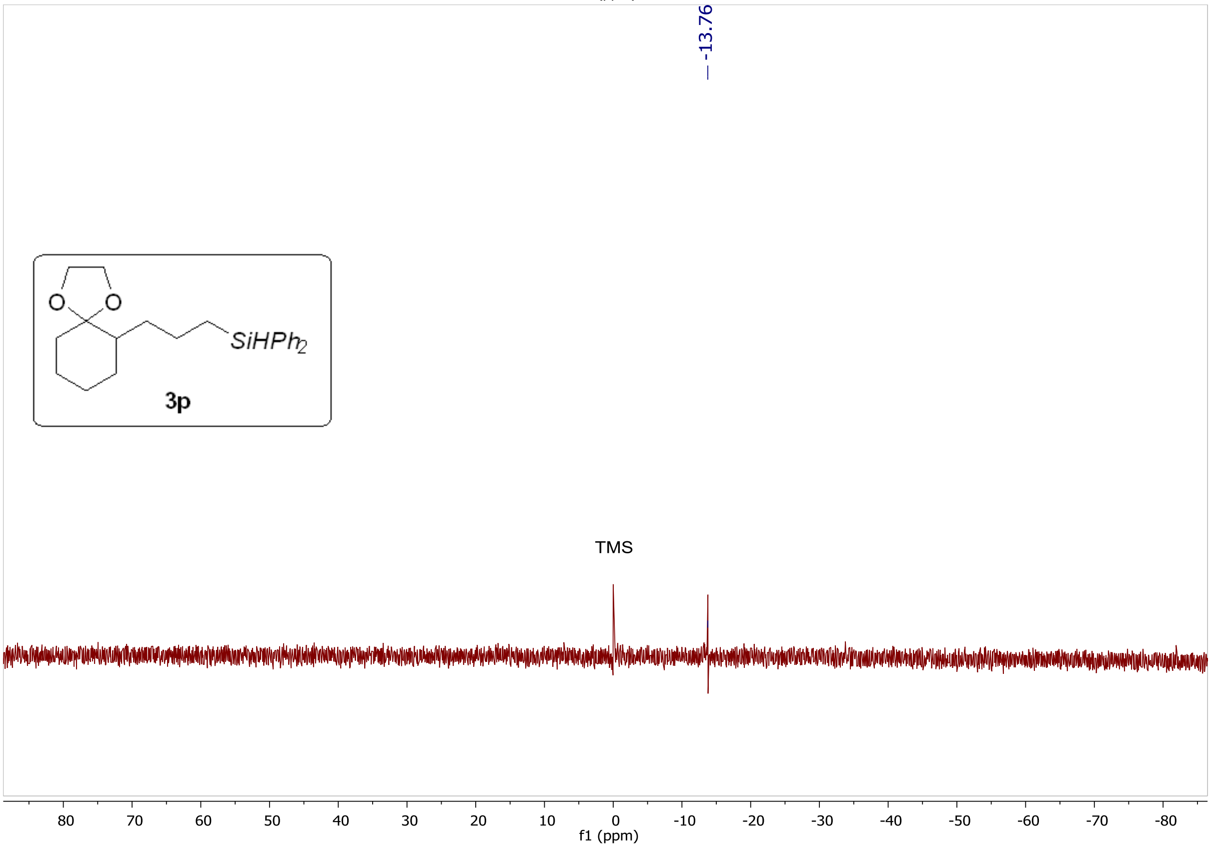
-13.76
O
O
SiHPh2
3p
TMS
80
70
60
50
40
30
20
10
0
-10
-20
-30
-40
-50
-60
-70
-80
f1 (ppm)

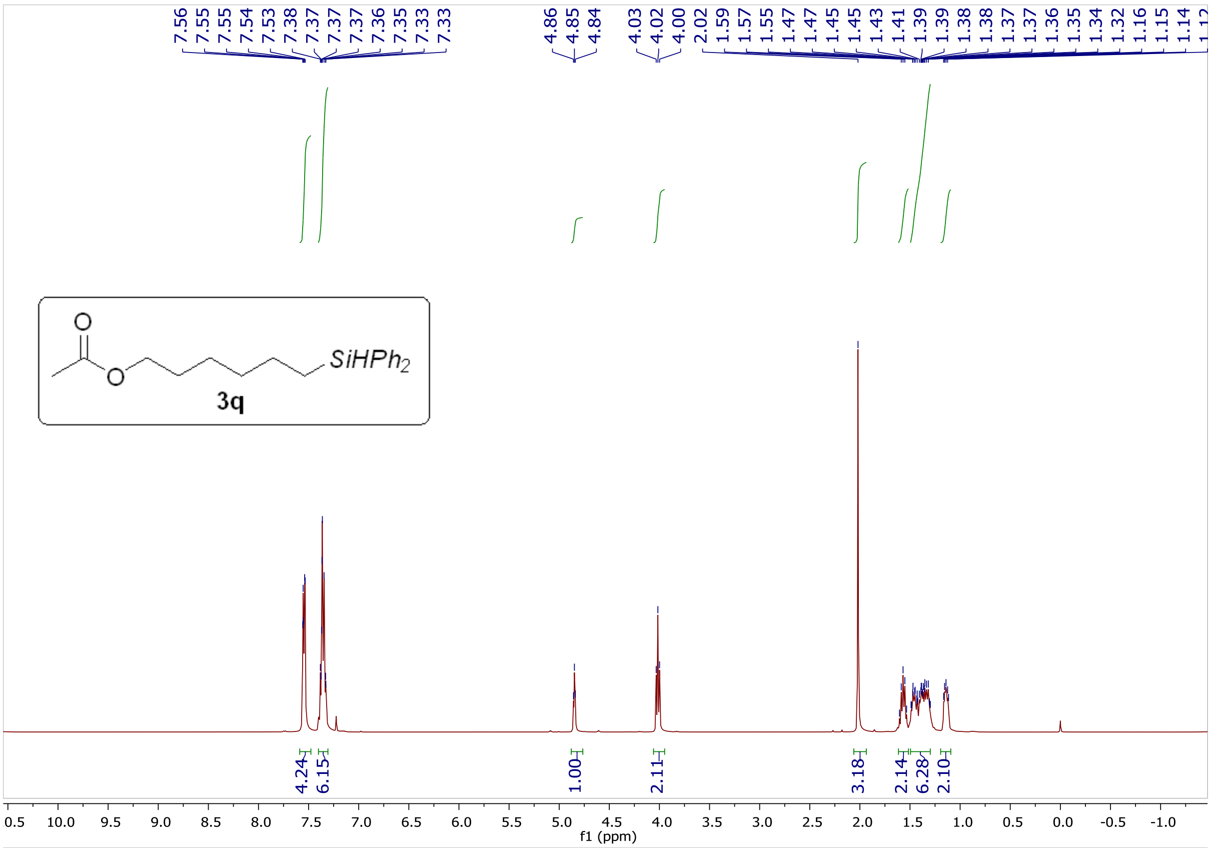
7.56
7.55
7.55
7.54
7.53
7.38
7.37
7.37
7.37
7.36
7.35
7.33
7.33
4.86
4.85
4.84
4.03
4.02
4.00
2.02
1.59
1.57
1.55
1.55
1.47
1.45
1.45
1.43
1.41
1.39
1.39
1.38
1.38
1.37
1.37
1.36
1.35
1.34
1.32
1.16
1.15
1.14
SiHPh2
3q
4.24
6.15
1.00
2.11
3.18
2.14
6.28
2.10
f1 (ppm)

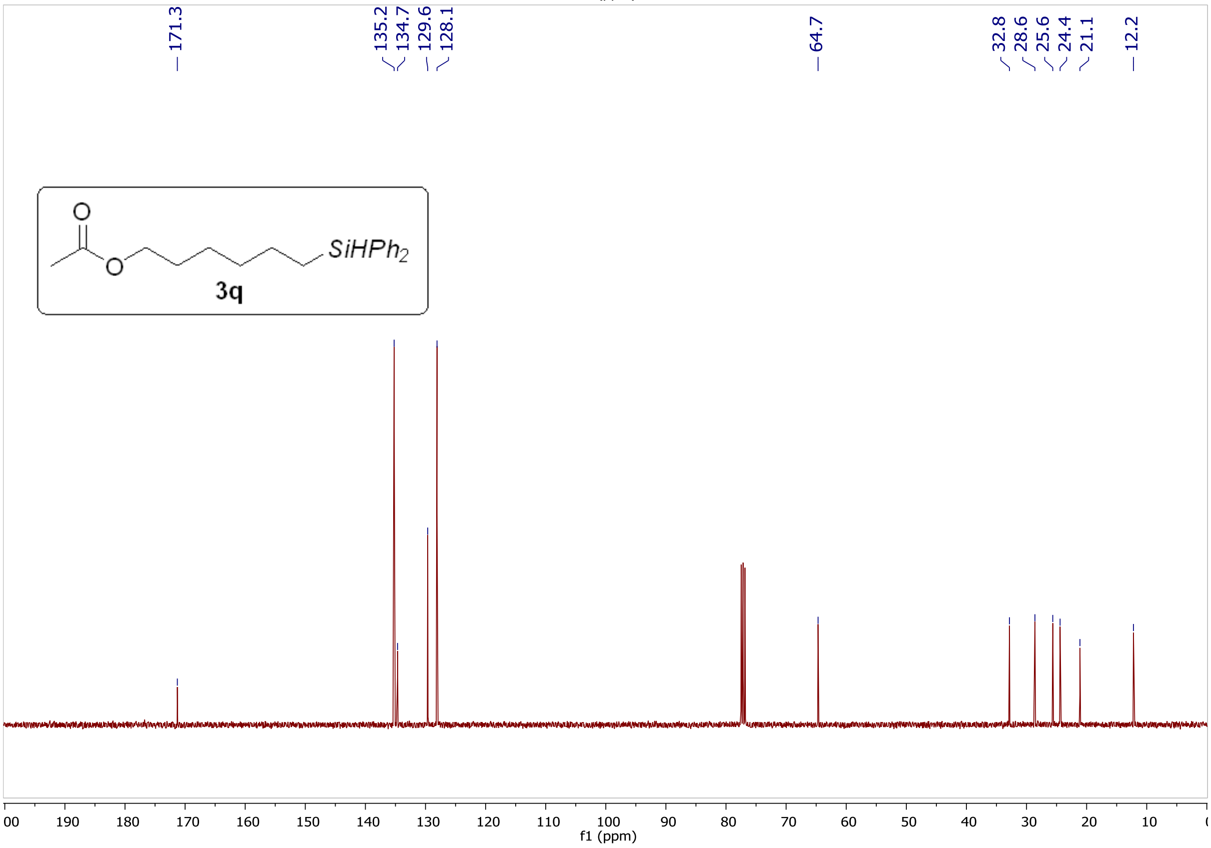
171.3
135.2
134.7
129.6
128.1
64.7
32.8
28.6
25.6
24.4
21.1
12.2
SiHPh2
3q
f1 (ppm)

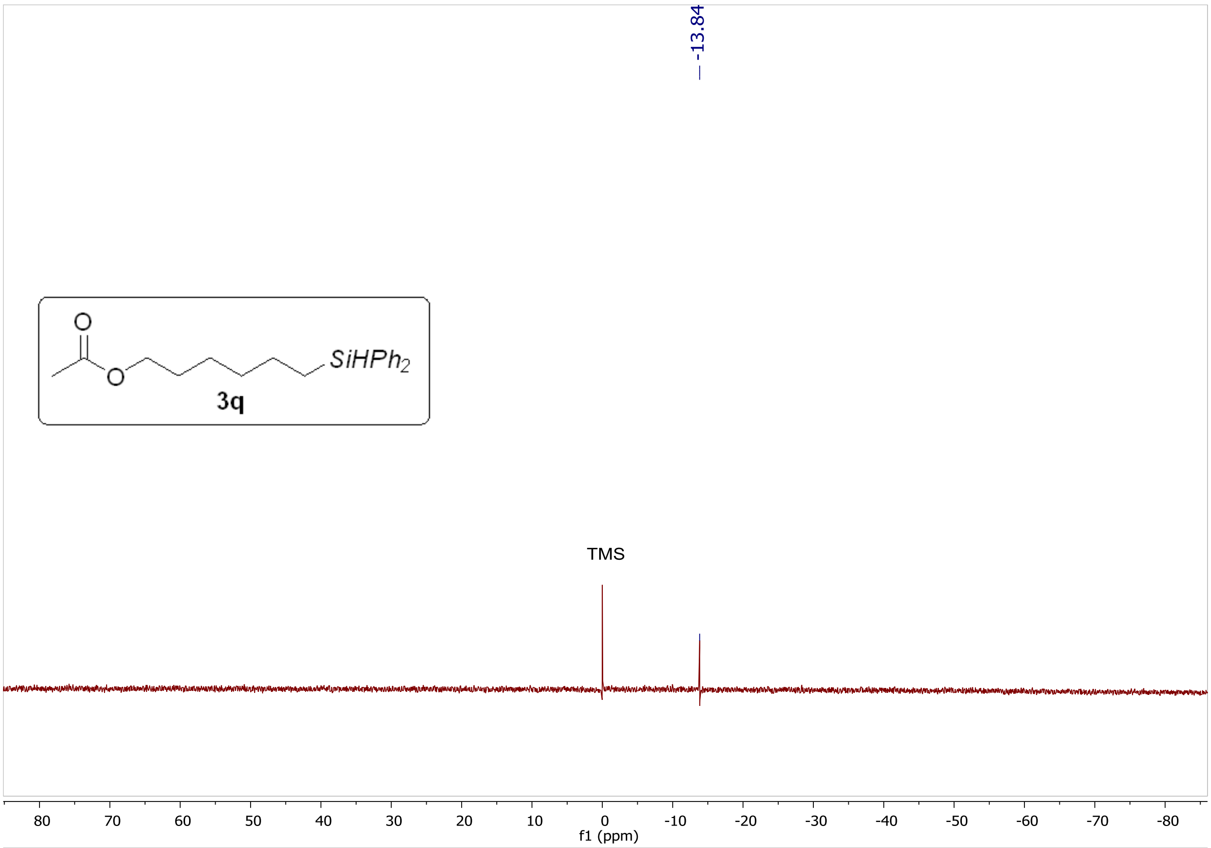
-13.84
SiHPh2
3q
TMS
f1 (ppm)

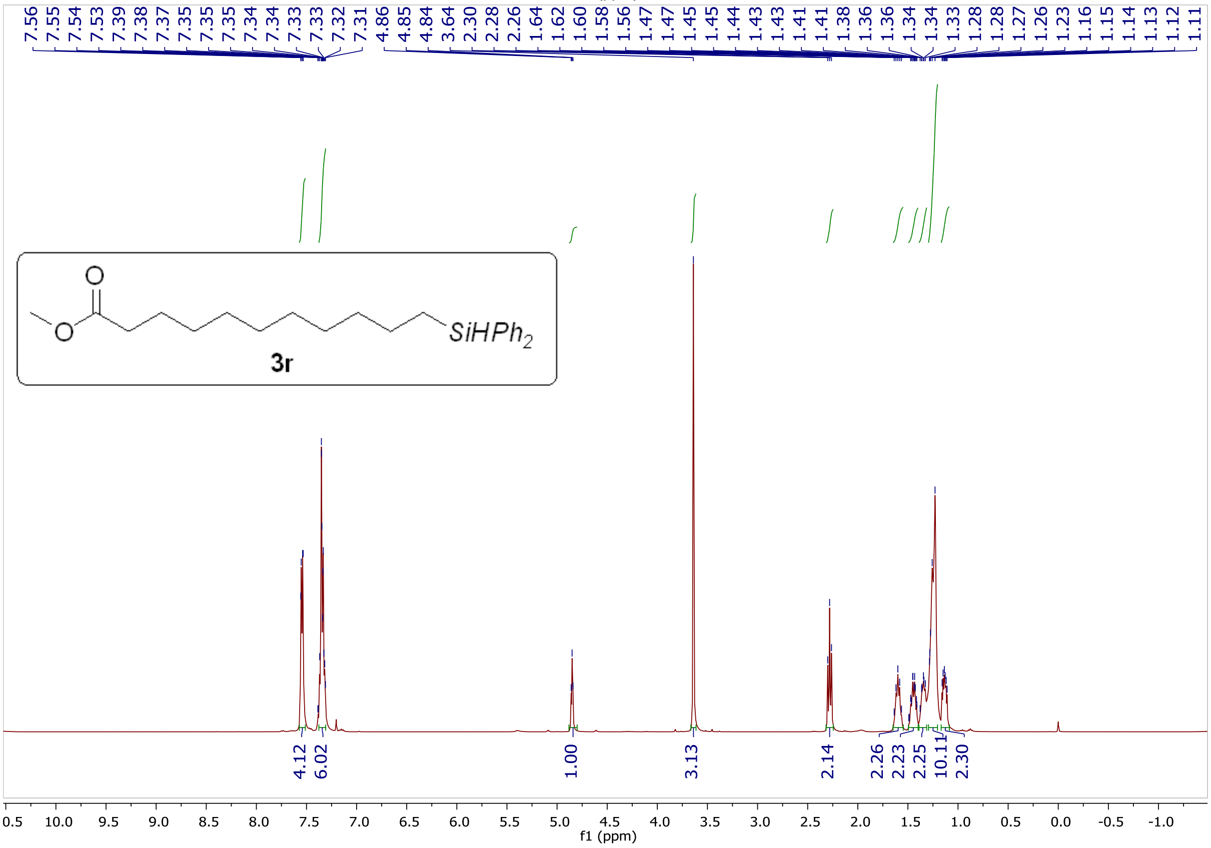
SiHPh2
3r
4.12
6.02
1.00
3.13
2.14
2.26
2.23
2.25
10.11
2.30
f1 (ppm)

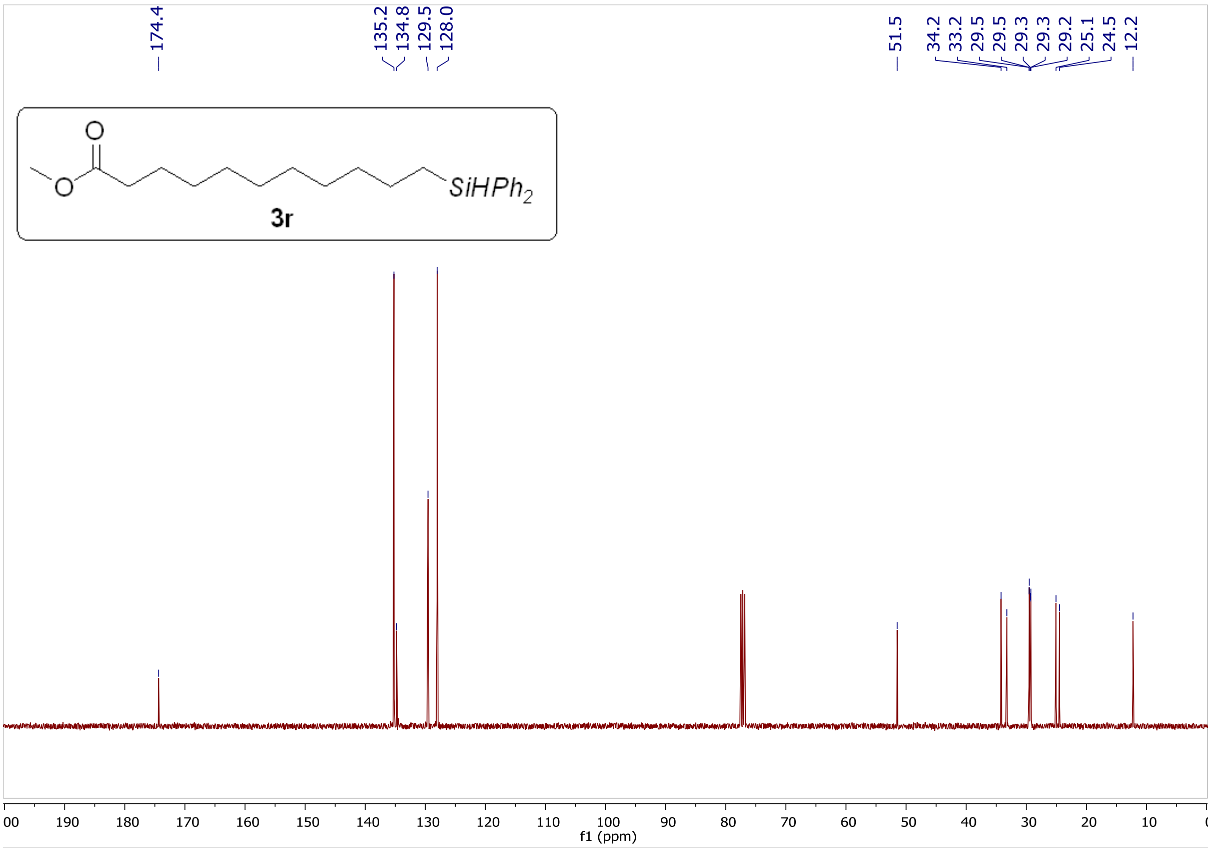
174.4
135.2
134.8
129.5
128.0
51.5
34.2
33.2
29.5
29.5
29.3
29.3
29.2
25.1
24.5
12.2
O
O
SiHPh2
3r
f1 (ppm)

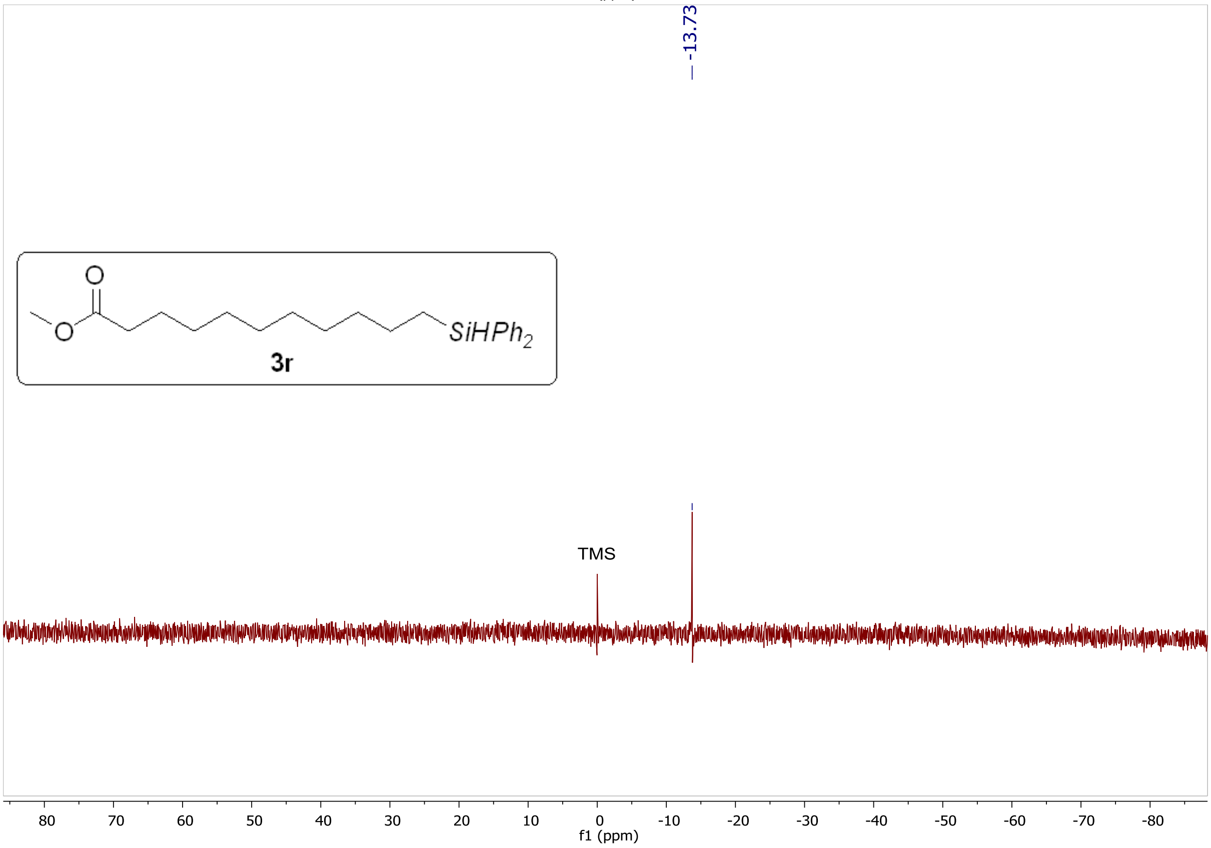
-13.73
O
O
SiHPh2
3r
TMS
f1 (ppm)

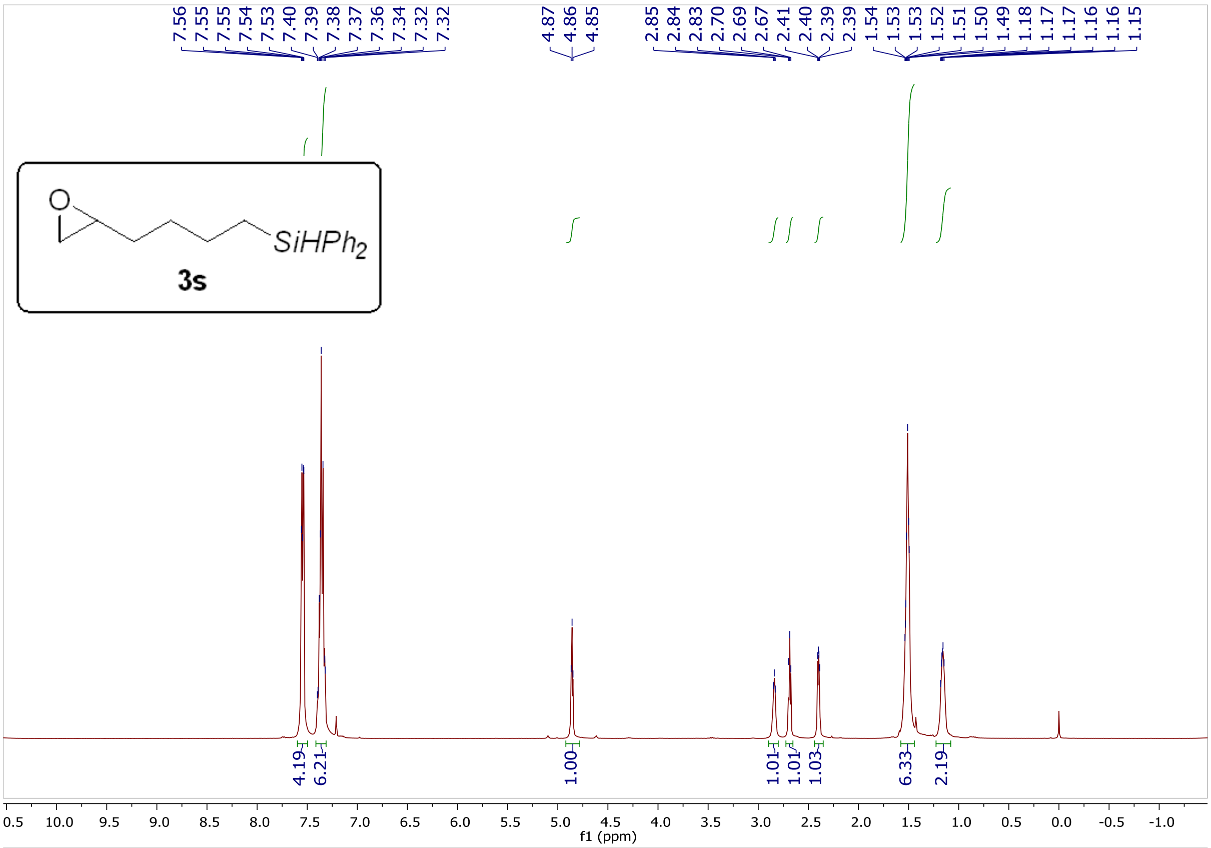
7.56 7.55 7.55 7.54 7.53 7.40 7.39 7.38 7.37 7.36 7.34 7.32 7.32
4.87 4.86 4.85
2.85 2.84 2.83 2.70 2.69 2.67 2.41 2.40 2.39 2.39 1.54 1.53 1.53 1.52 1.51 1.50 1.49 1.18 1.17 1.17 1.16 1.16 1.15
SiHPh2
3s
4.19 6.21 1.00 1.01 1.01 1.03 6.33 2.19
f1 (ppm)

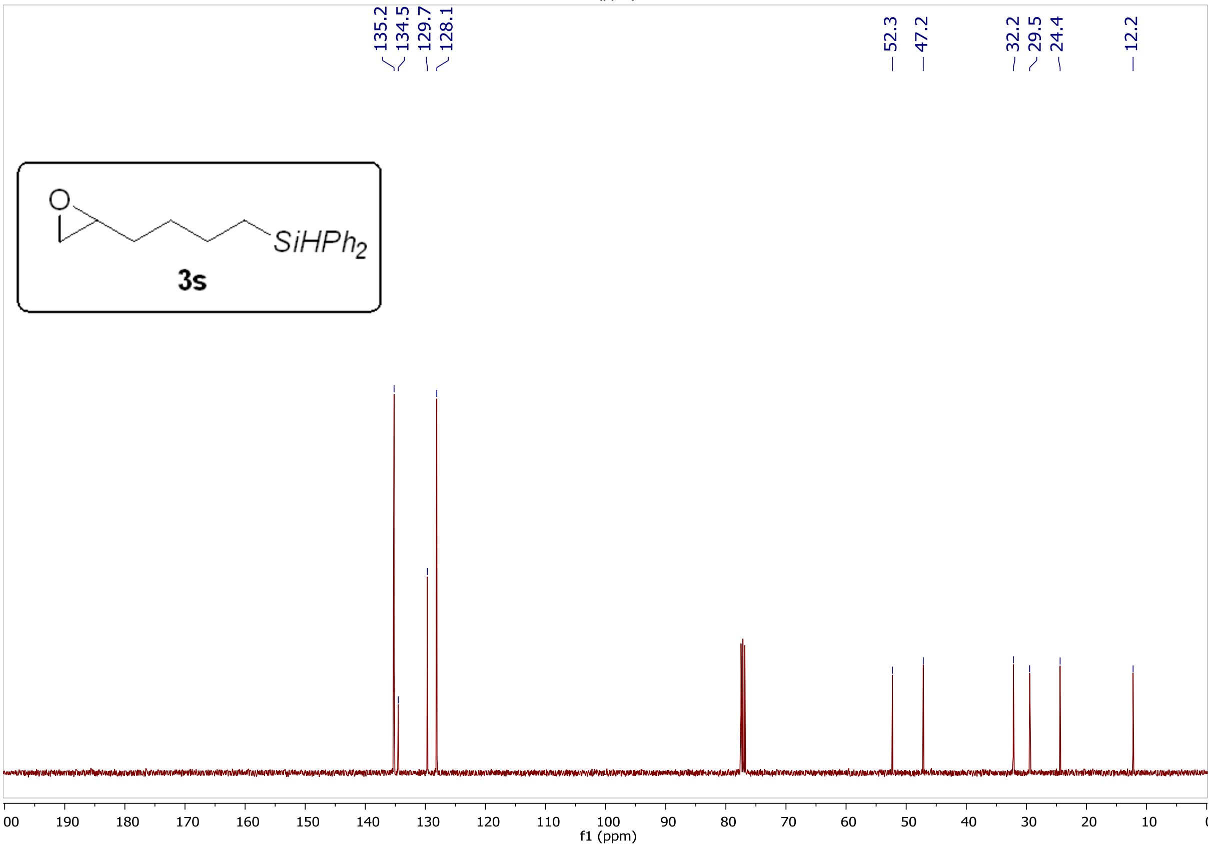
135.2 134.5 129.7 128.1
52.3 47.2
32.2 29.5 24.4
12.2
SiHPh2
3s
f1 (ppm)

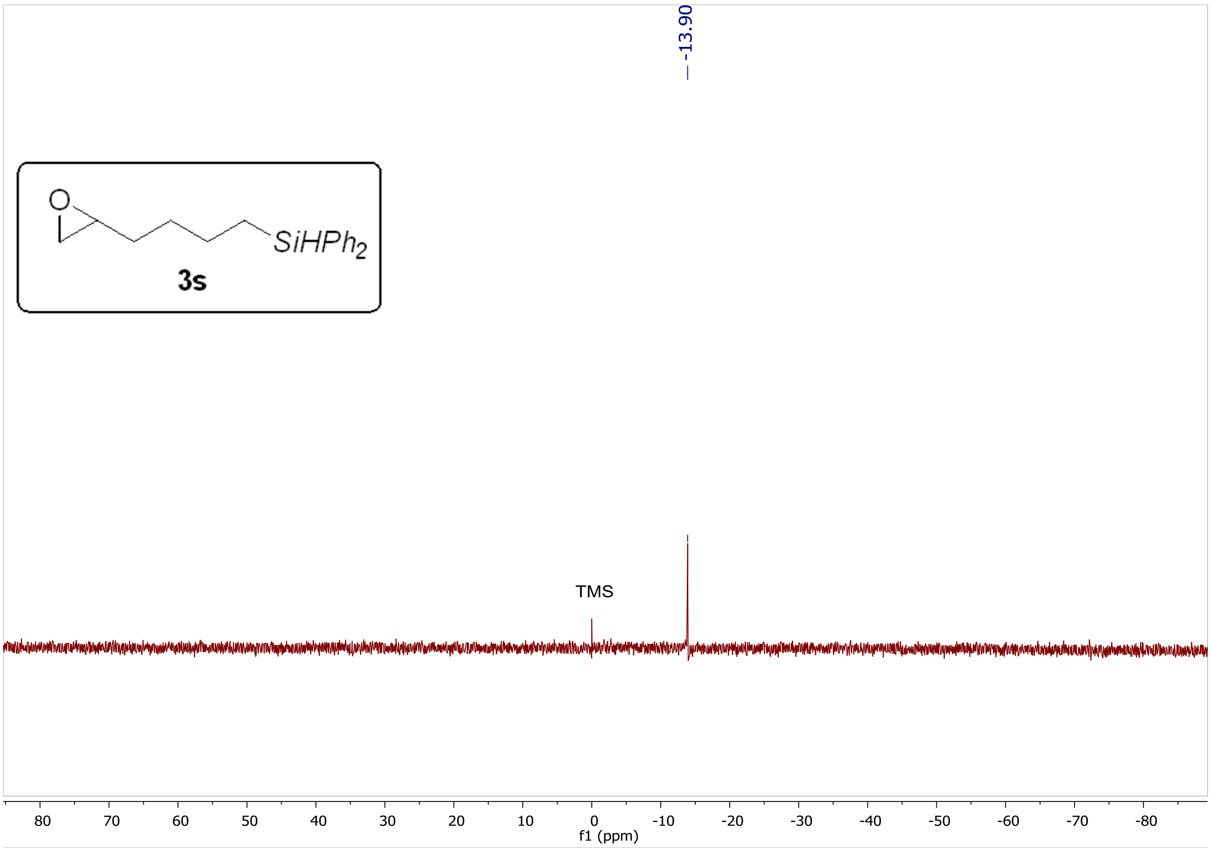
-13.90
SiHPh2
3s
TMS
f1 (ppm)

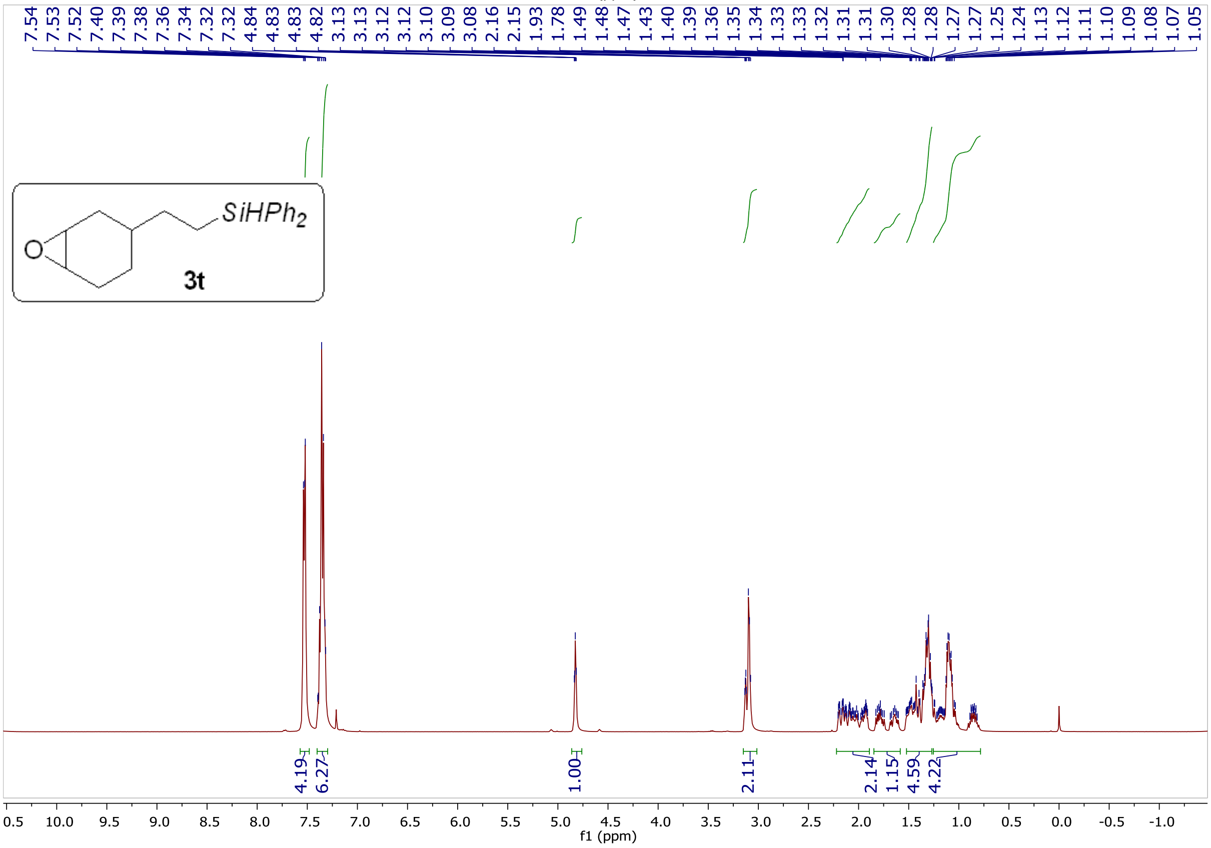
7.54 7.53 7.52 7.40 7.39 7.38 7.36 7.34 7.32 7.32 4.84 4.83 4.83 4.82 3.13 3.13 3.12 3.12 3.10 3.09 3.08 2.16 2.15 1.93 1.78 1.49 1.48 1.47 1.43 1.40 1.39 1.36 1.35 1.34 1.33 1.33 1.32 1.31 1.31 1.30 1.28 1.28 1.27 1.27 1.25 1.24 1.13 1.12 1.11 1.10 1.09 1.08 1.07 1.05
SiHPh2
3t
4.19
6.27
1.00
2.11
2.14
1.15
4.59
4.22
f1 (ppm)

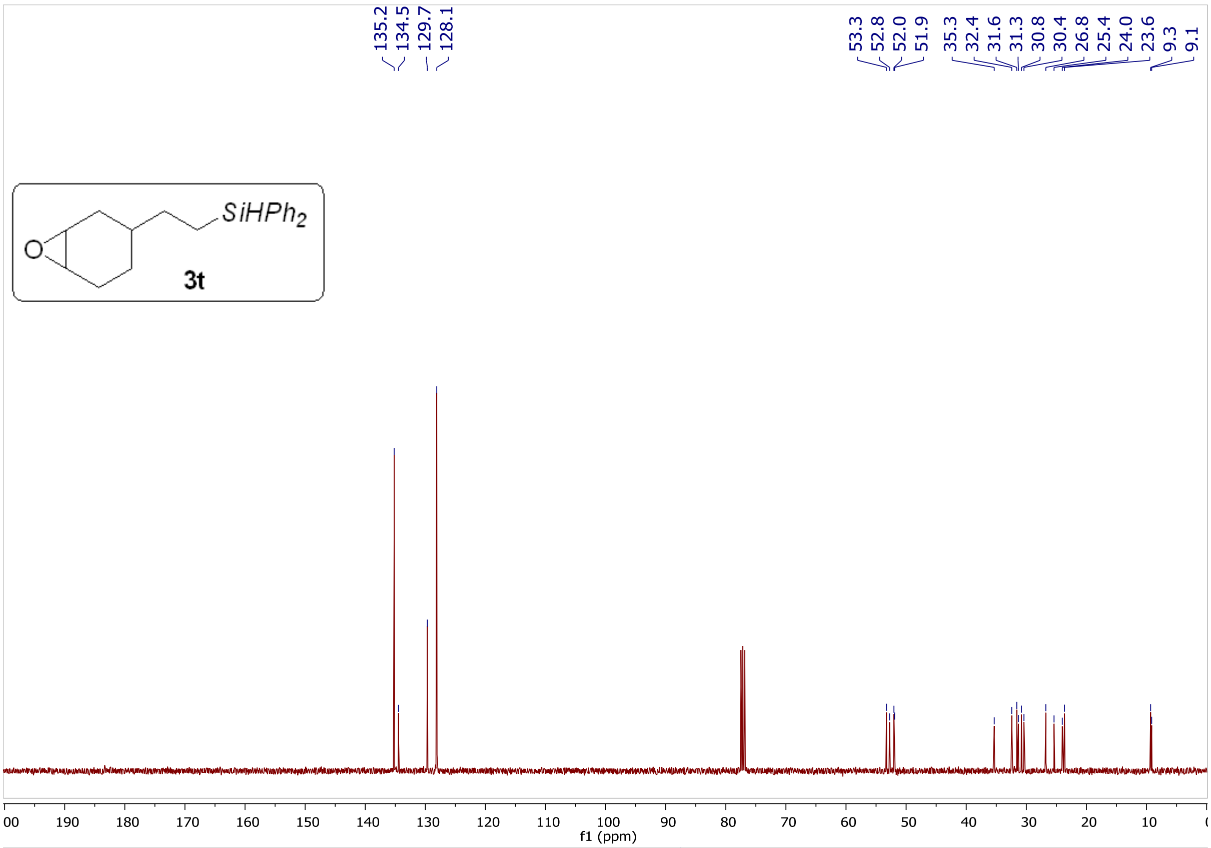
135.2
134.5
129.7
128.1
53.3
52.8
52.0
51.9
35.3
32.4
31.6
31.3
30.8
30.4
26.8
25.4
24.0
23.6
9.3
9.1
SiHPh2
O
3t
f1 (ppm)

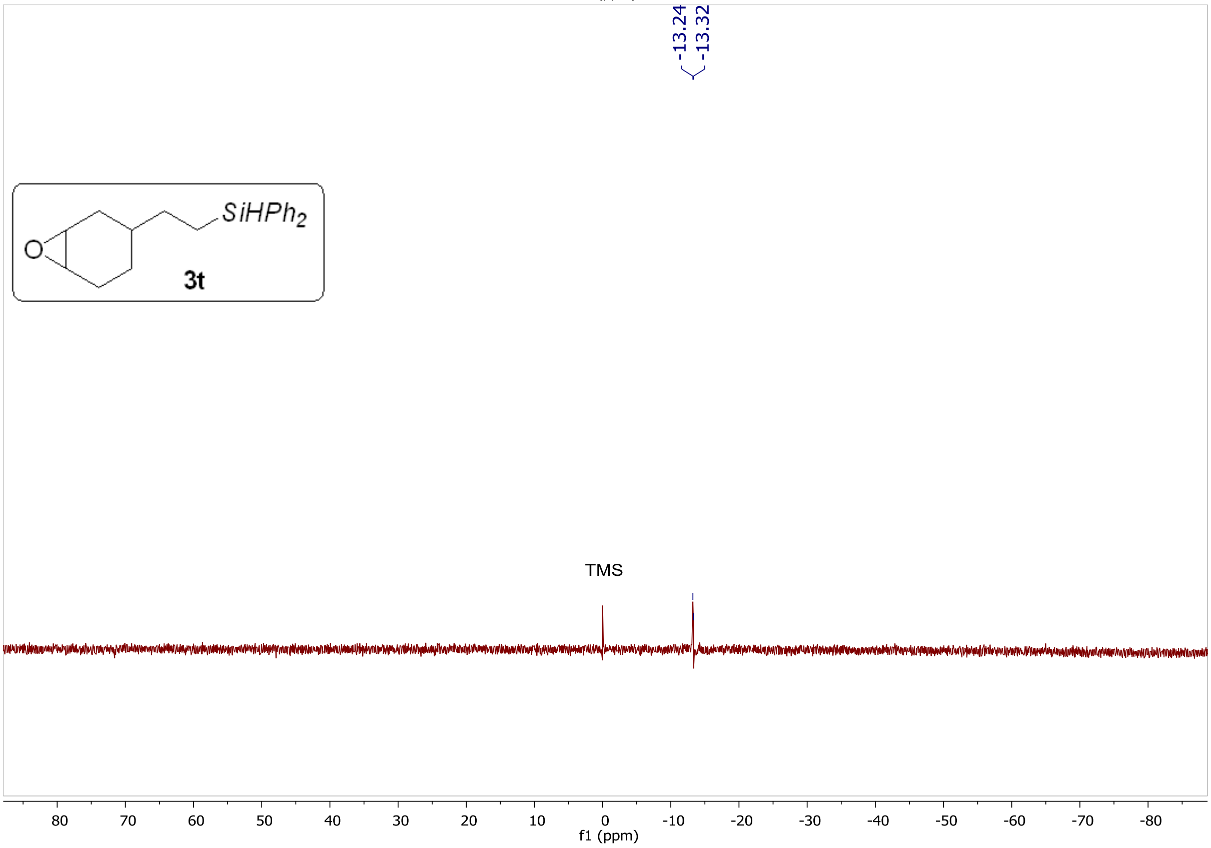
-13.24
-13.32
SiHPh2
O
3t
TMS
f1 (ppm)

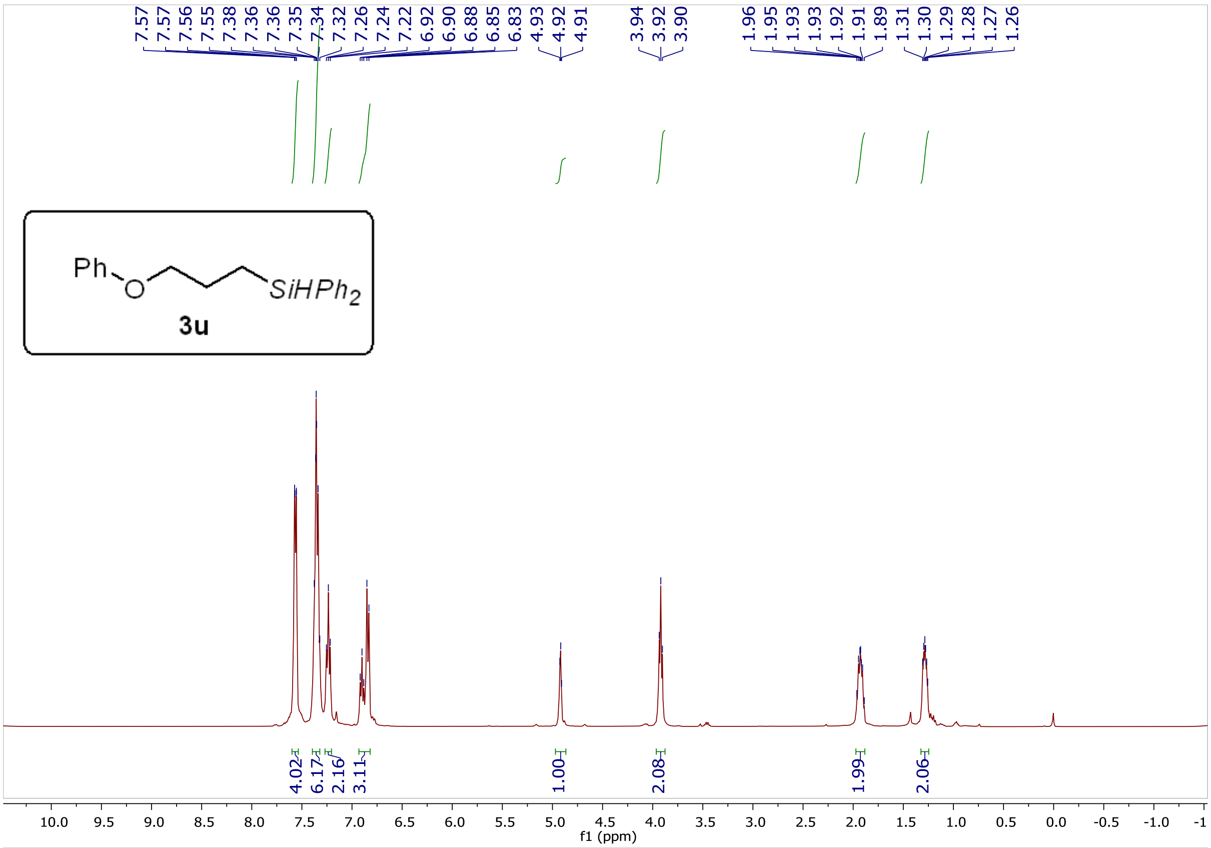
7.57 7.57 7.56 7.55 7.38 7.36 7.35 7.34 7.32 7.26 7.24 7.22 6.92 6.90 6.88 6.85 6.83 4.93 4.92 4.91 3.94 3.92 3.90 1.96 1.95 1.93 1.93 1.92 1.91 1.89 1.31 1.30 1.29 1.28 1.27 1.26
Ph
O
SiHPh2
3u
4.02 6.17 2.16 3.11 1.00 2.08 1.99 2.06
f1 (ppm)

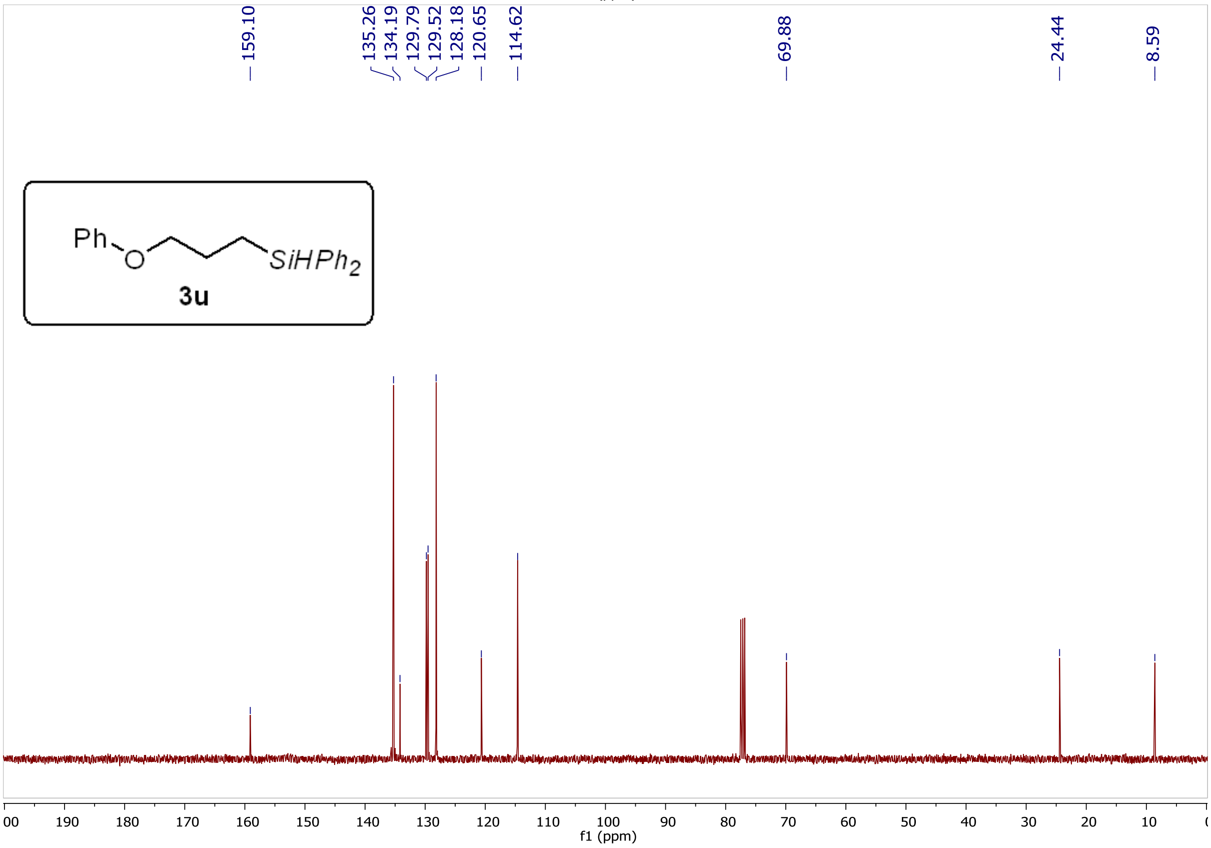
159.10 135.26 134.19 129.79 129.52 128.18 120.65 114.62 69.88 24.44 8.59
Ph
O
SiHPh2
3u
f1 (ppm)

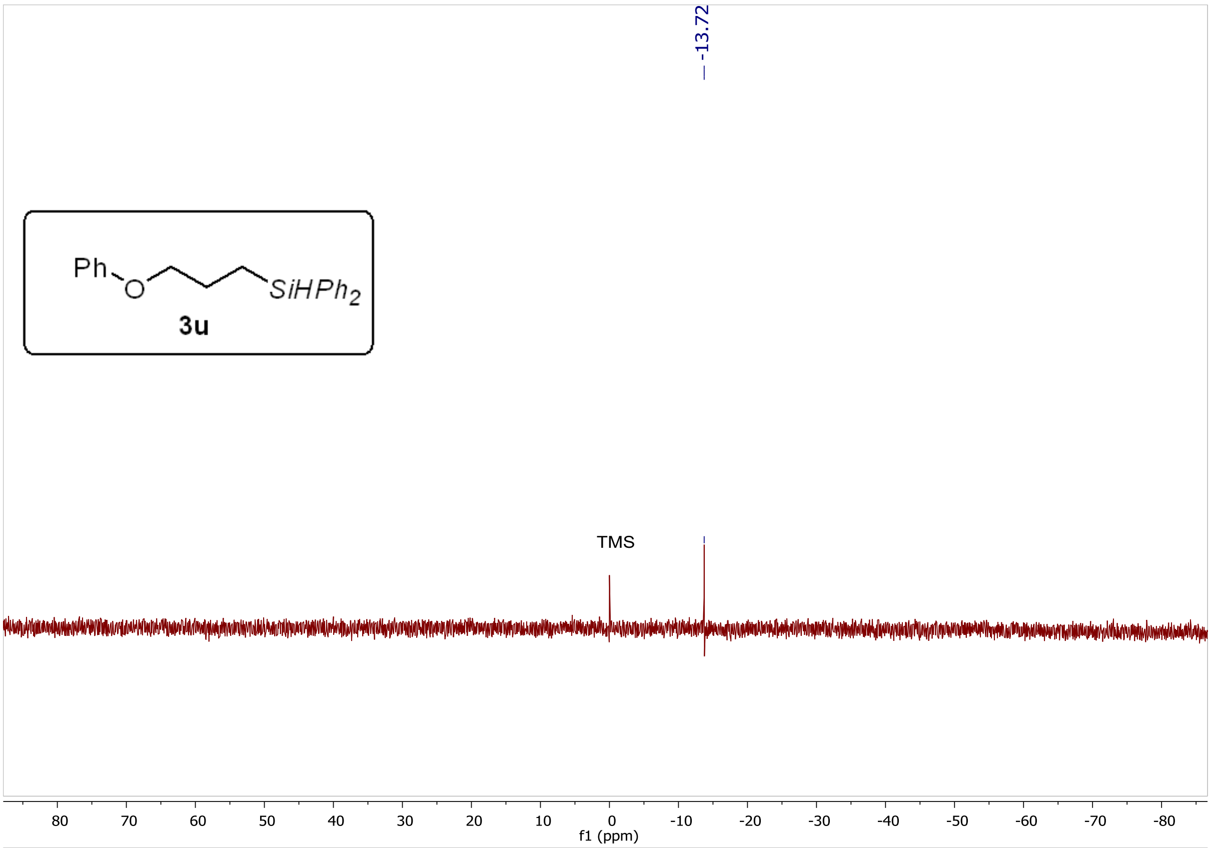
-13.72
Ph
O
SiHPh2
3u
TMS
f1 (ppm)

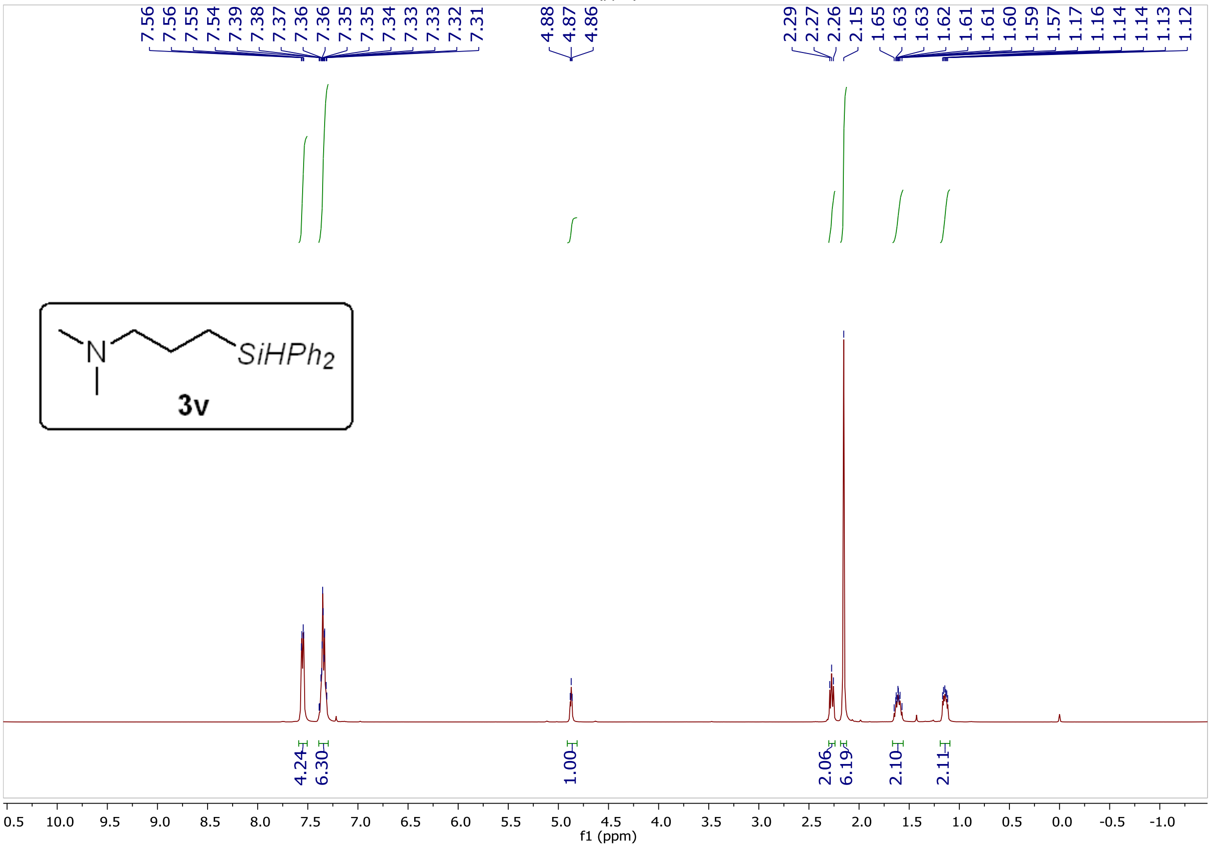
7.56
7.56
7.55
7.54
7.39
7.38
7.37
7.36
7.36
7.35
7.35
7.34
7.33
7.33
7.32
7.31
4.88
4.87
4.86
2.29
2.27
2.26
2.15
1.65
1.63
1.63
1.62
1.61
1.61
1.60
1.59
1.57
1.17
1.16
1.14
1.14
1.13
1.12
N
SiHPh2
3v
4.24
6.30
1.00
2.06
6.19
2.10
2.11
f1 (ppm)

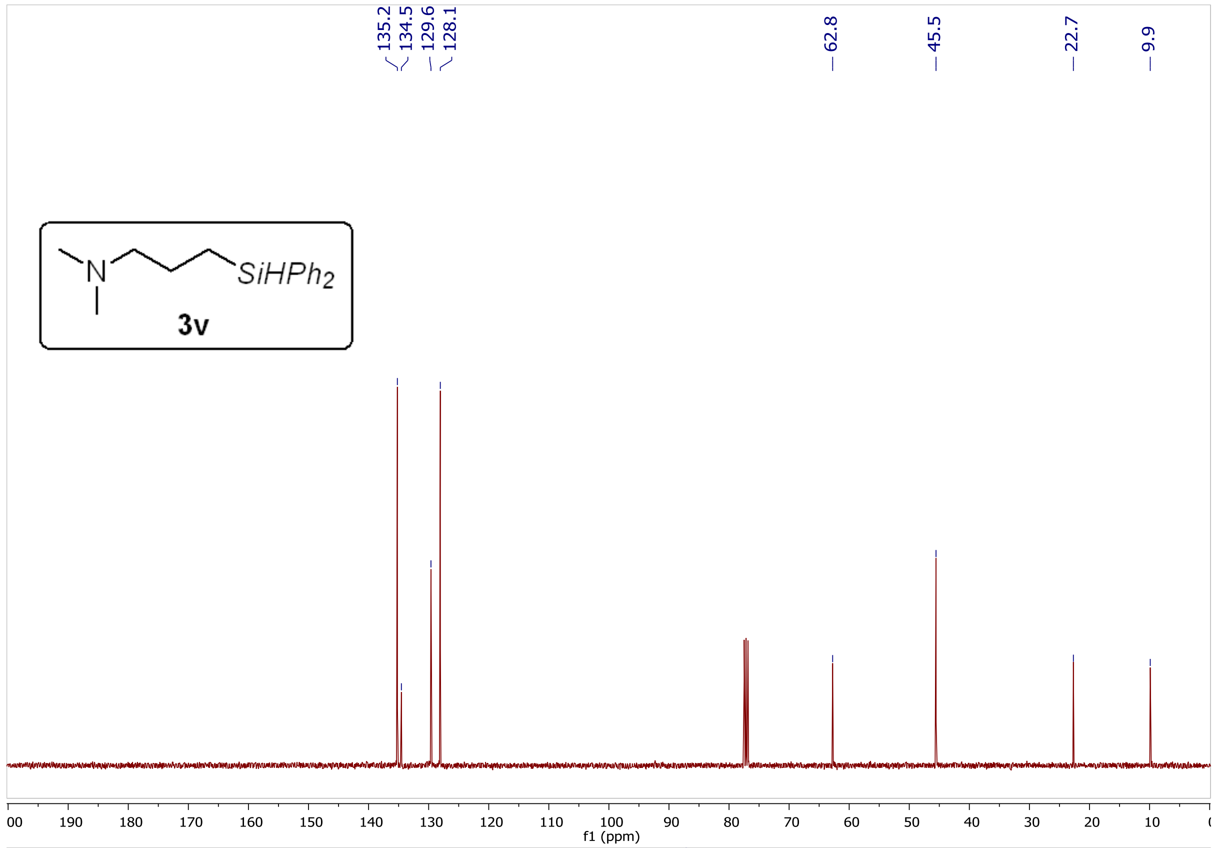
135.2
134.5
129.6
128.1
62.8
45.5
22.7
9.9
SiHPh2
3v
f1 (ppm)

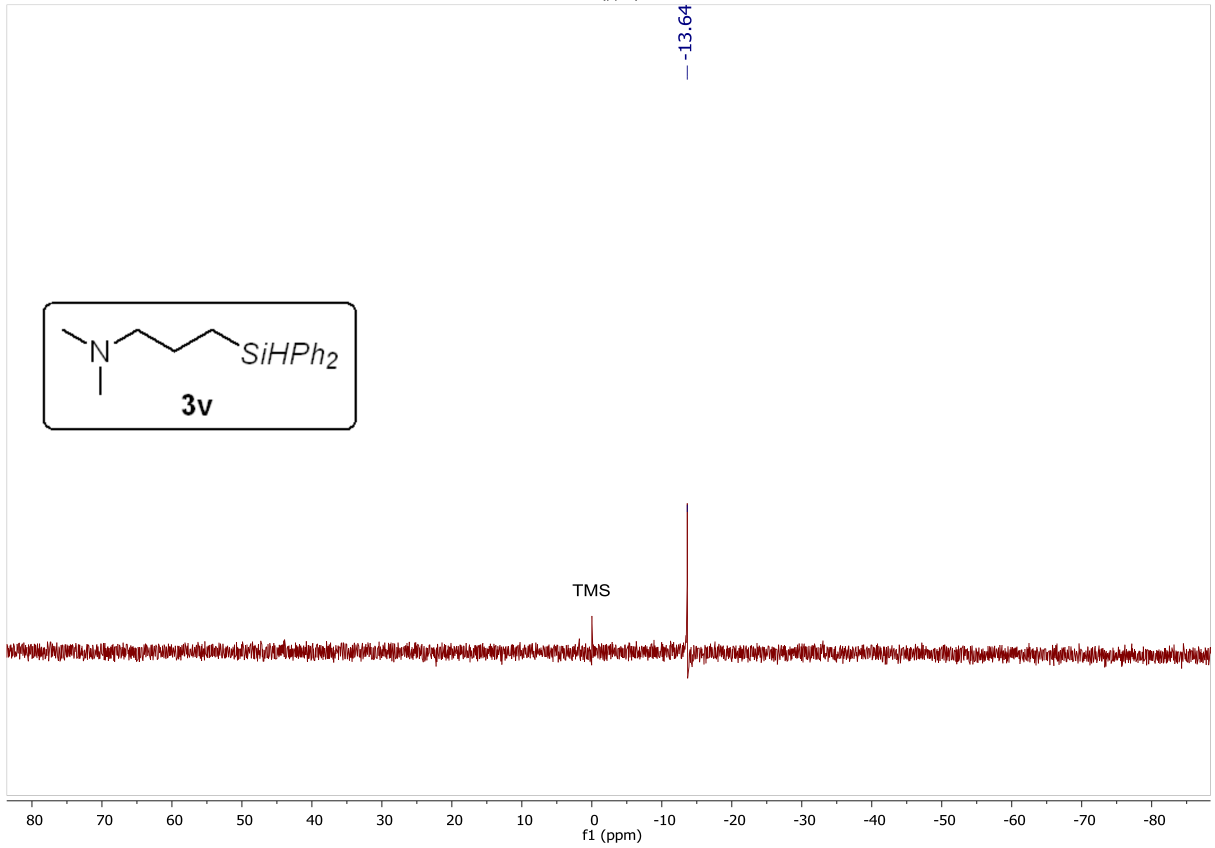
-13.64
SiHPh2
3v
TMS
f1 (ppm)

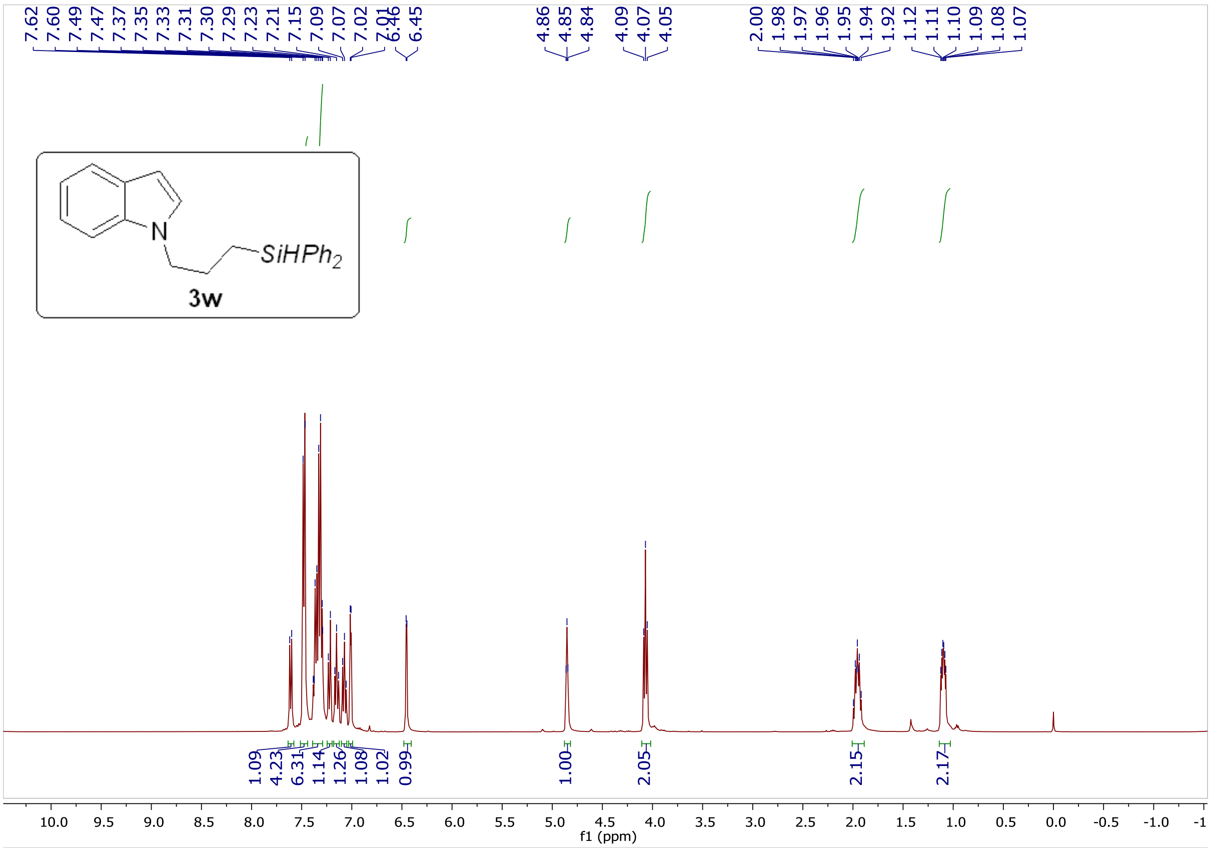
7.62
7.60
7.49
7.47
7.37
7.35
7.33
7.31
7.30
7.29
7.23
7.21
7.15
7.09
7.07
7.02
7.01
6.46
6.45
4.86
4.85
4.84
4.09
4.07
4.05
2.00
1.98
1.97
1.96
1.95
1.94
1.92
1.12
1.11
1.10
1.09
1.08
1.07
N
SiHPh2
3w
1.09
4.23
6.31
1.14
1.26
1.08
1.02
0.99
1.00
2.05
2.15
2.17
f1 (ppm)

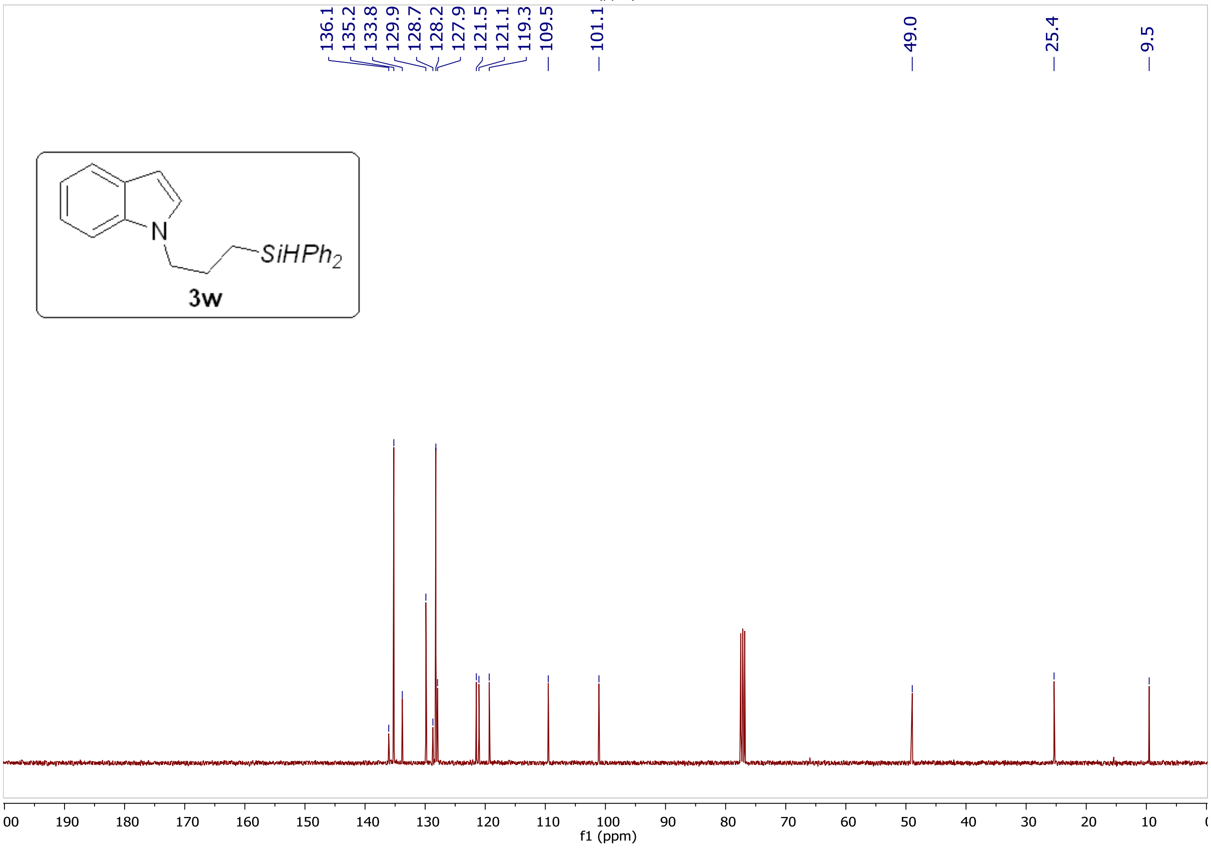
136.1
135.2
133.8
129.9
128.7
128.2
127.9
121.5
121.1
119.3
109.5
101.1
49.0
25.4
9.5
N
SiHPh2
3w
f1 (ppm)

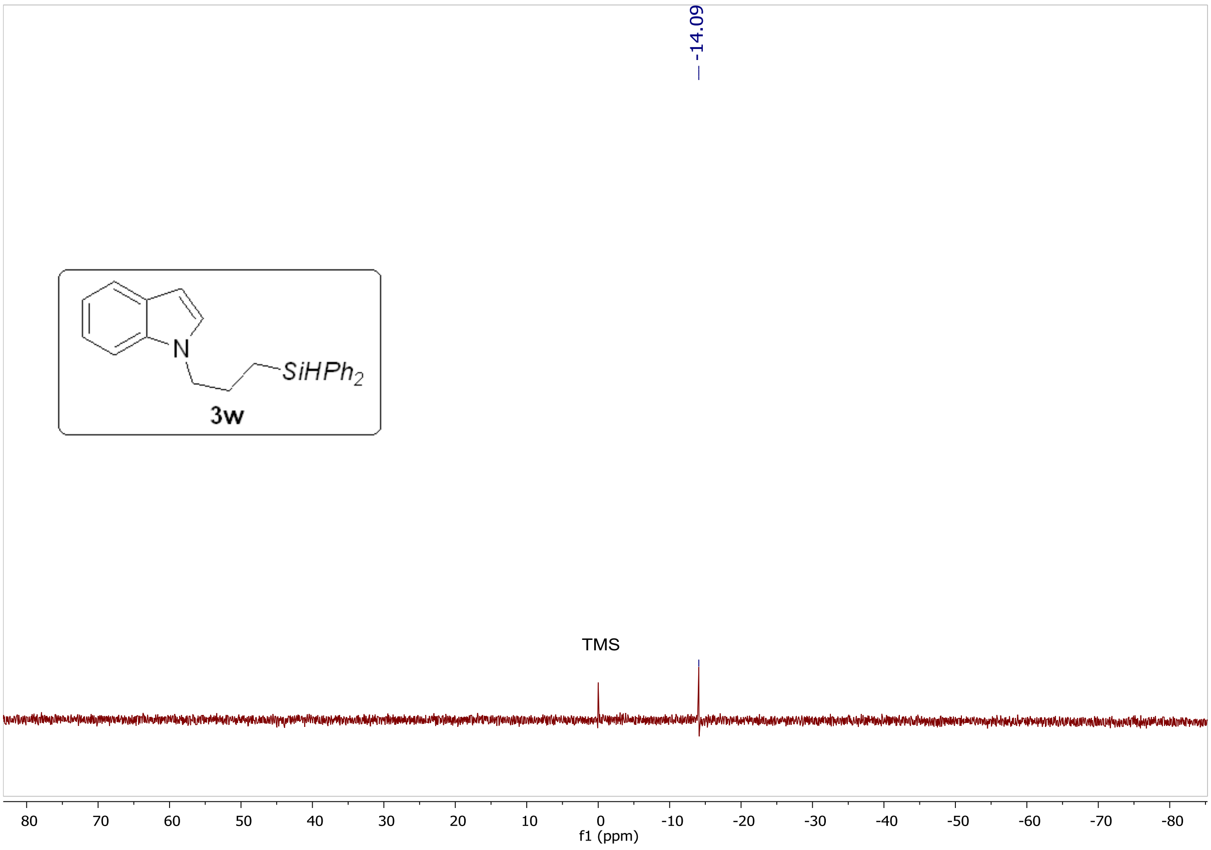
-14.09
TMS
N
SiHPh2
3w
80 70 60 50 40 30 20 10 0 -10 -20 -30 -40 -50 -60 -70 -80
f1 (ppm)

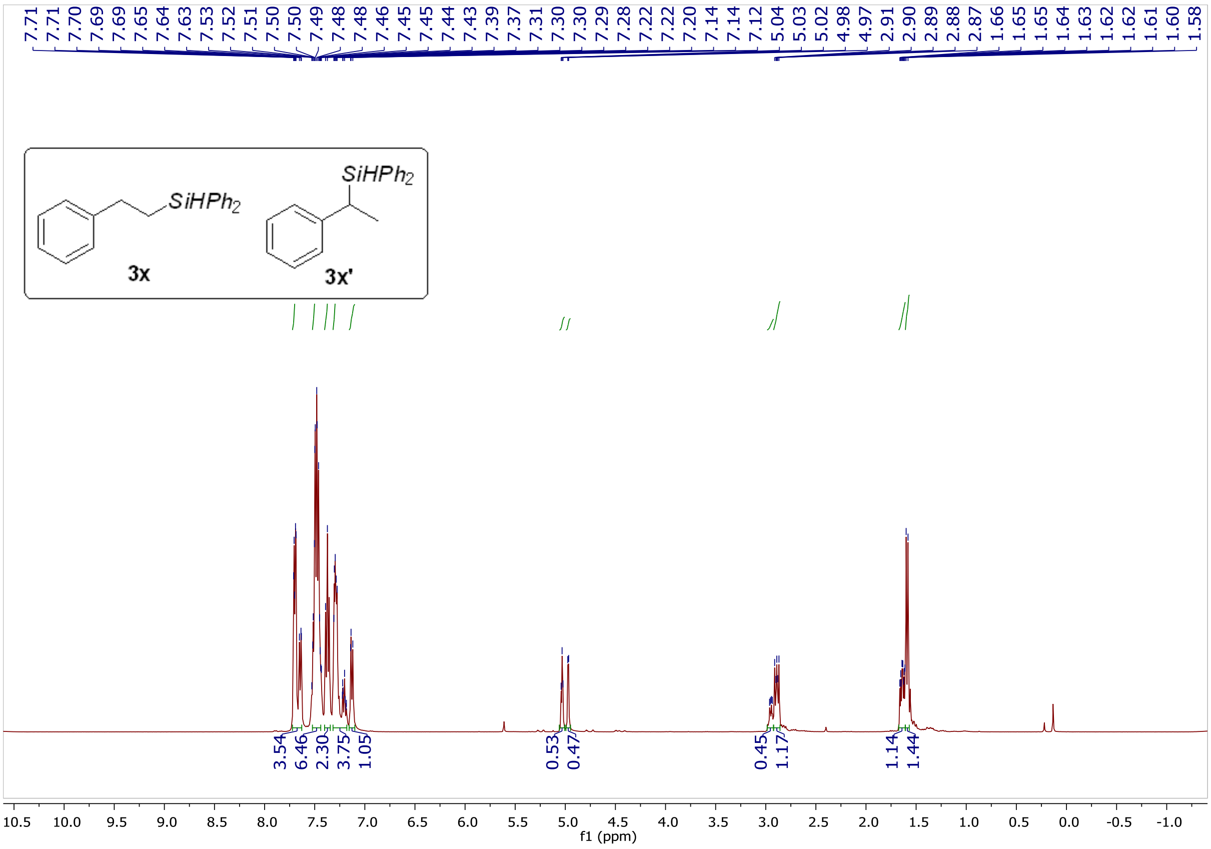
7.71 7.71 7.70 7.69 7.69 7.65 7.64 7.63 7.53 7.52 7.51 7.50 7.49 7.48 7.48 7.46 7.45 7.45 7.44 7.43 7.39 7.37 7.31 7.30 7.30 7.29 7.28 7.22 7.22 7.20 7.14 7.14 7.12 5.04 5.03 5.02 4.98 4.97 2.91 2.90 2.89 2.88 2.87 1.66 1.65 1.65 1.64 1.63 1.62 1.62 1.61 1.60 1.58
SiHPh2
SiHPh2
3x
3x'
3.54 6.46 2.30 3.75 1.05
0.53 0.47
0.45 1.17
1.14 1.44
10.5 10.0 9.5 9.0 8.5 8.0 7.5 7.0 6.5 6.0 5.5 5.0 4.5 4.0 3.5 3.0 2.5 2.0 1.5 1.0 0.5 0.0 -0.5 -1.0
f1 (ppm)

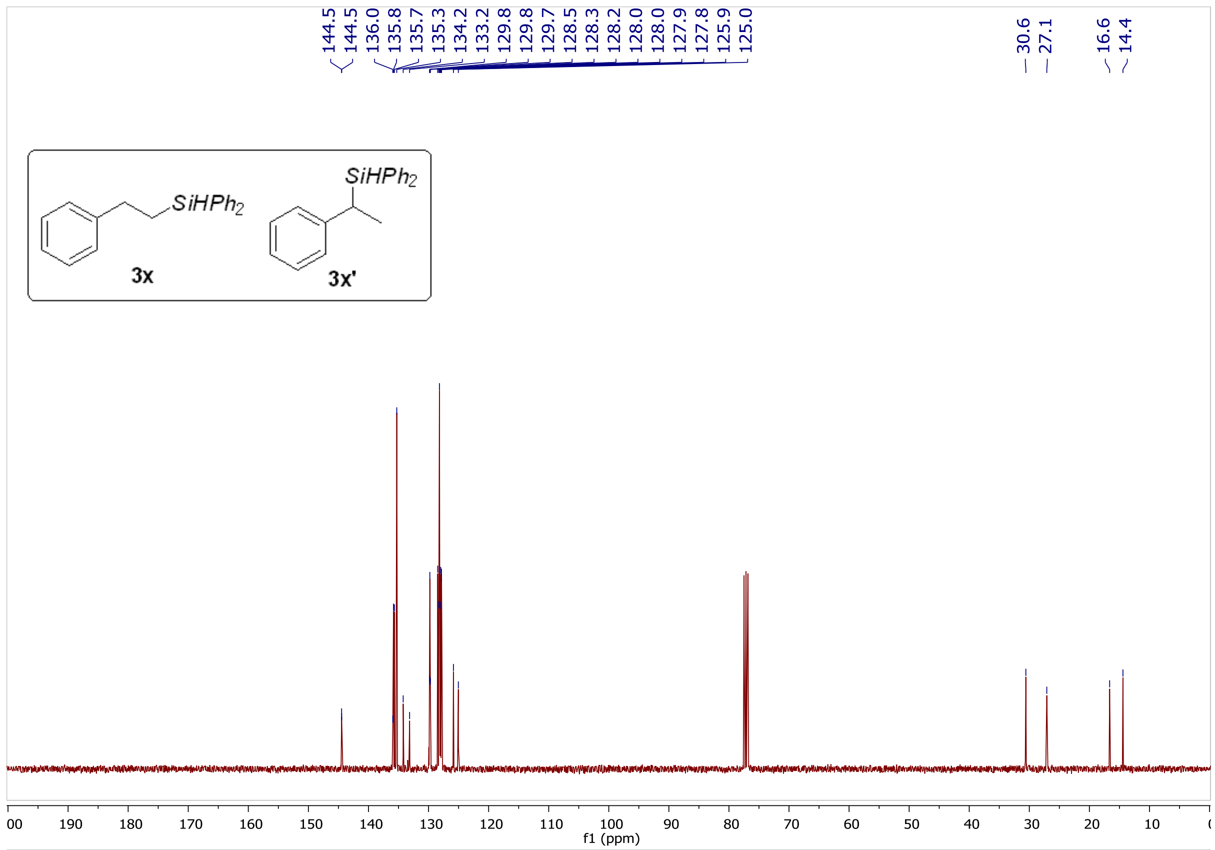
144.5
144.5
136.0
135.8
135.7
135.3
134.2
133.2
129.8
129.8
129.7
128.5
128.3
128.2
128.0
128.0
127.9
127.8
125.9
125.0
30.6
27.1
16.6
14.4
SiHPh2
SiHPh2
3x
3x'
f1 (ppm)

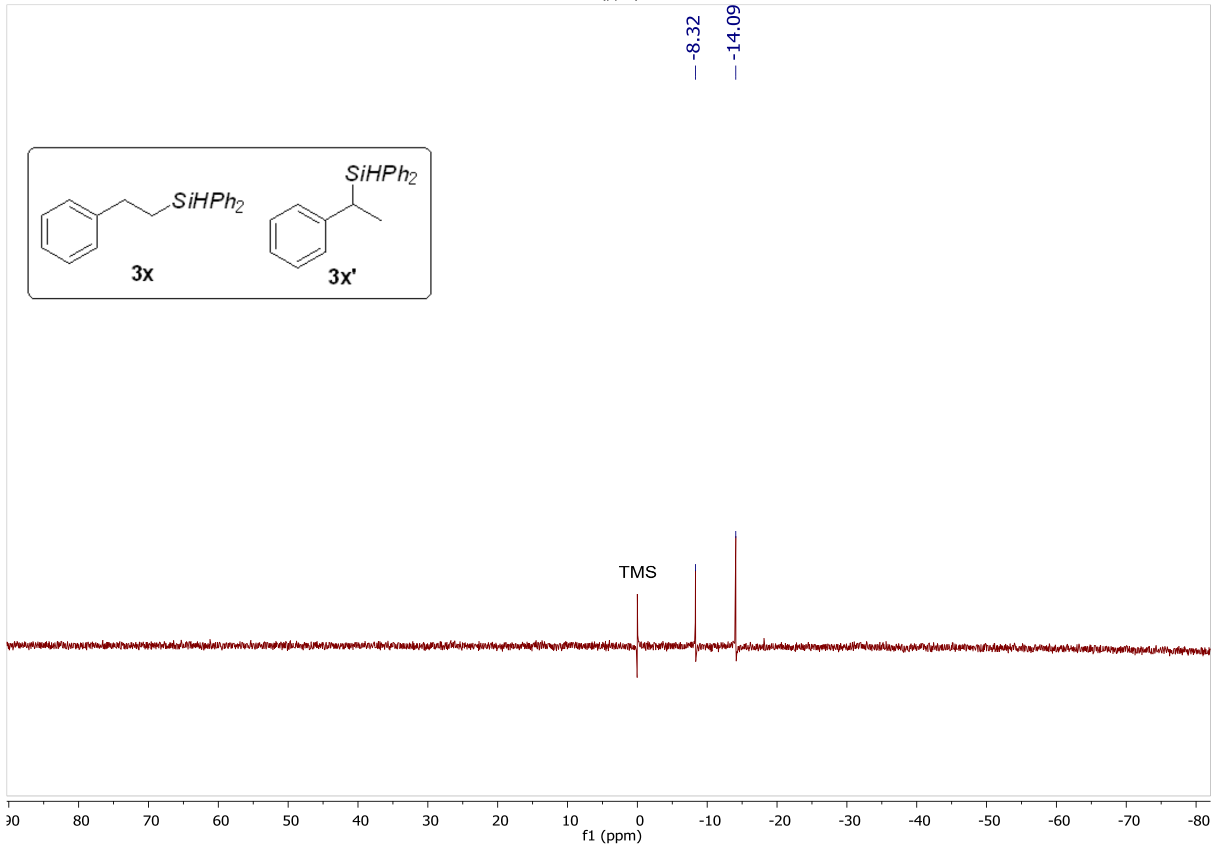
-8.32
-14.09
SiHPh2
SiHPh2
3x
3x'
TMS
f1 (ppm)

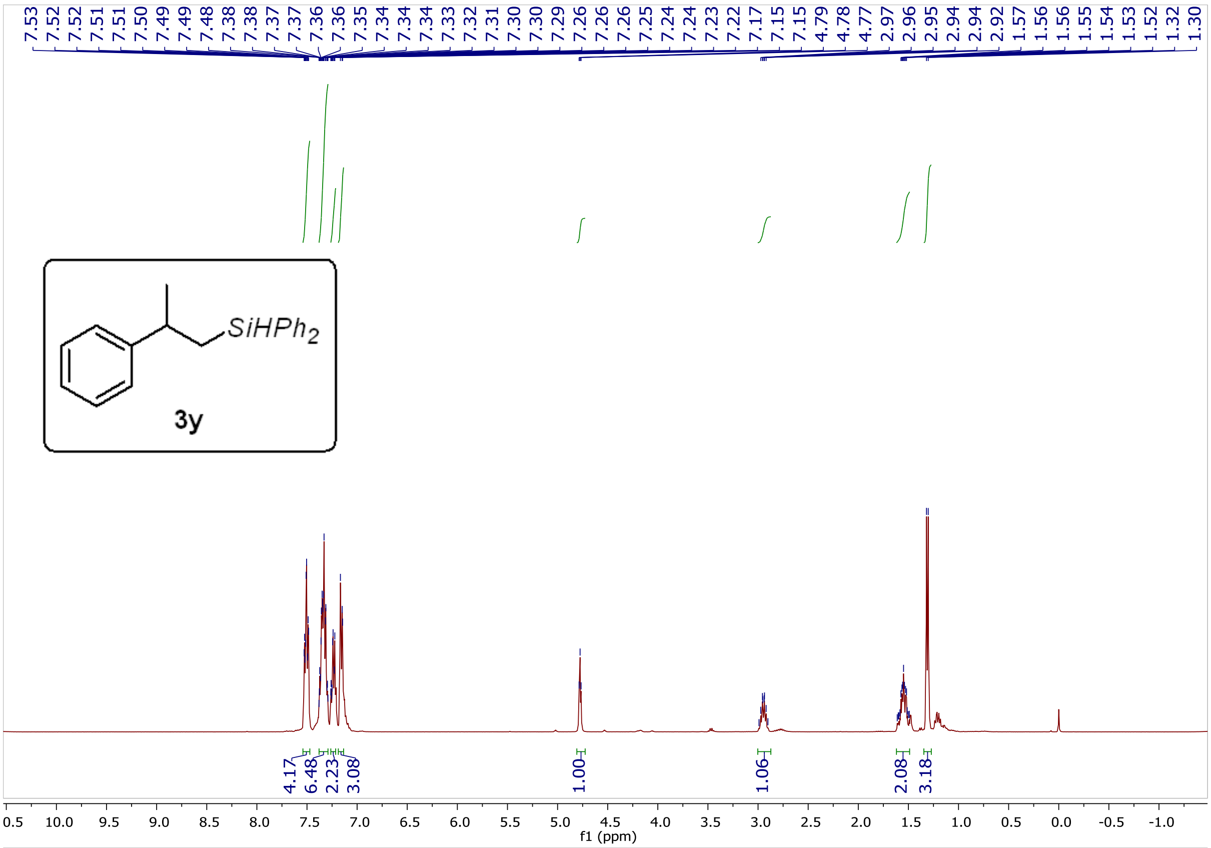
SiHPh2
3y
f1 (ppm)

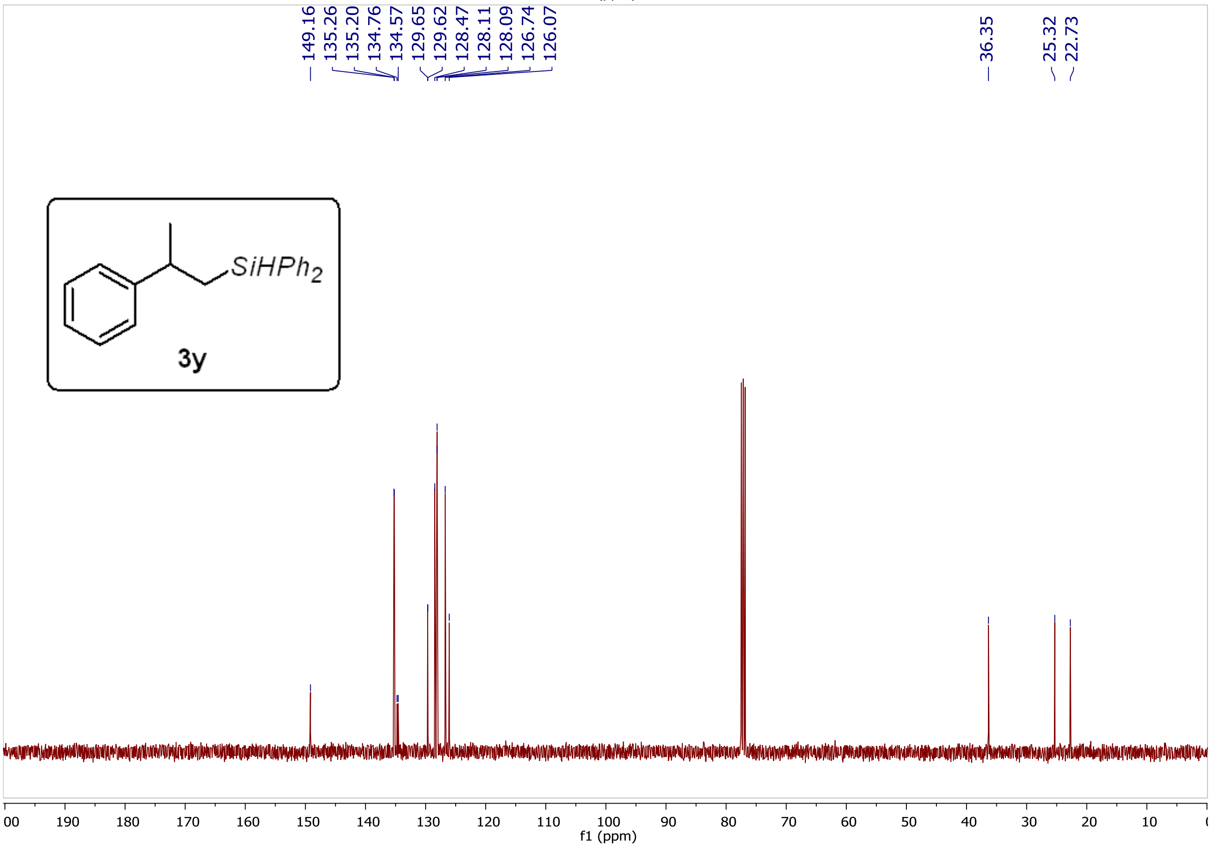
SiHPh2
3y
f1 (ppm)

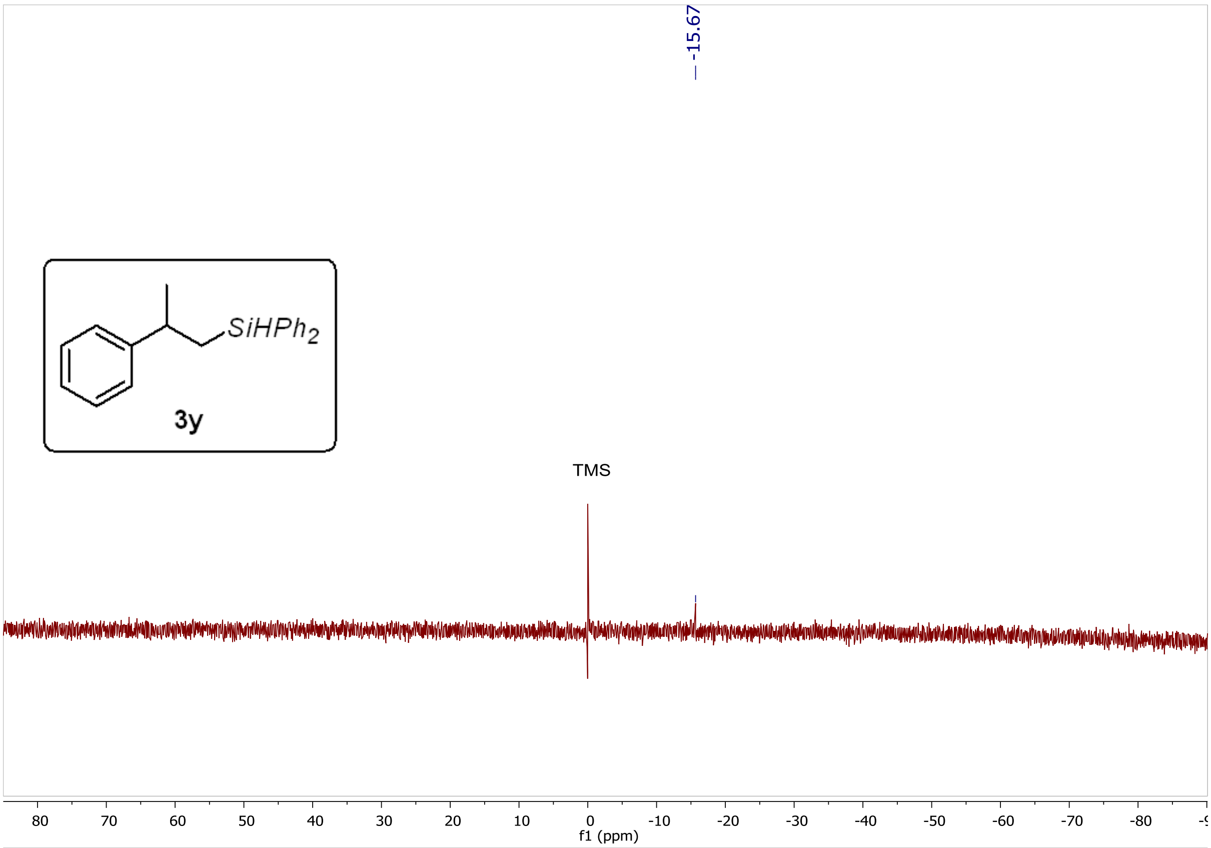

-15.67
SiHPh2
3y
TMS
f1 (ppm)

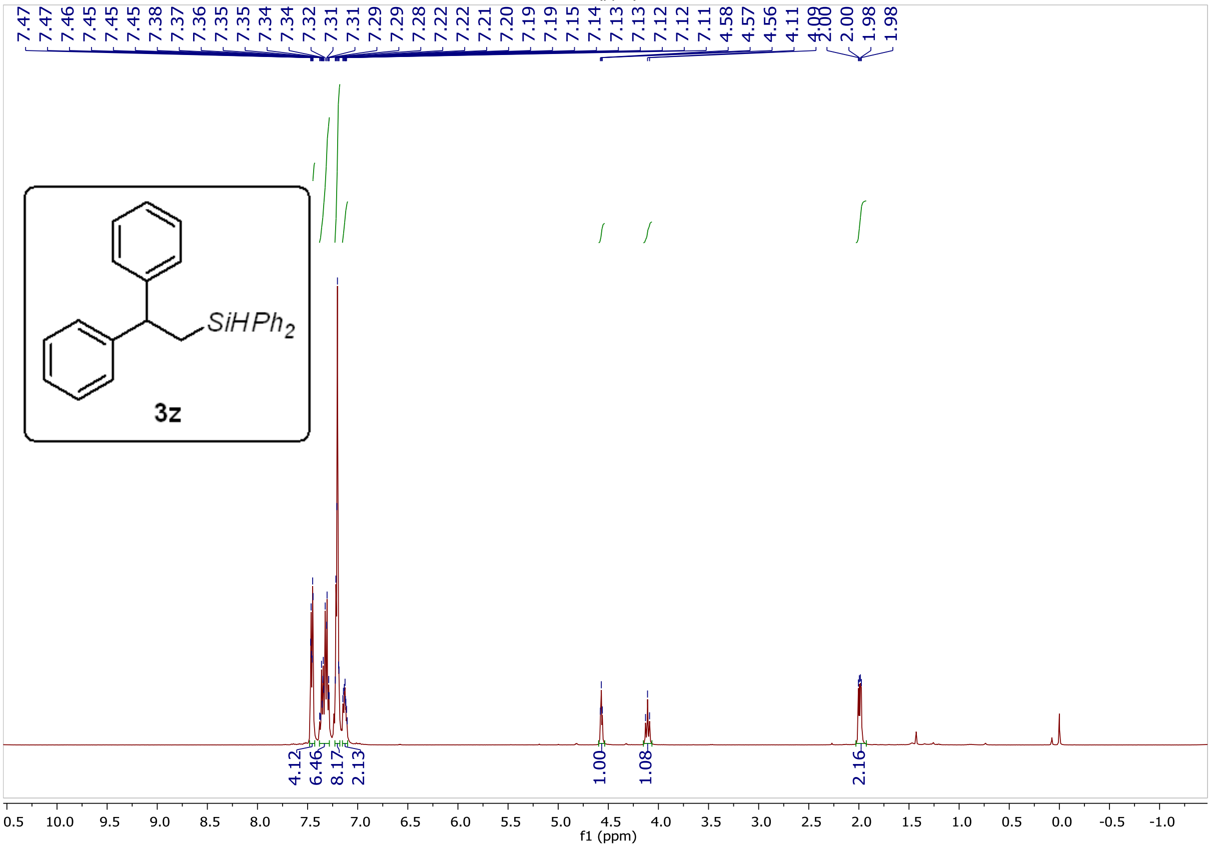

SiHPh2
3z
4.12
6.46
8.17
2.13
1.00
1.08
2.16
f1 (ppm)

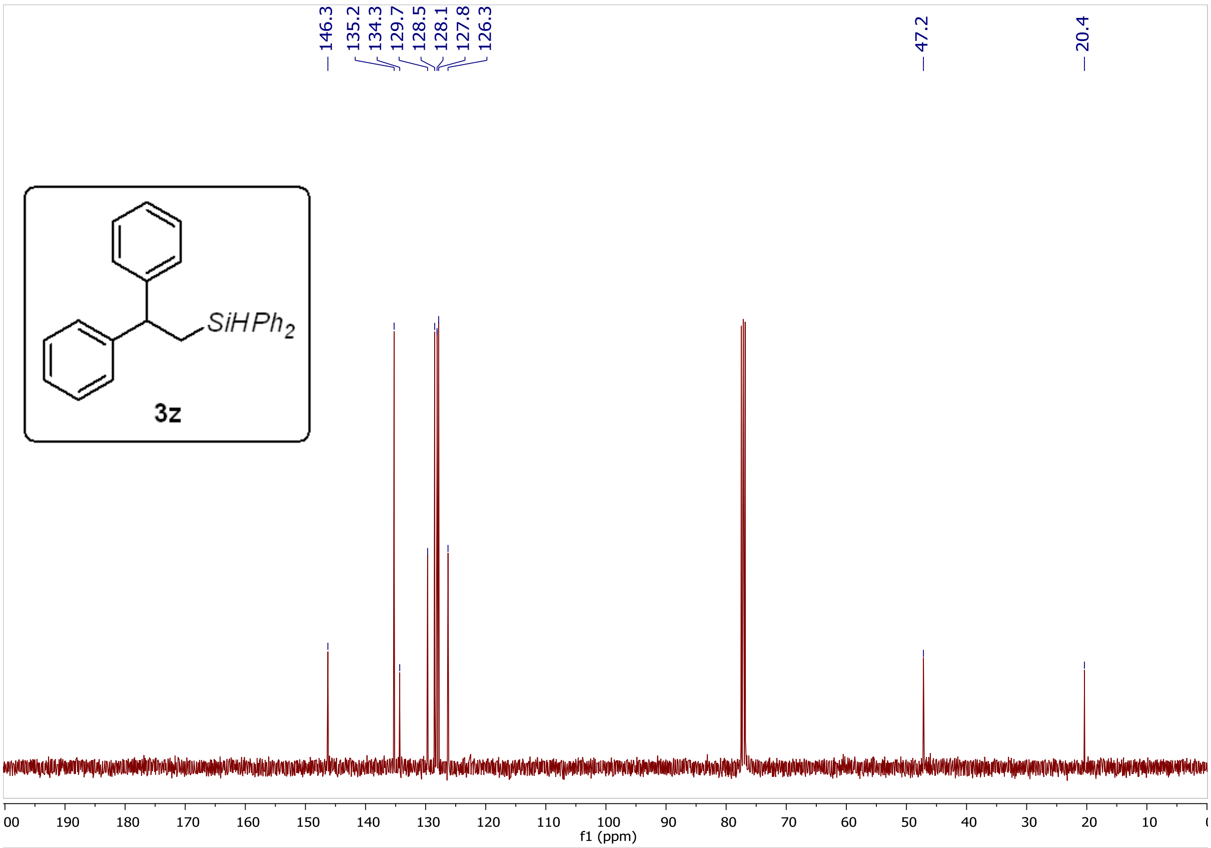
146.3
135.2
134.3
129.7
128.5
128.1
127.8
126.3
47.2
20.4
SiHPh2
3z
f1 (ppm)

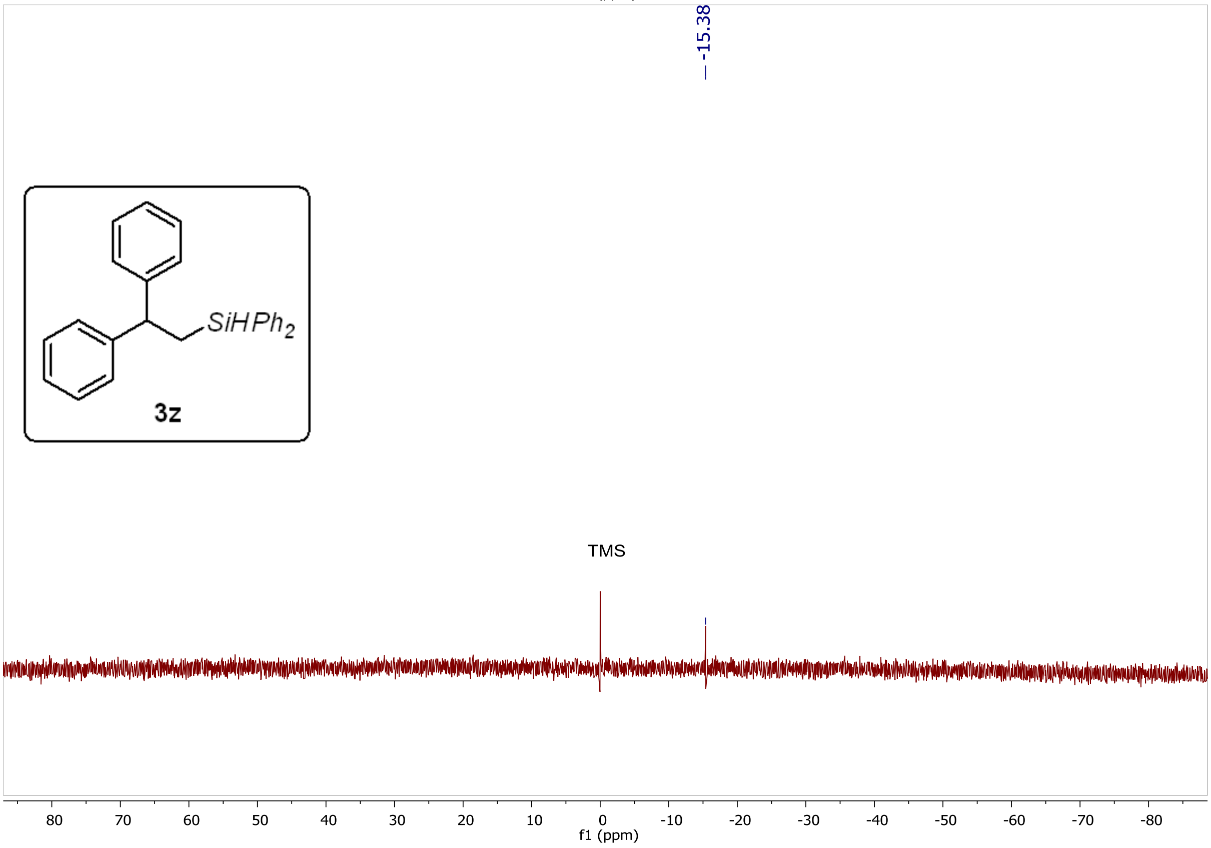
-15.38
SiHPh2
3z
TMS
f1 (ppm)

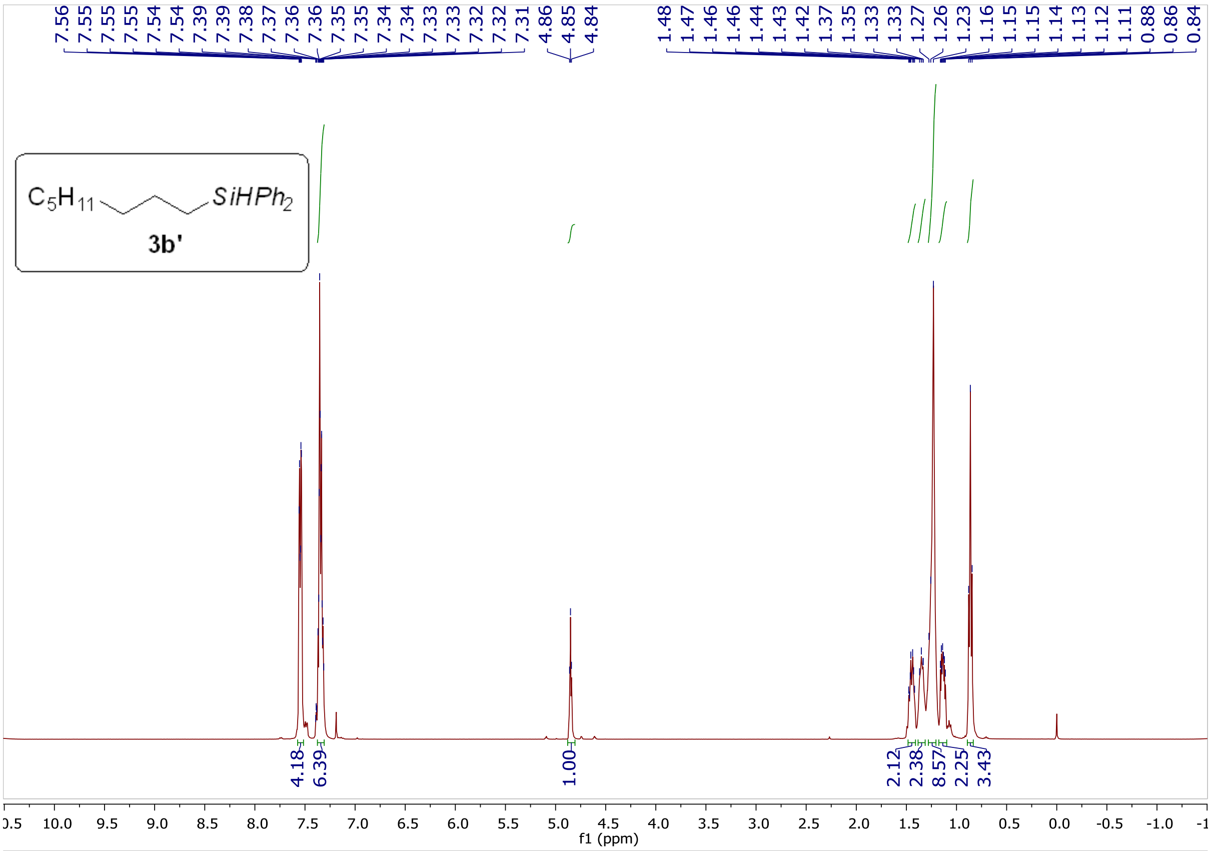
7.56 7.55 7.55 7.55 7.54 7.54 7.39 7.39 7.38 7.37 7.36 7.36 7.35 7.35 7.34 7.34 7.33 7.33 7.32 7.32 7.31 4.86 4.85 4.84 1.48 1.47 1.46 1.46 1.44 1.43 1.42 1.37 1.35 1.33 1.33 1.27 1.26 1.23 1.16 1.15 1.15 1.14 1.13 1.12 1.11 0.88 0.86 0.84
C_5H_{11} SiHPh$_2$
3b'
4.18 6.39 1.00 2.12 2.38 8.57 2.25 3.43
f1 (ppm)

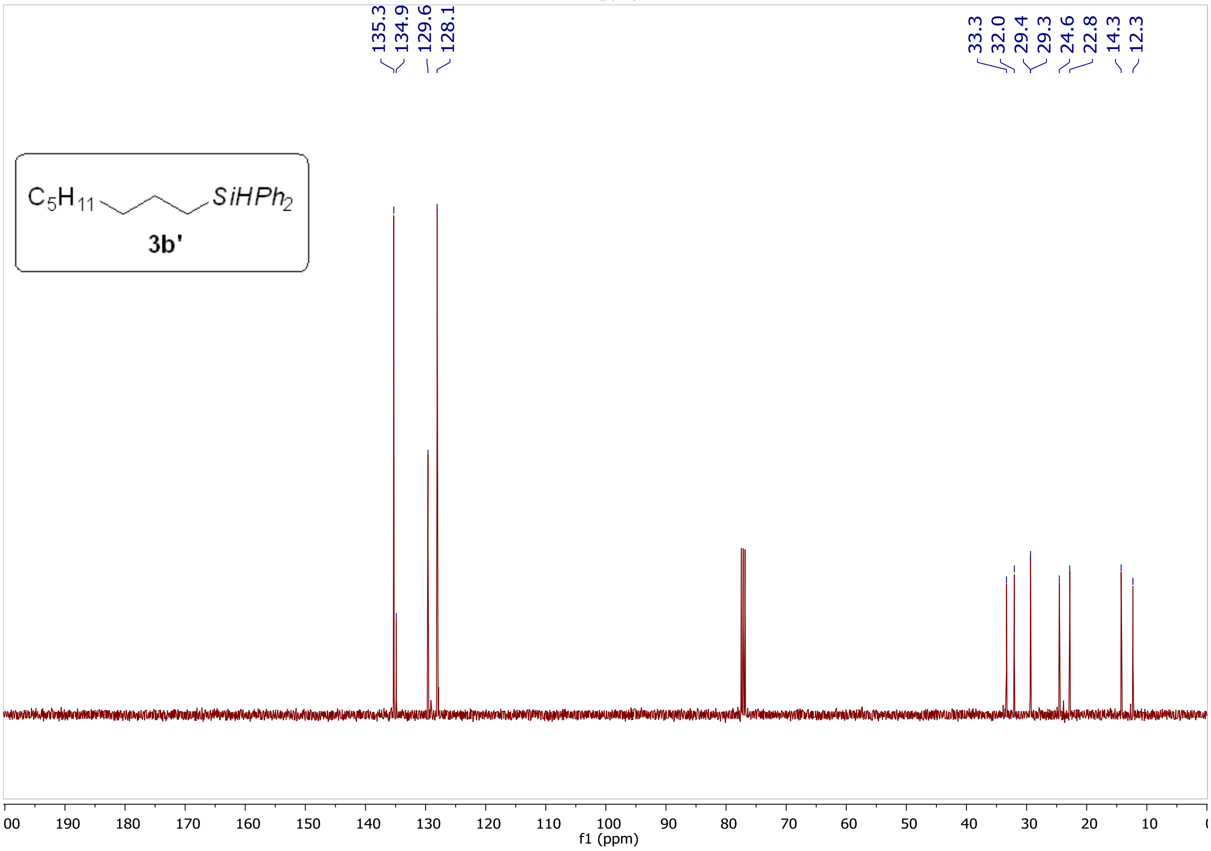
135.3 134.9 129.6 128.1 33.3 32.0 29.4 29.3 24.6 22.8 14.3 12.3
C_5H_{11} SiHPh$_2$
3b'
f1 (ppm)

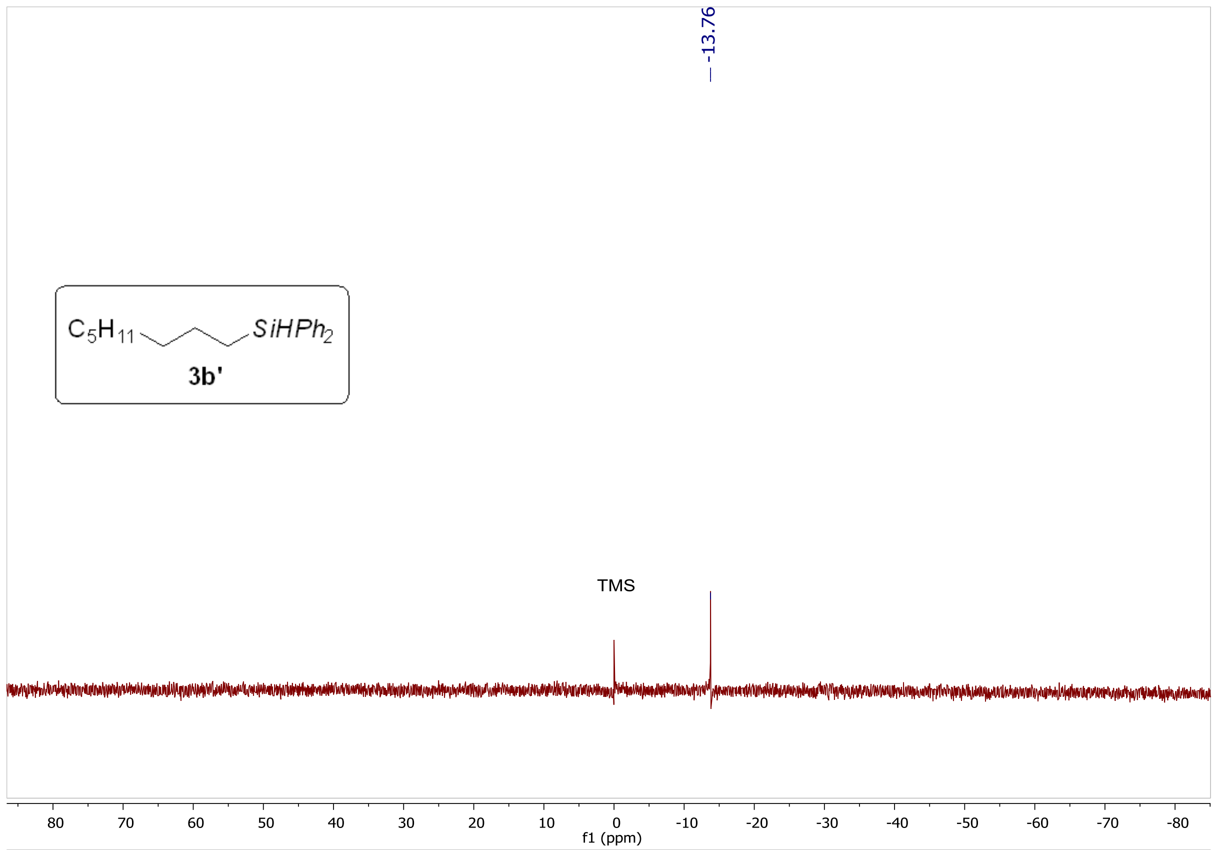

-13.76
TMS
C_5H_{11} SiHPh2
3b'
80 70 60 50 40 30 20 10 0 -10 -20 -30 -40 -50 -60 -70 -80
f1 (ppm)

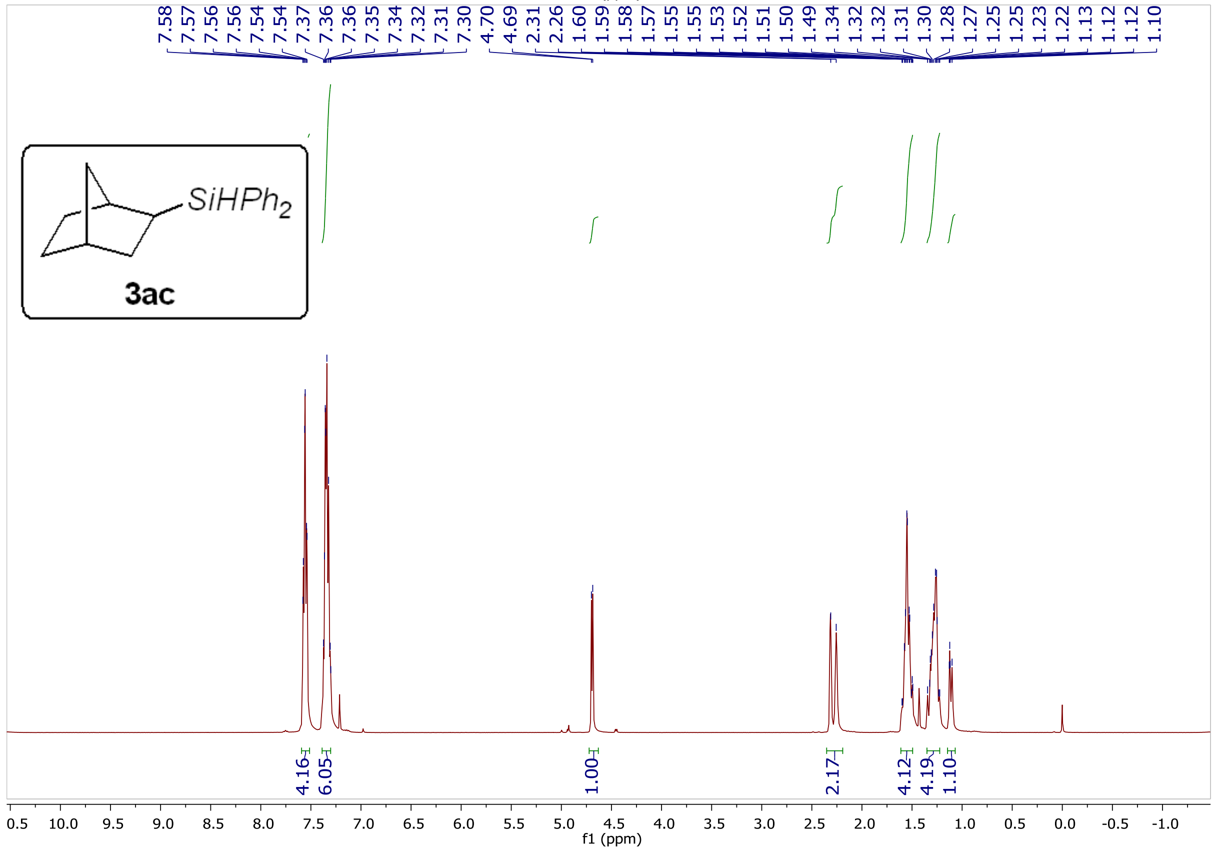

7.58 7.57 7.56 7.56 7.54 7.54 7.37 7.36 7.36 7.35 7.34 7.32 7.31 7.30 4.70 4.69 2.31 2.26 1.60 1.59 1.58 1.57 1.55 1.55 1.53 1.52 1.51 1.50 1.49 1.34 1.32 1.32 1.31 1.30 1.28 1.27 1.25 1.25 1.23 1.22 1.13 1.12 1.12 1.10
SiHPh2
3ac
4.16 6.05 1.00 2.17 4.12 4.19 1.10
0.5 10.0 9.5 9.0 8.5 8.0 7.5 7.0 6.5 6.0 5.5 5.0 4.5 4.0 3.5 3.0 2.5 2.0 1.5 1.0 0.5 0.0 -0.5 -1.0
f1 (ppm)

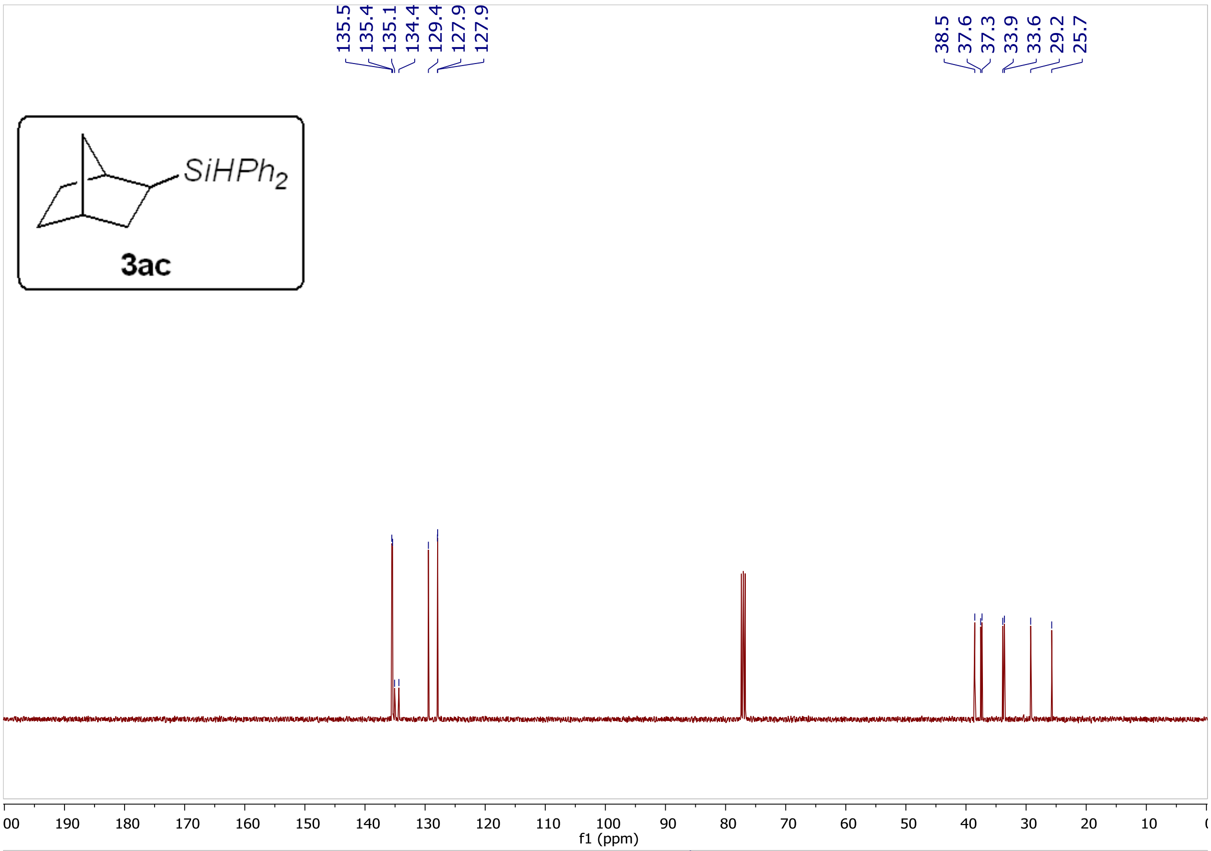
135.5
135.4
135.1
134.4
129.4
127.9
127.9
38.5
37.6
37.3
33.9
33.6
29.2
25.7
SiHPh2
3ac
f1 (ppm)

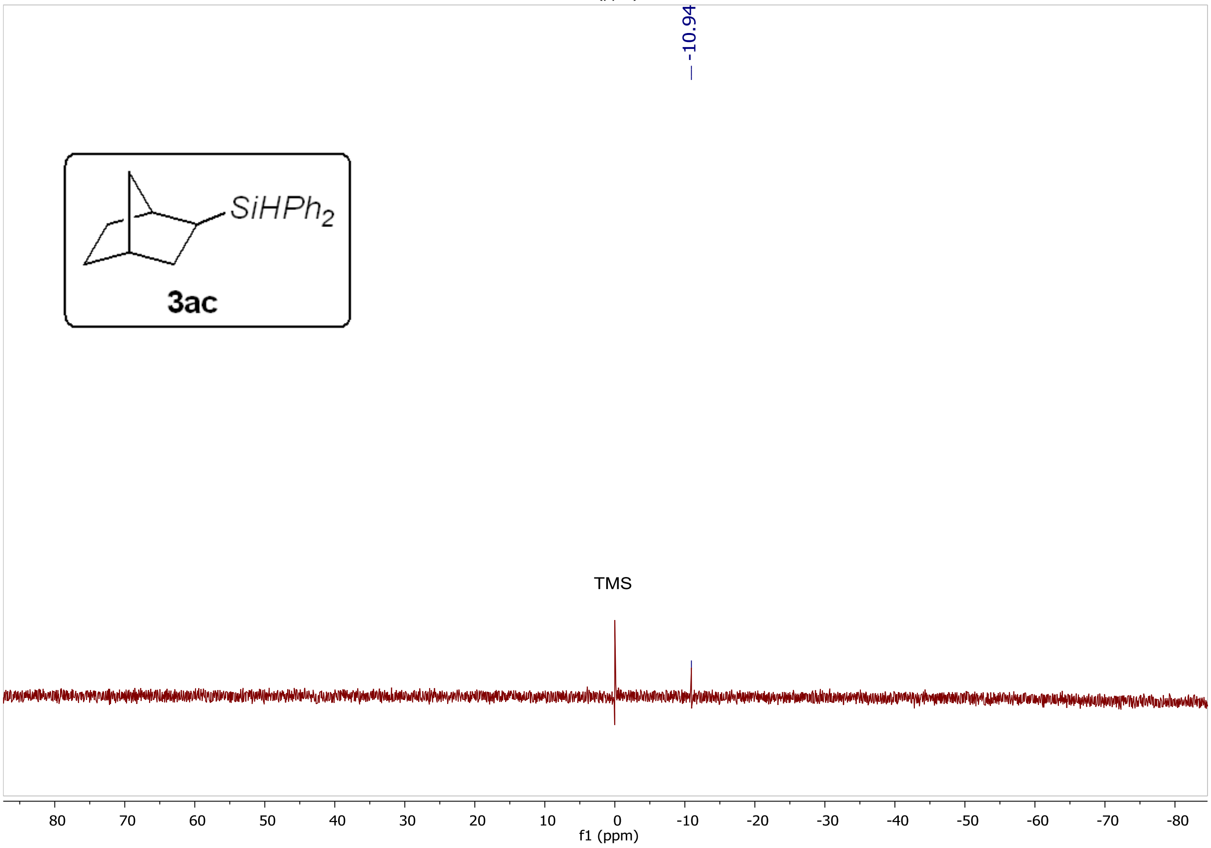
-10.94
SiHPh2
3ac
TMS
f1 (ppm)

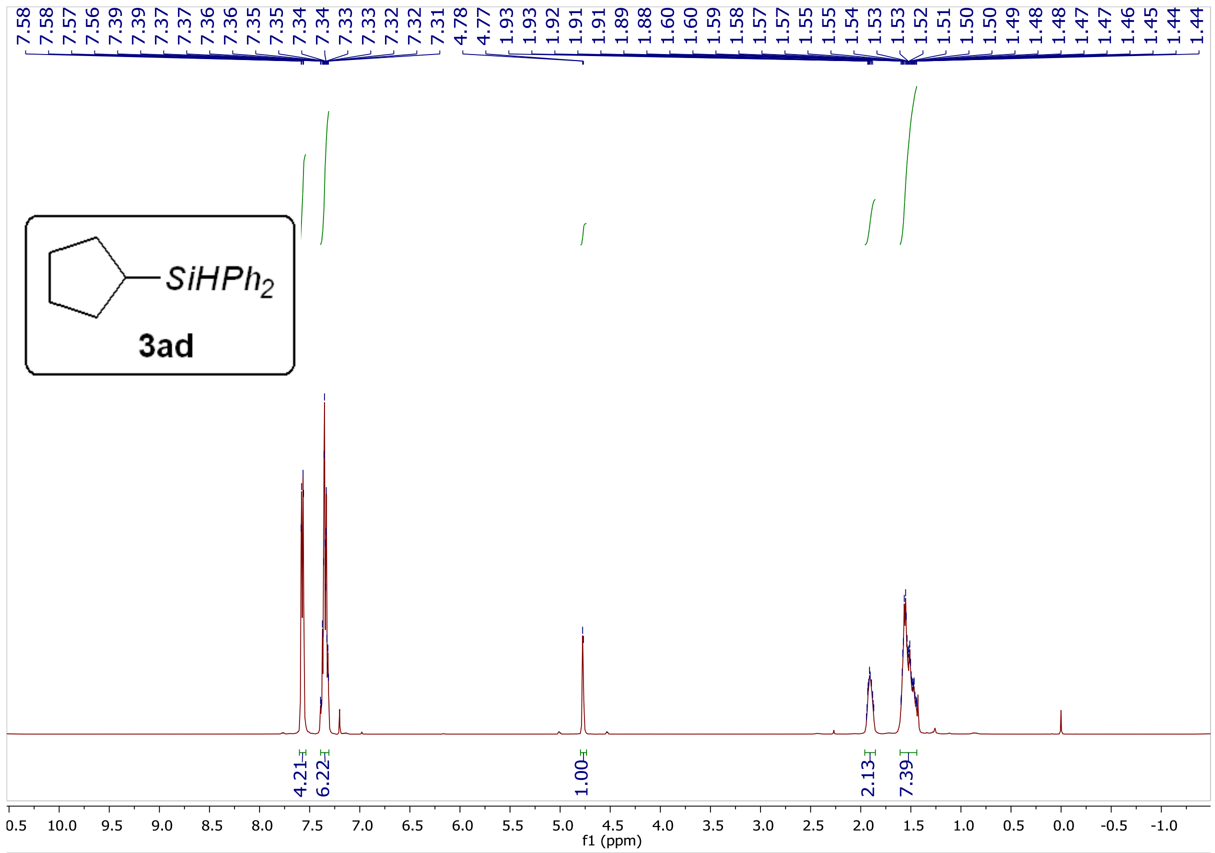
7.58
7.58
7.57
7.56
7.39
7.39
7.37
7.37
7.36
7.36
7.35
7.35
7.34
7.34
7.33
7.33
7.32
7.32
7.31
4.78
4.77
1.93
1.93
1.92
1.91
1.91
1.89
1.88
1.60
1.60
1.59
1.58
1.57
1.57
1.55
1.55
1.54
1.53
1.53
1.52
1.51
1.50
1.50
1.49
1.48
1.48
1.47
1.47
1.46
1.45
1.44
1.44
SiHPh2
3ad
4.21
6.22
1.00
2.13
7.39
f1 (ppm)

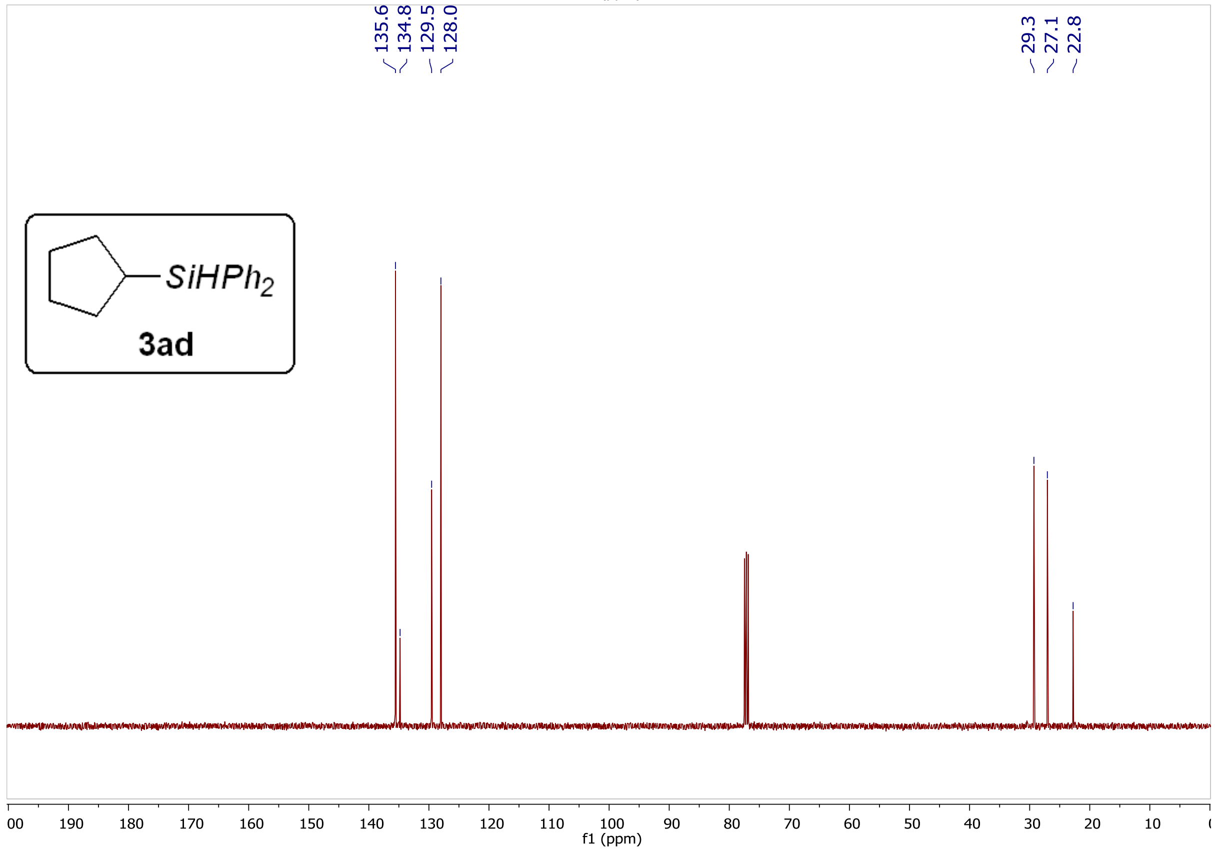
135.6
134.8
129.5
128.0
29.3
27.1
22.8
SiHPh2
3ad
f1 (ppm)

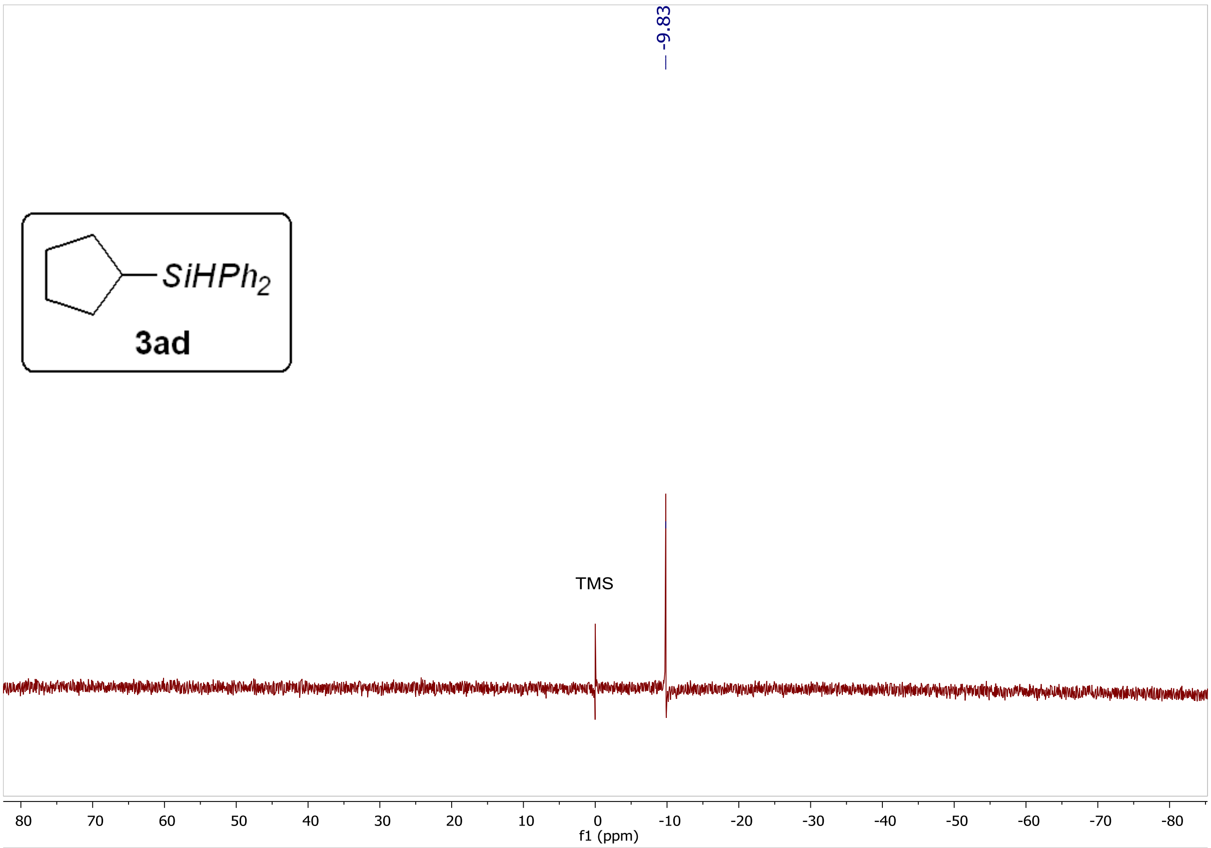
-9.83
SiHPh2
3ad
TMS
f1 (ppm)

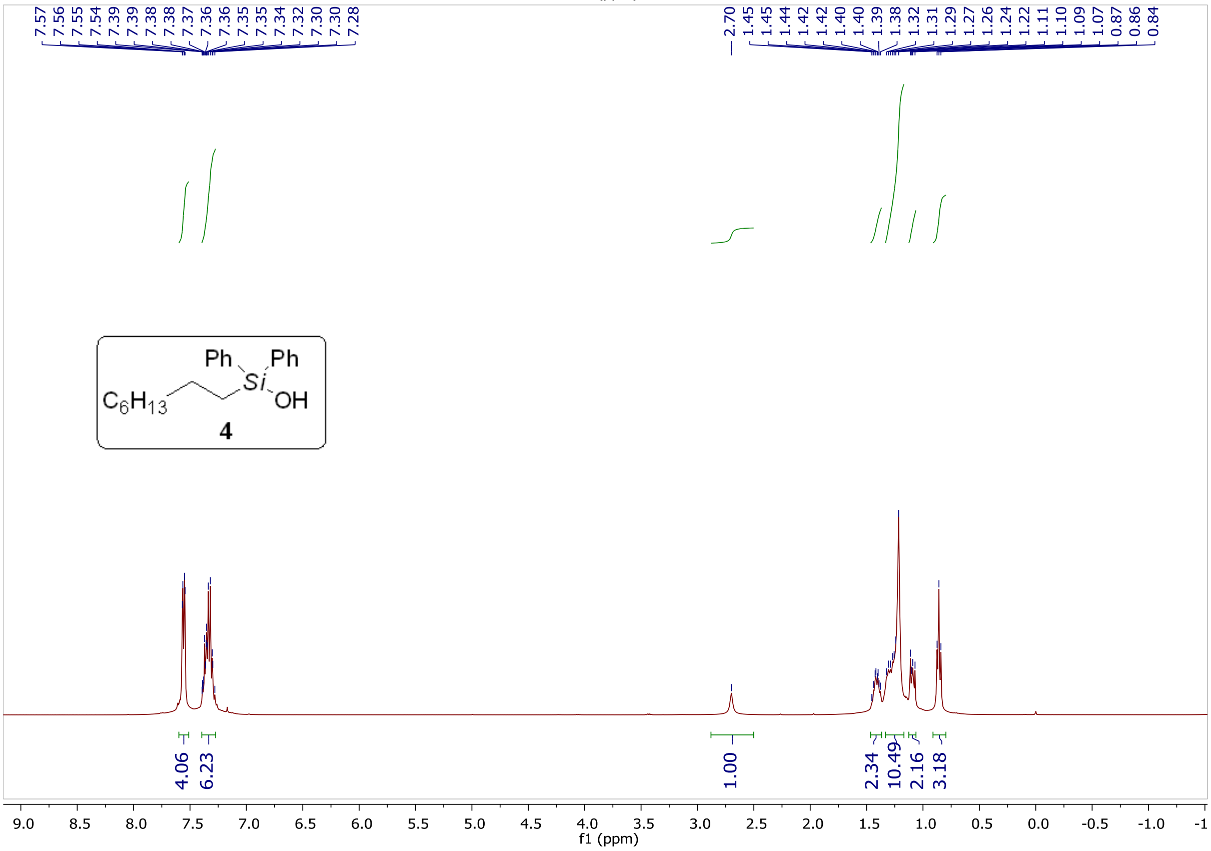
7.57 7.56 7.55 7.54 7.39 7.39 7.38 7.38 7.37 7.36 7.36 7.35 7.35 7.34 7.32 7.30 7.30 7.28
2.70 1.45 1.45 1.44 1.42 1.42 1.40 1.40 1.39 1.38 1.32 1.31 1.29 1.27 1.26 1.24 1.22 1.11 1.10 1.09 1.07 0.87 0.86 0.84
Ph Ph
Si
C6H13
OH
4
4.06 6.23 1.00 2.34 10.49 2.16 3.18
f1 (ppm)

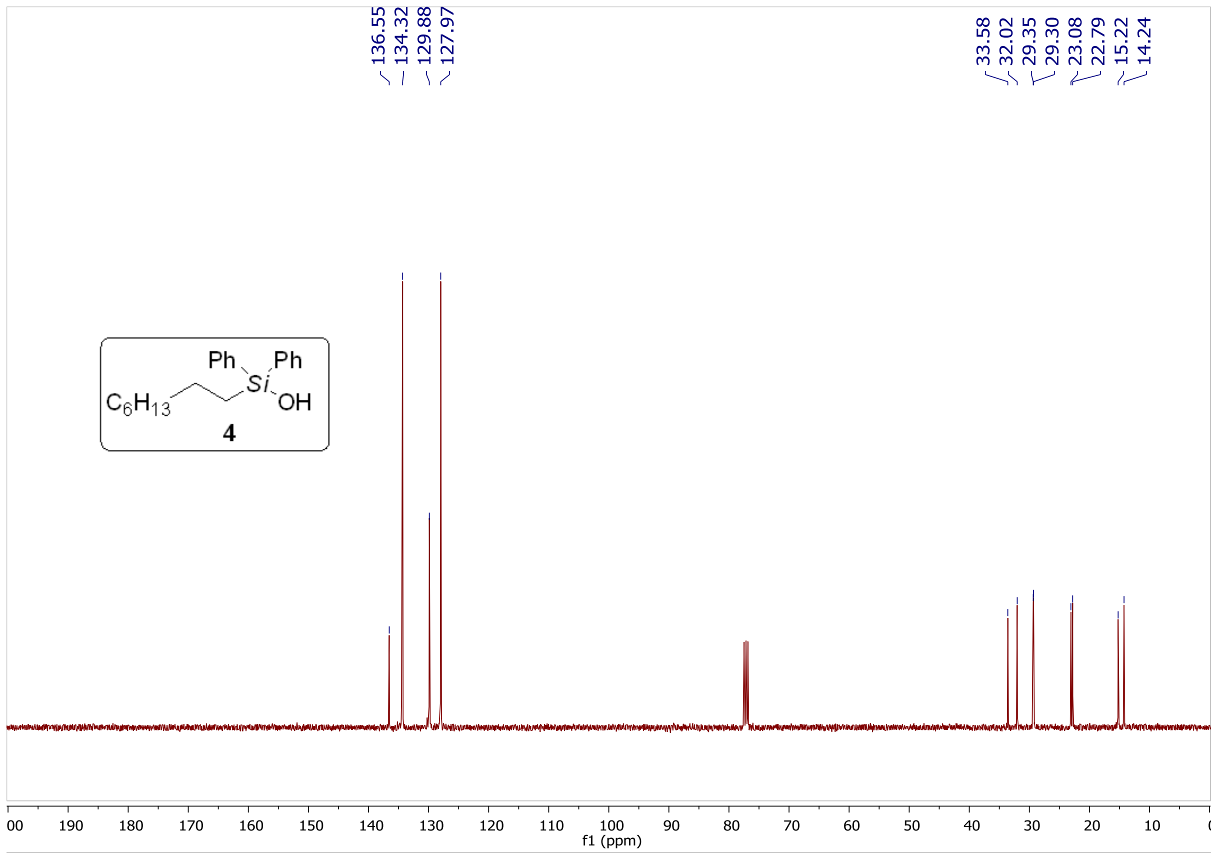
136.55
134.32
129.88
127.97
33.58
32.02
29.35
29.30
23.08
22.79
15.22
14.24
Ph
Ph
Si
C_6H_{13}
OH
4
f1 (ppm)

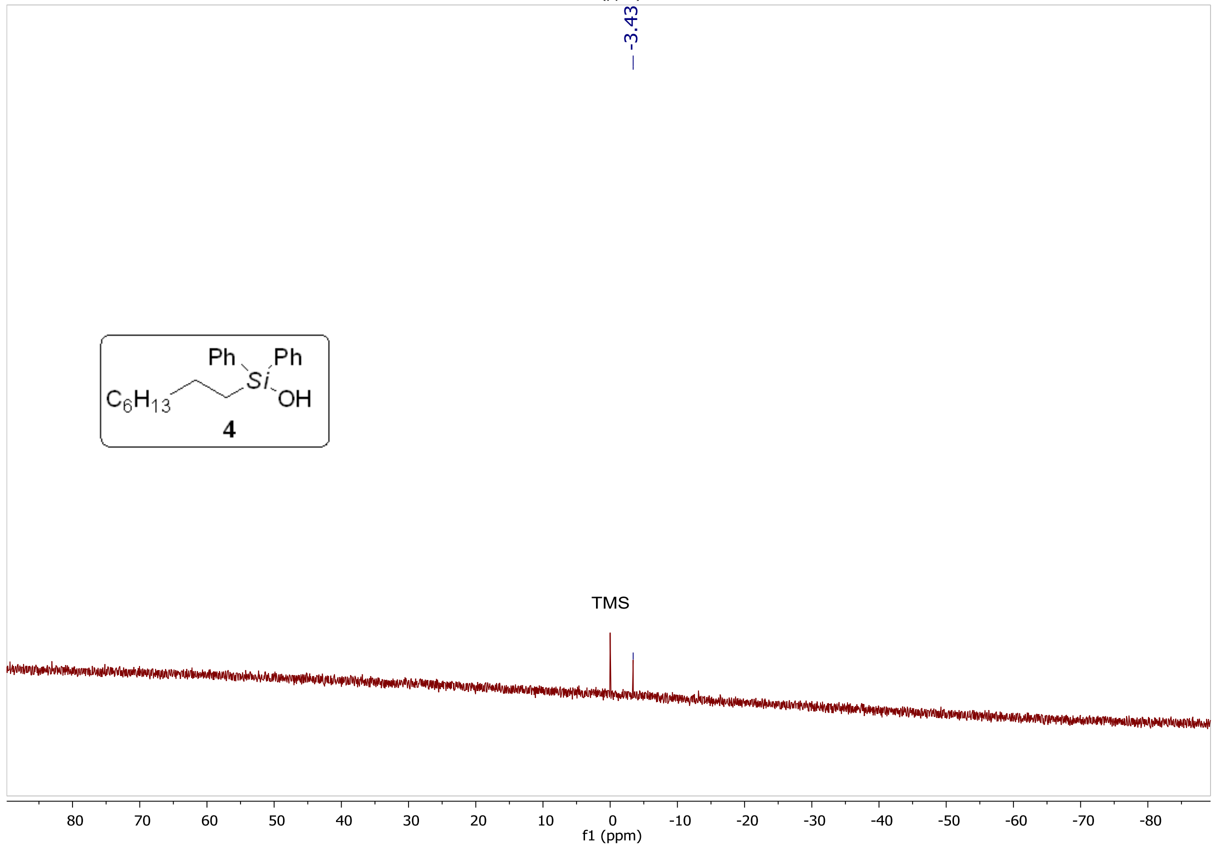
-3.43
Ph
Ph
Si
C_6H_{13}
OH
4
TMS
f1 (ppm)

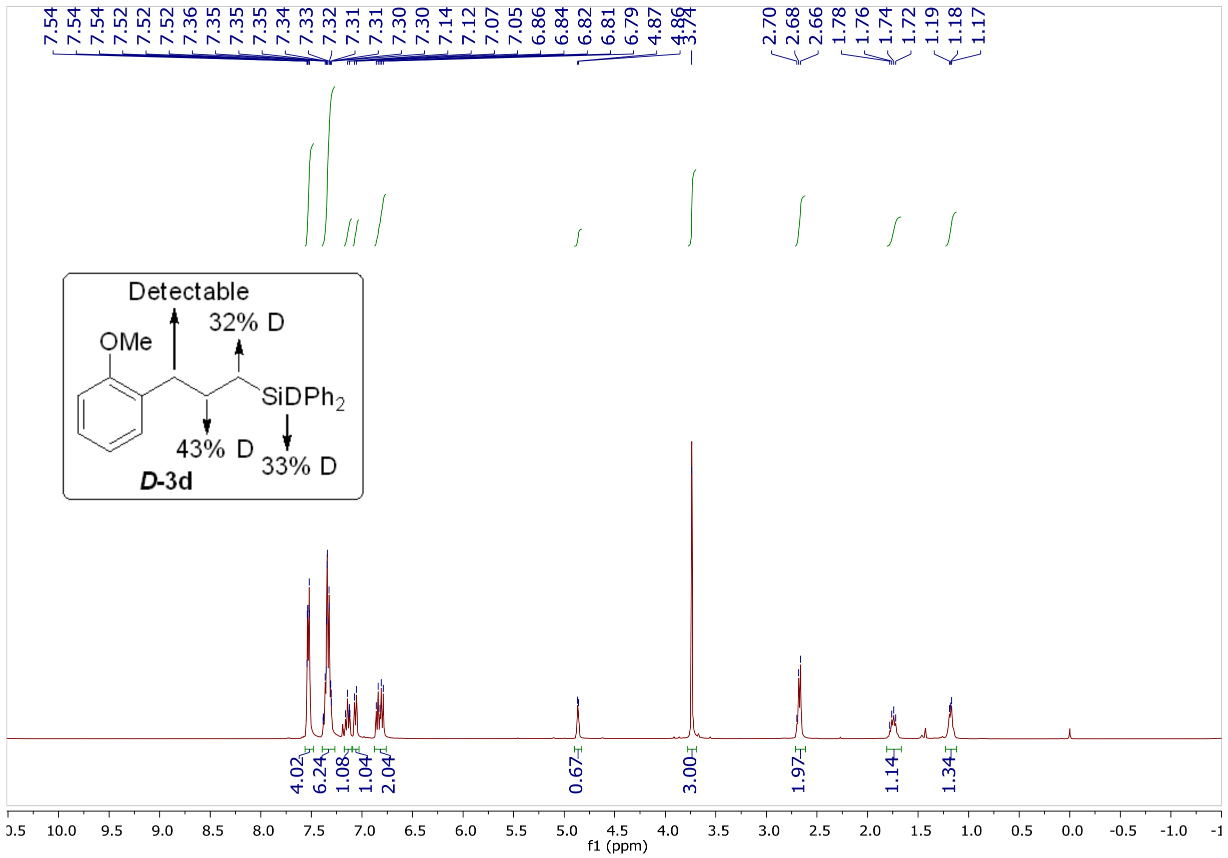
Detectable
32% D
OMe
SiDPh2
43% D
33% D
D-3d
f1 (ppm)

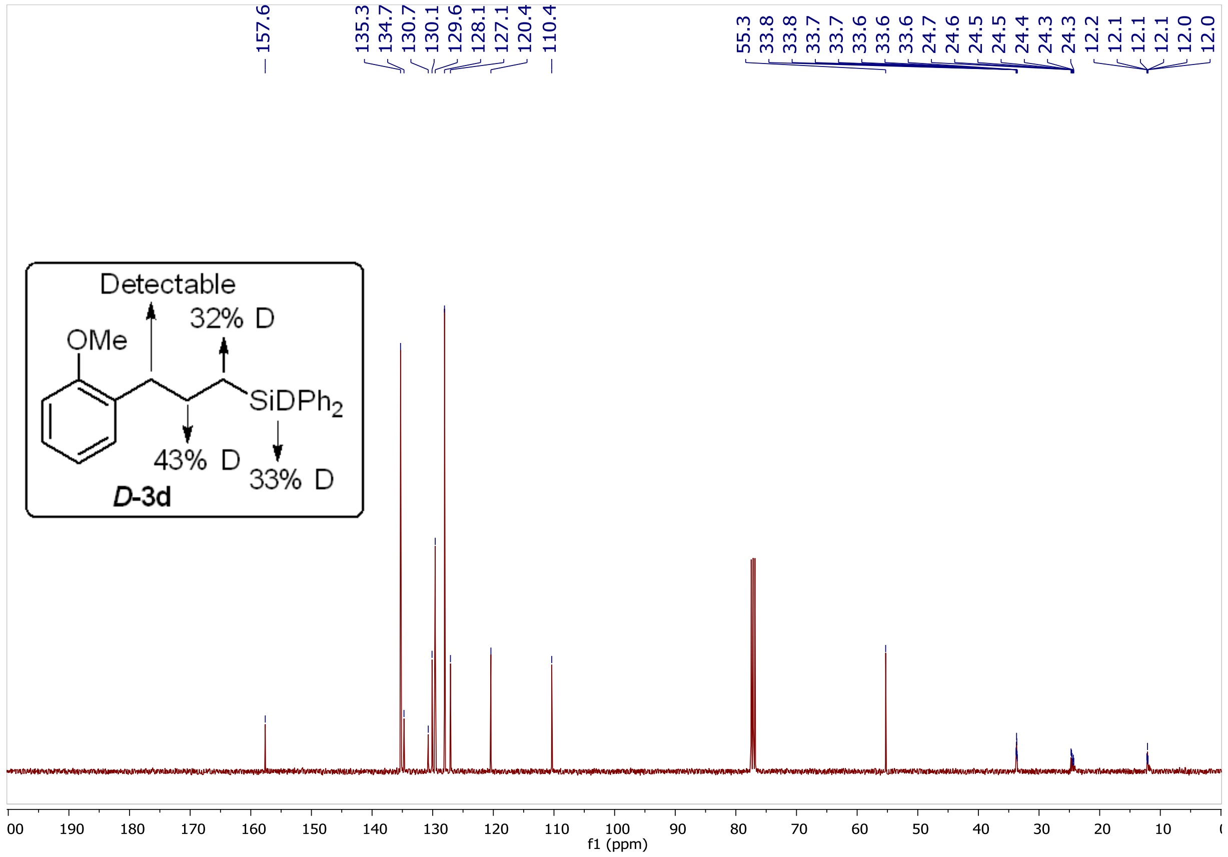
Detectable
32% D
OMe
SiDPh2
43% D
33% D
D-3d
f1 (ppm)

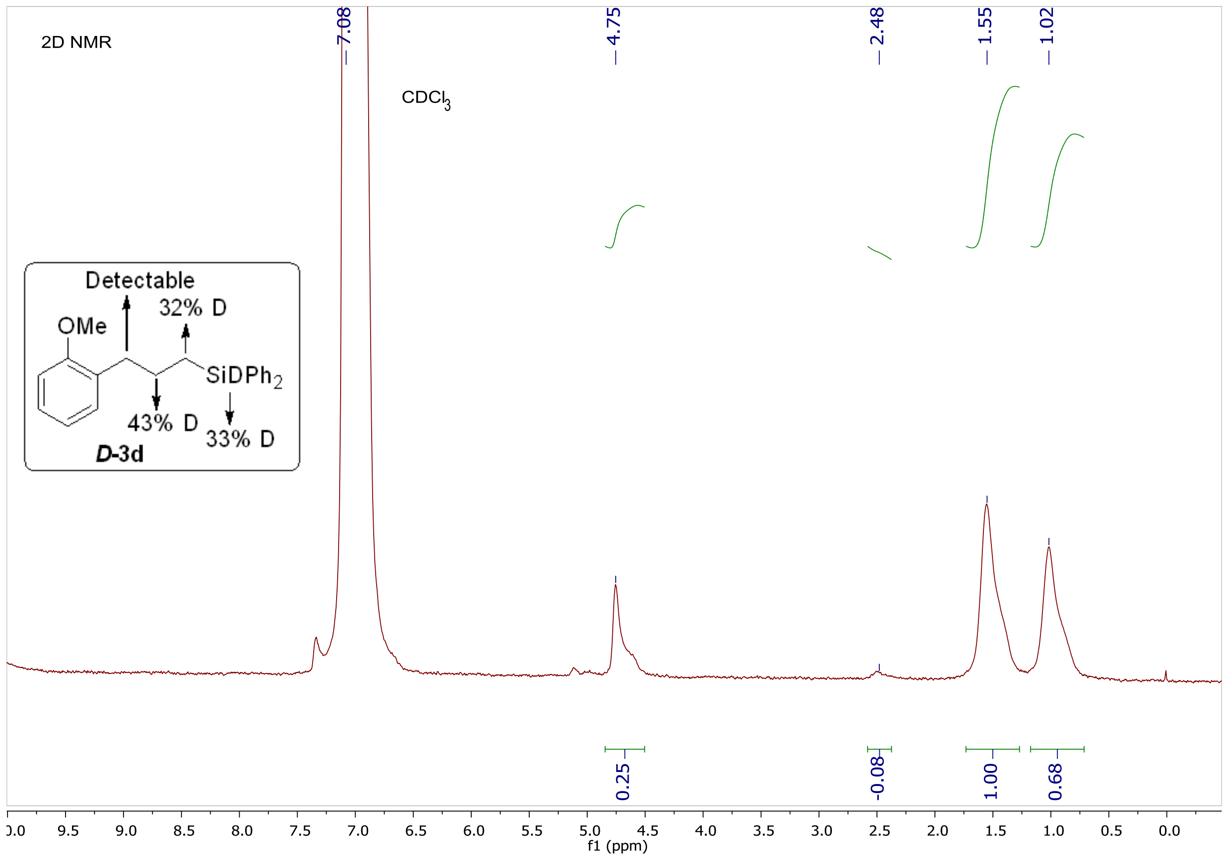
2D NMR
CDCl3
7.08
4.75
2.48
1.55
1.02
Detectable
32% D
OMe
SiDPh2
43% D
33% D
D-3d
0.25
-0.08
1.00
0.68
f1 (ppm)
